# Comparative Genomics and Pathogenicity Analysis of Three Fungal Isolates Causing Barnyard Grass Blast

**DOI:** 10.3390/jof10120868

**Published:** 2024-12-13

**Authors:** Na Zhang, Xinyang Li, Liangping Ming, Wenda Sun, Xiaofang Xie, Cailing Zhi, Xiaofan Zhou, Yanhua Wen, Zhibin Liang, Yizhen Deng

**Affiliations:** 1 State Key Laboratory for Conservation and Utilization of Subtropical Agro-Bioresources, Guangdong Province Key Laboratory of Microbial Signals and Disease Control, Integrative Microbiology Research Centre, South China Agricultural University, Guangzhou 510642, China; nzhang0206@163.com (N.Z.); mg210914@163.com (L.M.); dadasun34@gmail.com (W.S.); 13925429932@163.com (X.X.); zhicailing2022@163.com (C.Z.); zhouxiaofan1983@163.com (X.Z.); yhwen@scau.edu.cn (Y.W.); 2 State Key Laboratory for Conservation and Utilization of Subtropical Agro-Bioresources, Guangxi Key Laboratory of Sugarcane Biology, Guangxi University, Nanning 530004, China; 17309879162@163.com; 3 Guangdong Laboratory for Lingnan Modern Agriculture, Guangzhou 510642, China

**Keywords:** plant pathogenic fungi, whole-genome sequencing, avirulence genes, comparative genomics

## Abstract

Barnyard grass is one of the most serious rice weeds, often growing near paddy fields and therefore potentially serving as a bridging host for the rice blast fungus. In this study, we isolated three fungal strains from diseased barnyard grass leaves in a rice field. Using a pathogenicity assay, we confirmed that they were capable of causing blast symptoms on barnyard grass and rice leaves to various extents. Based on morphology characterization and genome sequence analyses, we confirmed that these three strains were *Epicoccum sorghinum* (SCAU-1), *Pyricularia grisea* (SCAU-2), and *Exserohilum rostratum* (SCAU-6). The established Avirulence (*Avr*) genes *Avr-Pia*, *Avr-Pita2*, and *ACE1* were detected by PCR amplification in SCAU-2, but not in SCAU-1 or SCAU-6. Furthermore, the whole-genome sequence analysis helped to reveal the genetic variations and potential virulence factors relating to the host specificity of these three fungal pathogens. Based on the evolutionary analysis of single-copy orthologous proteins, we found that the genes encoding glycoside hydrolases, carbohydrate esterases, oxidoreductase, and multidrug transporters in SCAU-1 and SCAU-6 were expanded, while expansion in SCAU-2 was mainly related to carbohydrate esterases. In summary, our study provides clues to understand the pathogenic mechanisms of fungal isolates from barnyard grass with the potential to cause rice blast.

## 1. Introduction

Species of *Pyricularia* are responsible for major diseases in grasses, among which *Pyricularia oryzae* (syn. *Magnaporthe oryzae*) is responsible for the major disease in rice (*Oryza sativa* L.) [1] and other important crops including wheat (*Triticum aestivum* L.), barley (*Hordeum vulgare*), and millet (*Panicum miliaceum* L.) [2]. On the other hand, *Pyricularia grisea* (syn. *Magnaporthe grisea*) is responsible for foliar diseases in crabgrass (*Digitaria sanguinalis*). *P. grisea* isolates from crabgrass usually only infect crabgrass but not other hosts, while *P. oryzae* isolates from rice and other grasses and some *P. grisea* isolates from crabgrass are capable of infecting rice [3,4]. The host shift and host expansion of fungal plant pathogens increase the frequency of new pathogens and the incidence of diseases in various crops, which poses a threat to global food security. The outbreaks of wheat blast in Brazil and gray leaf spot in perennial ryegrass (*Lolium perenne* L.) in Pennsylvania in the 20th century are important pieces of evidence of the host shift of *P. oryzae* [5,6]. It was speculated that the *Lolium* isolates may contribute to the evolution of the wheat-infecting strain by jumping from *Lolium* spp. to wheat as the host plant [4,7]. In addition, a study suggests that *P. oryzae* may shift from millet (*Setaria* spp.) to rice [8]. Recently, researchers have discovered a new epidemic caused by *P. oryzae* in maize (*Zea mays*), and comparative genomics analysis suggests that the pathogen may originate from barnyard grass (*Echinochloa* spp.) [9]. In addition, the fungal pathogens *Epicoccum sorghinum* and *Exserohilum rostratum* also have a wide host range. *E. sorghinum* can infect maize [10], daylily (*Hemerocallis citrina*) [11], *Dendrobium officinale* [12], tea (*Camellia sinensis*) [13], *Digitaria sanguinalis* [14], lavender (*Lavandula stoechas*) [15], bayberry (*Myrica species*) [16], etc. *E. sorghinum* can not only independently cause plant diseases but also can synergistically infect plants with other fungi [17,18]. *E. sorghinum* can synthesize a mycotoxin called Tenuazonic acid (TeA), which is toxic to plants and animals [19,20]. In addition to the mycotoxin, *E. sorghinum* can also secrete a polyglycine hydrolase (Es-cmp), which can specifically cleave plant class IV chitinases containing polyglycine structures [21]. *E. rostratum* is a pathogen with strains causing plant or human disease [22]. The plant hosts of *E. rostratum* include more than 30 plant species across 11 families and 28 genera, e.g., maize [23], sugarcane (*Saccharum sinensis*) [24], broccoli (*Brassica oleracea* var. *italica*) [25], banana (*Musa paradisiaca*) [26], tomato (*Solanum lycopersicum*) [27], and wheat [28]. In recent years, rice leaf spot caused by *E. sorghinum* and rice brown spot caused by *E. rostratum* have been reported [29,30]. However, there are very few reports on how *E. sorghinum* and *E. rostratum* shift between hosts and cause diseases in rice. Surveillance of host shift and/or expansion of rice pathogenic fungi based on comparative genomic analysis may be beneficial to predict and prevent the epidemiology of rice diseases.

The genomes of pathogenic strains of *Pyricularia* contain more secretory proteins encoding genes and Avirulent (*Avr*) genes than those of the non-pathogenic strains, which may account for the successful infection of the host plants [31]. The interaction between the *Avr* genes of the fungal pathogen and the resistance (*R*) genes of the plant host controls the host specificity of plant pathogens, according to the classical gene-to-gene theory [32]. The interaction between rice and the blast fungus conforms to the classical gene-to-gene theory. A total of 26 *Avr* genes have been identified in *P. oryzae*, and 14 of the corresponding *R* genes have been cloned in rice [33]. *Avr* gene variations cause the virulence variation of *P. oryzae* strains. Gene deletions, frame-shift mutations, and transposon insertions, etc., may change the function of *Avr* genes, thus affecting the pathogenicity of the strains carrying such *Avr* genes [34,35]. In addition, the existence of some specific gene families in the genome, such as gene families encoding carbohydrate active enzymes (CAZys) or those involved in secondary metabolism, may adapt pathogens to different hosts [36]. For instance, a comparison of the genomes of *Botryosphaeria dothidea* and *B. kuwatsukai* revealed that *B. kuwatsukai*, which can only infect apples and pears, has lost several genes encoding secondary metabolites, carbohydrate enzymes, and plant cell wall-degrading enzymes [37]. Analysis of the genomes of two closely related *Colletotrichum* species with diverged hosts, *Colletotrichum graminicola* and *C. sublineola,* showed the differences in their genes encoding secondary metabolites and small secreted proteins [38]. Additionally, a previous study indicated that *Colletotrichum* strains, which can cause plant diseases but have a narrow host range, have undergone a contraction in the gene families encoding carbohydrate-active enzymes and proteolytic enzymes, while strains with broad host ranges exhibit specific expansions [39]. At present, genomic information on rice-infecting *Pyricularia* strains is abundantly available, but less reported in the barnyard grass-infecting *Pyricularia* strain(s).

Barnyard grass is a widely distributed weed and could potentially serve as a bridging host for the rice blast fungus, aiding in the survival and dispersal of rice pathogens. In this study, we isolated three fungal strains from the blast lesion of barnyard grass growing near a rice field. We tested the pathogenicity of these three strains on detached leaves of barnyard grass and rice. We also detected the presence/absence of several well-established *Pyricularia Avr* genes in these three strains using PCR amplification and DNA sequencing. In addition, we sequenced the whole genome of the three strains in order to further study their classification and pathogenic mechanism at the genome level. Overall, our results provided new insights into the evolutionary relationship between barnyard grass-infecting and rice-infecting fungal strains.

## 2. Materials and Methods

### 2.1. Isolation of Fungal Strains

In 2022, barnyard grass leaves with blast symptoms were collected from the experimental farm of South China Agricultural University (113.36° E, 23.16° N), and the fungal strains were isolated according to the conventional tissue isolation method [40]. The single colony was obtained using the single-spore isolation method [41]. After the isolated strains were cultured under the same conditions, the morphology of the hyphae, conidiophores, and conidia of fungal strains were observed under a light microscope and the sizes of conidia were measured for morphological classification [42,43,44]. These three fungal strains were routinely grown at 25 ± 2 °C and 50 ± 5% relative humidity.

### 2.2. Pathogenicity Assay

The mycelial plugs or conidial suspensions (10^5^/mL unless stated otherwise) of the isolated strains were inoculated on the detached leaves of barnyard grass (*Echinochloa phyllopogon*) or susceptible rice cultivars (Nipponbare, CO39, and LTH) [45,46]. The inoculated leaves were kept in a dark incubator at 25 °C and 90% relative humidity for 24 h, then transferred to an incubator with light/dark (12 h/12 h) cycling for 4–5 days before disease symptoms were recorded and photographed [47]. Three independent biological repeats were performed.

### 2.3. Genomic DNA Extraction, Amplification, and Sequencing

The fungal strains were inoculated in Potato Dextrose Agar (PDA) medium and cultured in a 25 °C incubator for 7–10 days. The mycelia were collected for genomic DNA extraction by the CTAB method [48]. PCR amplification with the ITS1/ITS4 primer was performed, and the amplified product was sent to Sangon Biotech (Shanghai) Co., Ltd. (Shanghai, China) for sequencing. Sequence alignment was performed by Clustal X2 Version 2.1 [49] and dashed by GeneDoc Version 2.7 [50]. The phylogenetic tree of ITS was constructed using the neighbor-joining method (NJ) with MEGA7 Version 7.0.26 [51] software based on the amplified ITS fragment sequences. The phylogenetic tree of ACE1 proteins was constructed using the maximum likelihood method based on the best-fit JTT matrix-based model selected by MEGA 7, with 1000 bootstrap support. For the detection of the selected *Avr* genes, primers were designed using reference gene sequences (Table S1).

The whole genome was sequenced, via massively parallel sequencing (MPS) on the Illumina NovaSeq 6000 platform, by Sangon Biotech (Shanghai) Co., Ltd.

### 2.4. Assembly, Prediction, and Annotation

SPAdes Version 3.5.0 software [52] was used to perform de novo genome-wide assembly of the quality-controlled sequences (parameters: -k 31, 51, 71, 91). Replicates were performed to ensure consistency in genome assembly. The assembled data were evaluated by BUSCO Version 5.4.6 [53]. FastANI Version 1.33 software was used to calculate the ANI value between the sequenced genome and the published genomes of the closely related species in order to evaluate the phylogenetic relationships of the above species at the whole-genome level [54]. The assembly results were used for de novo genome prediction with GeneMark Version 1.10, which employs an unsupervised learning model to predict protein-coding genes in eukaryotes [55]. The tRNA was predicted by tRNAscan-SE Version 2.0 software, and the rRNA was predicted by rRNAmmer Version 1.2 software. The assembled complete genome was annotated by aligning it with the Rfam database (http://rfam.xfam.org/, accessed on 25 February 2023) using Infernal Version 1.1.4 software and its cmsearch program (with default parameters) to identify the final snRNA, miRNA, and sRNA [56]. For the prediction of repetitive sequences, RepeatModeler Version 2.0.3 software was used to construct a species-specific repeat library and identify the types of repetitive sequences within it. This library was then merged with the Repbase database (https://www.girinst.org/server/RepBase/index.php, accessed on 18 March 2023) to create the final repetitive sequence database. RepeatMasker Version 4.1.4 software was used to align the genomic sequences to this database to predict interspersed repeats. TRF Version 4.09.1 was used to predict tandem repeats [57].

The protein sequence of the target species was compared with COG (http://www.ncbi.nlm.nih.gov/COG/, accessed on 10 April 2023), GO (https://www.geneontology.org/, accessed on 15 April 2023), KEGG (https://www.kegg.jp/, accessed on 19 April 2023), TCDB (http://www.tcdb.org, accessed on 14 May 2023), Swiss-Prot (http://www.expasy.ch/sprot, accessed on 21 April 2023), Pfam (http://pfam.xfam.org/, accessed on 26 April 2023), CAZy (http://www.cazy.org, accessed on 1 May 2023), Cytochrome P450 (http://p450.riceblast.snu.ac.kr/intro.php, accessed on 15 May 2023), and other databases for BLAST Version 2.2.28 (Basic Local Alignment Search Tool) comparison (E-value 1E-5). We employed the PHI (http://www.phi-base.org/, accessed on 2 June 2023) and the Fungal Virulence Factor Database (DFVF) (http://sysbio.unl.edu/DFVF/, accessed on 10 June 2023) to predict pathogenic factors of the pathogen (E-value 1E-5; similarity >40%). SignalP Version 4.1.0 [58] software and TMHMM (https://services.healthtech.dtu.dk/services/TMHMM-2.0/, accessed on 20 June 2023) were used to forecast the proteins secreted from the classical pathway in the genome. Sequences with only signal peptide characteristics were then subjected to effector protein prediction using EffectorP Version 3.0.0. Finally, antiSMASH Version 7.0.0 software (https://fungismash.secondarymetabolites.org/, accessed on 10 July 2023) was utilized to identify secondary metabolite biosynthetic genes and gene clusters based on specified types of hidden Markov models, and BLAST analysis was conducted to determine the presence and absence of core secondary metabolism genes in the genome (parameters: E-value 1E-5, 75% coverage).

### 2.5. Phylogenetic Analysis and Comparative Genomic Analysis

OrthoFinder Version 2.5.5 [59] was used to perform cluster analysis of 3 isolated strains and 16 selected genomes downloaded from the NCBI Genome database (https://www.ncbi.nlm.nih.gov/genome, accessed on 17 August 2023). The extracted single-copy orthologous protein sequences were aligned using MUSCLE Version 5.1.0 for multiple sequence alignment [60]. Subsequently, a phylogenetic tree was constructed using the RAxML Version 8.2.12 software with the maximum likelihood (ML) method (Model: PROTGAMMALGF; Bootstrap: 1000). MCMCTree (MCMCTREE in paml version 4.10.0) was employed to estimate divergence times (references: *U*. *maydis* and *P. oryzae* 70-15: 583.2–749.0 MYA, *P. oryzae* Y34 and *E*. *rostratum*: 279.0–558.0 MYA; based on the estimation on http://www.timetree.org/, accessed on 30 August 2023), and based on the results of OrthoFinder Version 2.5.5, information on the number of gene families in each species was obtained. CAFÉ Version 5.0.0 was then utilized to identify gene family expansions and contractions. Additionally, six genomes (*P. oryzae* 70-15, *P. oryzae* B71, *P. grisea* NI907, SCAU-1, SCAU-2, and SCAU-6) were selected for cluster analysis to identify the unique gene families and common gene families in the six genomes.

Identification of positively selected genes was conducted across four genomes, *P. oryzae* 70-15 (GenBank accession no. GCA_000002495.2), *P. oryzae* B71 (GenBank accession no. GCA_004785725.2), *P. grisea* NI907 (GenBank accession no. GCA_004355905.1), and SCAU-2, obtained in this study. Using the single-copy orthologous genes among the four species, the branch-site model in Codeml (Codeml in paml version 4.10.0) was employed to detect genes under positive selection. The parameters were set as follows. For the null hypothesis, model = 2, NSsites = 2, fix-omega = 1, and omega = 1; for the alternative hypothesis, model = 2, NSsites = 2, fix-omega = 0, and omega = 2. A likelihood ratio test (LRT) between the alternative and null hypotheses was performed to calculate *p*-values, followed by the calculation of the false discovery rate (FDR) using the method of Benjamini and Hochberg. A gene was considered to be under positive selection only when the FDR < 0.05. Finally, an enrichment analysis was carried out on the positively selected genes (https://www.omicshare.com/, accessed on 15 October 2023).

## 3. Results

### 3.1. Isolation and Identification of Three Fungal Strains from Leaf Blast in Barnyard Grass

**Figure 1:**
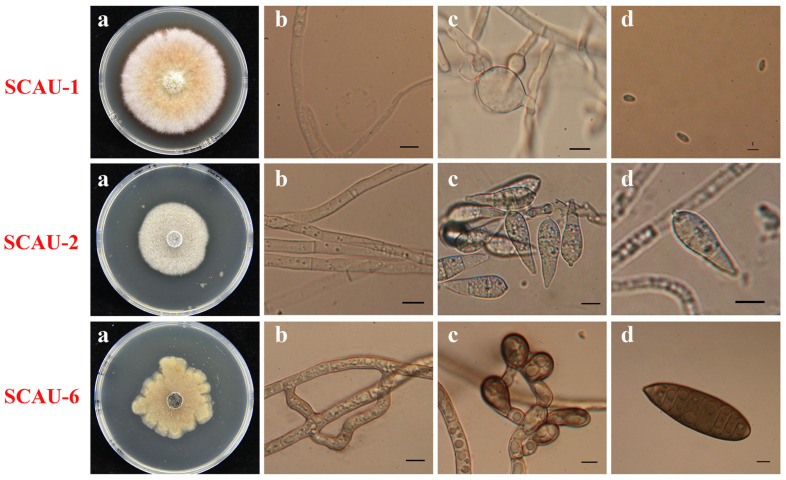
Morphological characteristics of three fungal strains isolated from barnyard grass blast. (**a**) Colony morphology; (**b**) Mycelial morphology; (**c**) Spore-producing structure; (**d**) Conidia morphology. Scale bars = 100 μm. The fungi were grown for 5 days before being photographed.

We isolated three fungal strains from the diseased leaves of barnyard grass growing near the rice field. Molecular identification based on ITS sequences suggested that these three strains, namely SCAU-1, SCAU-2, and SCAU-6, respectively, belong to the *Epicoccum* (SCAU-1), *Pyricularia* (SCUA-2), and *Exserohilum* (SCAU-6) genera (Figure S1). When grown on PDA medium, SCAU-1 formed an orange-red colony with dense mycelium. The conidiospores of SCAU-1 were transparent, single-celled, and oval-shaped, with sporogenous cells that were transparent, smooth, and round (Figure 1). SCAU-2 grew as a greyish-white velvety colony and became melanin-colored at the late growth stage, with pear-shaped conidiospores with two septa (Figure 1). SCAU-6 had a greyish-brown colony with a central protrusion and cottony. The SCAU-6 conidia were dark brown and long and elliptical, with a slightly thicker middle part, possessing multiple septa (Figure 1). The morphologies of mycelia and conidia for each strain fit the genus identification based on their respective ITS sequences.

Given that the SCAU-2 was identified as belonging to the *Pyricularia* genus with typical characteristics of *Pyricularia* mycelial and conidial morphology, we performed PCR amplification using nine pairs of primers (Table S1), as well as a further DNA sequencing analysis to detect the established *Pyricularia Avr* genes. The *P. oryzae* strains B157 and Y34 were included as control strains. Three *Avr* genes, namely *Avr-Pia*, *Avr-Pita2*, and *ACE1*, were detected only in SCAU-2 (Figure S2). The amplified band, using *Avr-Pita2* primers and SCAU-1 as a template (Figure S2, asterisk), turned out to be a sequence not related to the established *Avr-Pita2* gene. Therefore, none of the *Avr* genes were detected in SCAU-1 or SCAU-6. This further confirms that SCAU-2 is a *Pyricularia* strain.

**Figure 2:**
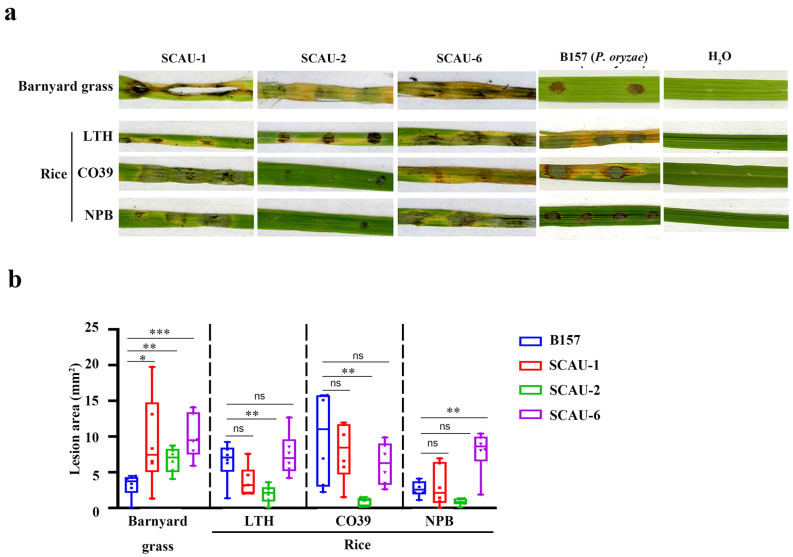
Pathogenicity tests. (**a**) Spore suspensions (10^5^/mL) of SCAU-1, SCAU-2, or SCAU-6 were inoculated on the detached leaves of barnyard grass and different rice varieties (LTH, CO39, and Nipponbare). *P. oryzae* B157 served as a positive control and H_2_O as a blank control. (**b**) Quantitative analysis of lesion area based on a leaf infection assay. The mean ± S.D. was derived from six independent biological repeats. Statistical analysis was performed using a two-tailed unpaired Student’s *t*-test versus B157. ns—no significance; *—*p* < 0.05; **—*p* < 0.01; ***—*p* < 0.001.

A pathogenicity test was performed for these three strains, with the *P. oryzae* strain B157 as a control. The results in Figure 2 show that all three strains can successfully infect barnyard grass. However, SCAU-1 and SCAU-6 exhibited more severe lesion symptoms than SCAU-2 on rice cultivars LTH, CO39, and Nipponbare (NPB) (Figure 2b). Therefore, we confirmed that these three strains were capable of causing blast lesions in barnyard grass and rice leaves to various degrees.

### 3.2. Genome Sequencing and Assembly

Next, we performed whole-genome sequencing for SCAU-1, SCAU-2, and SCAU-6 using Illumina technology. After conducting quality control on the raw data, de novo assembly of the genomes gave rise to 31.36 Mb, 41.20 Mb, and 34.85 Mb, respectively, for SCAU-1, SCAU-2, and SCAU-6, with a total number of 252, 1764, and 439 contigs, respectively. The GC content of the genomes of these three strains was similar, at 52%, 50%, and 50%, respectively (Table 1). According to the BUSCO assessment of the genomes, the number of complete and single-copy genes in the three strains was 238 (93.3%), 249 (97.6%), and 251 (98.4%) (Table 2). Comparative genome average nucleotide identity (ANI) analysis revealed that SCAU-1 had a similarity of 96.36% with the *E. sorghinum* strain USPMTOX48, SCAU-2 had a similarity of 99.68% with *P. grisea* NI907, and SCAU-6 had a similarity of 99.26% with *E. rostratum* BF9006 (Table S2). A total number of 11,242, 11,594, and 11,408 protein-coding genes were predicted in the three genomes, respectively (Table 3).

We successfully retrieved the sequences of *Avr-Pia*, *Avr-Pita2*, and *ACE1* from the SCAU-2 genome, but not from the SCAU-1 or SCAU-6 genomes, verifying the PCR detection of these three *Avr* genes in SCAU-2 only (Figure S2). The coding sequence of the *Avr-Pia* gene in SCAU-2 is 100% consistent with the referenced gene (NCBI accession no. AB498873.1), based on sequence alignment (Figure S3). Compared to the referenced *Avr-Pita2* sequence (NCBI accession no. AB607343.1), we found a C to G mutation and a C to T mutation in the coding sequence of *Avr-Pita2* in SCAU-2 (Figure S4), giving rise to a H to Q mutation at 78 aa and a H to Y mutation at 110 aa in the AVR-Pita2 protein of SCAU-2 (Figure S5). For the DNA sequence of *ACE1* retrieved from the SCAU-2 genome, we found numerous point mutations in the coding sequence region, and two short insertions (23 nt and 24 nt), respectively, in the first and third introns of the referenced *ACE1* sequence (NCBI accession no. AJ704622.1; Figure S6). As SCAU-2 was closely related to the *P. grisea* strain NI907 (Table S2), we inferred that such variants in the DNA sequence of *ACE1* may reflect the difference between *P. oryzae* and *P. grisea* species. To verify this, we compared the *ACE1* sequences from the *P. oryzae* strains Guy11 (the referenced sequence), 70-15, B71, and B2 (among which Guy11 and 70-15 infect rice, while B71 and B2 infect wheat), and the *P. grisea* strains NI907 and W97-11 (both of which infect grass), together with SCAU-2 and the reference sequence (from the *P. oryzae* strain Guy11, which infects rice). The results showed that the *ACE1* sequences were indeed mostly divergent between *P. grisea* and *P. oryzae* species (Figure S6). The two short insertions in the first and third introns were exclusively present in the three grass-infecting strains, and completely conserved in sequence (Figure S6). Mutations in the nucleotide sequence led to changes in the amino acid sequences of ACE1 proteins, which also showed a clear divergence between *P. grisea* and *P. oryzae* strains (Figure S7). Overall, the complete sequences of the three PCR-amplified *Avr* genes could be retrieved from the SCAU-2 genome, and phylogenic tree analysis based on the ACE1 protein supports the fact that SCAU-2 belongs to *P. grisea* (Figure S8), which is consistent with the ANI-based clustering analyses.

Repetitive sequences are ubiquitous in the genomes of eukaryotic organisms, and their proportion is closely related to the genome’s size [61]. In this study, repetitive sequences account for 3.17%, 6.20%, and 5.70% of the genomes of SCAU-1, SCAU-2, and SCAU-6, respectively. Transposable elements are an important component of repetitive sequences, and transposons can promote gene evolution through chromosomal rearrangements and other forms of genomic variation [62]. DNA transposons were predicted in all three strains, at frequencies of 114, 439, and 362, respectively, accounting for less than 1% of the total genome length. Notably, long interspersed nuclear element (LINE) retrotransposons were only present in SCAU-2, while short interspersed nuclear element (SINE) retrotransposons were only identified in SCAU-1. In addition, the 366 LTR retrotransposons identified in SCAU-1 were all of the Gypsy/DIRS1 type, while the 1034 and 874 LTR retrotransposons identified in SCAU-2 and SCAU-6 were mainly Gypsy/DIRS1 and Ty1/Copia types (Table 3). Non-coding RNAs (ncRNAs), as a new class of regulatory factors, play important roles in cellular processes such as growth, differentiation, survival, and apoptosis [63]. In this study, ncRNAs were predicted in the three genomes, including 130, 129, and 86 tRNAs, 52, 35, and 37 rRNAs, and 27, 22, and 27 snRNAs, respectively. A total number of three small RNAs (sRNAs) were predicted in each genome (Table 3).

### 3.3. Prediction of Pathogenicity-Related Genes

The distribution of gene length in the SCAU-1, SCAU-2, and SCUA-6 genomes is shown in Figure S9a, and the majority of gene lengths in the three strains are 800–1200 bp. We performed functional annotation of the three genomes using conventional databases such as GO (Gene Ontology), KEGG (Kyoto Encyclopedia of Genes and Genomes), NR (Non-Redundant protein sequence), and KOG (Eukaryotic Orthologous Groups). As shown in Figure S9b, SCAU-1, SCAU-2, and SCAU-6 had 10,537, 11,073, and 11,065 coding genes annotated in the four databases, respectively. Next, we used the Pfam and Swissprot protein databases for protein function annotation of the coding genes of the three strains. The number of annotated genes in all three strains was more than 50% (Figure S9c).

**Figure 3:**
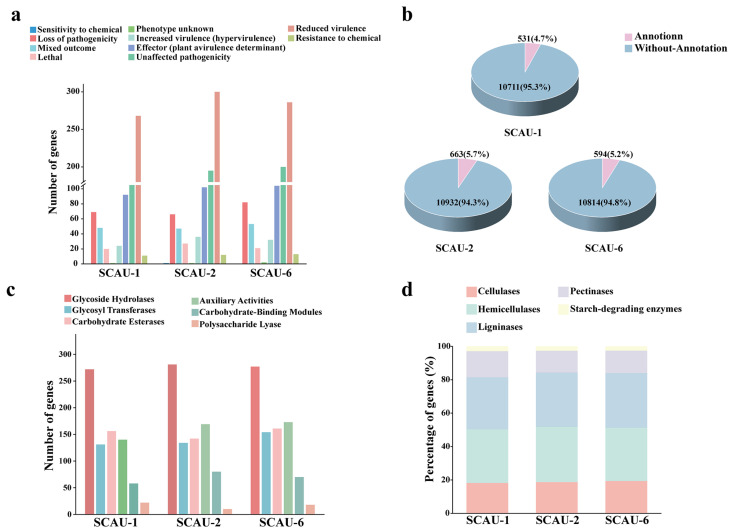
Annotation of genes in SCAU-1, SCAU-2, and SCAU-6 based on the PHI, DFVF, and CAZy databases, together with a comparison of plant cell wall degradation enzymes. (**a**) PHI annotation statistics of the three pathogens. (**b**) Proportion of annotated genes for SCAU-1, SCAU-2, and SCAU-6 in the DFVF database. (**c**) CAZy functional annotation of the three pathogens. (**d**) Comparison of FCWDEs among the three pathogens.

To identify potential virulence genes in the fungal pathogens, we used the Pathogen–Host Interactions (PHI) database and Database of Virulence Factors in Fungal Pathogens (DFVF) for gene annotation. In SCAU-1, SCAU-2, and SCAU-6, a total of 533, 609, and 577 PHI-related genes were annotated. Among these, the number of genes associated with increased pathogenicity (hypervirulence) amounted to 24, 36, and 32, while effector (plant avirulence determinant) genes numbered 92, 102, and 104 (Figure 3a), respectively. Details on PHI-related genes (identity exceeding 90%) can be found in Table S3. In the DFVF database, 531, 663, and 594 coding genes were annotated in SCAU-1, SCAU-2, and SCAU-6, respectively (Figure 3b). Details on DFVF-related genes (identity exceeding 90%) can be found in Table S4. In addition, carbohydrate-active enzymes are also closely related to the growth, development, and pathogenicity of fungi [64,65]. There were 779, 816, and 853 genes in SCAU-1, SCAU-2, and SCAU-6 annotated to the CAZy database, respectively. Among them, glycoside hydrolases (GHs) were the most abundant, with 272, 281, and 277 in each strain. SCAU-1 contained a higher number of carbohydrate esterases (CEs), while SCAU-2 and SCAU-6 had a higher number of auxiliary activities (AAs). The least prevalent were polysaccharide lyases (PLs) (Figure 3c). During the invasion process into their hosts, pathogens secrete a large amount of cell wall-degrading enzymes to destroy the host plant’s cell wall structure. Fungal cell wall-degrading enzymes (FCWDEs) are mainly divided into cellulases, hemicellulases, ligninases, pectinases, and starch-degrading enzymes [66]. In the three strains, we found that hemicellulases and ligninases were relatively higher (more than 60%) in quantity among the total FCWDEs, followed by cellulases and pectinases, and the number of starch-degrading enzymes was the lowest (Figure 3d). Further cluster analysis of plant cell wall-degrading enzymes in SCAU-1, SCAU-2, and SCAU-6 showed that the clustering relationship between SCAU-1 and SCAU-6 was closer, indicating that the plant cell wall-degrading enzymes contained in SCAU-1 and SCAU-6 were more similar in composition (Figure S10a). Classifications of FCWDEs in each strain are shown in Table S5.

**Figure 4:**
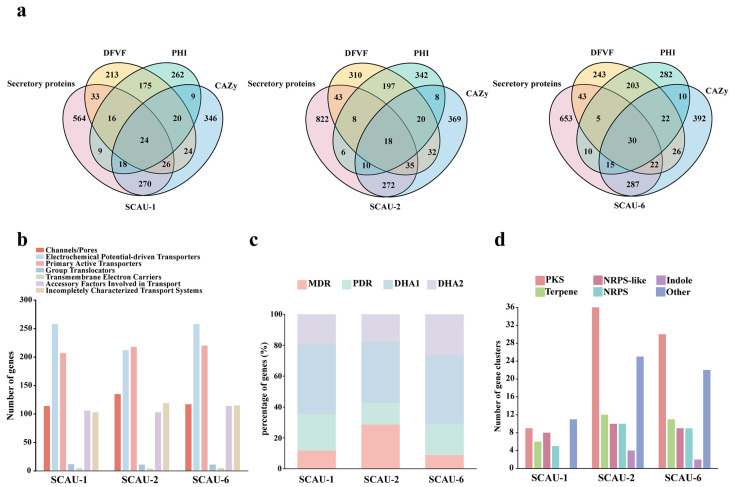
Identification of pathogenic factors, prediction of membrane transport proteins, and enzymes for the biosynthesis of secondary metabolites. (**a**) Venn diagrams showing the number of genes encoding secreted proteins, namely DFVF, PHI, and carbohydrate-active enzymes, in SCAU-1, SCAU-2, and SCAU-6. (**b**) TCDB annotation statistics of the three pathogens. (**c**) Comparison of multidrug transporters in the ABC family and MFS family of the three pathogens. (**d**) Statistics of secondary metabolite synthesis gene clusters in the three pathogens.

A total of 960, 1214, and 1065 proteins were identified as secreted proteins in the three strains. At the same time, we conducted effector protein prediction and identified 360, 556, and 474 effector proteins, respectively (Table 3). SCAU-1 and SCAU-6 contain a higher number of apoplastic effectors (227 and 280, respectively) than cytoplasmic effectors (133 and 194, respectively), while in SCAU-2, the numbers of both types of effectors are similar, with 280 cytoplasmic effectors and 276 apoplastic effectors (Table 3). To further explore the key pathogenic factors of pathogenic fungi, we compared the genes encoding PHI, DFVF, CAZy, and secreted proteins. Among SCAU-1, SCAU-2, and SCAU-6, there were 24, 18, and 30 coding genes, respectively, that have been annotated in four databases (Figure 4a), including some important carbohydrate-active enzymes such as PL3, GH18, CE5, GH7, and GH18 (Table S6). We suggest that these genes may be involved in multiple biological pathways, which could play a crucial role in the pathogenicity of the pathogen and could be further studied.

Membrane transport proteins play a crucial role in the interaction between pathogens and their hosts. In SCAU-1, SCAU-2, and SCAU-6, a total of 805, 802, and 840 membrane transport proteins were predicted, respectively. Among these membrane transport proteins, electrochemical potential-driven transporters make up the largest proportion, with a total of 258, 212, and 258 in SCAU-1, SCAU-2, and SCAU-6, respectively (Figure 4b). All three strains contain more MFS (major facilitator superfamily) transporters (98, 63, and 89, respectively) than ABC (ATP-binding cassette) transporters (39, 44, and 40, respectively) (Table 4). Both ABC and MFS superfamilies contain multidrug transporters, which protect the pathogens from adverse conditions [67,68]. Among these three pathogenic strains, SCAU-2 contains the largest number of multidrug transporter proteins, while the total number of multidrug transporter proteins in SCAU-1 and SCAU-6 are comparable (Figure 4c). In SCAU-1, the most abundant proteins are DHA1 and pleiotropic drug resistance (PDR) proteins, while DHA1 and multidrug resistance (MDR) proteins are the most abundant in SCAU-2. Hydrogen ion translocating proteins (DHA2 and DHA1) account for the greatest proportion in SCAU-6 (Figure 4c).

Plant pathogens can produce a large number of secondary metabolites that act as virulence factors, aiding their infection of the hosts [69]. In the genomes of SCAU-1, SCAU-2, and SCAU-6, we identified 39, 97, and 83 secondary metabolite biosynthetic gene clusters, respectively. PKS was the most abundant among the secondary metabolite biosynthetic gene clusters in all three strains. In SCAU-2 and SCAU-6, the number of NRPS and NRPS-like gene clusters were comparable. In the SCAU-1, SCAU-2, and SCAU-6 genomes, the number of gene clusters encoding indole is the lowest (Figure 4d). Based on the secondary metabolism core genes in SCAU-2, we searched for common genes among the three tested strains. As shown in Figure S10b, there are 17 core genes of secondary metabolism shared by SCAU-1, SCAU-2, and SCAU-6, including three terpene genes, three NRPS genes, six PKS genes, and five NRPS-like genes. In addition, we also predicted the genes encoding cytochrome P450 in the three strains. The results indicated that the number of predicted cytochrome P450 genes in SCAU-1, SCAU-2, and SCAU-6 was less than 5% of the number of coding genes (Figure S9c).

### 3.4. Comparative and Phylogenetic Analyses

**Figure 5:**
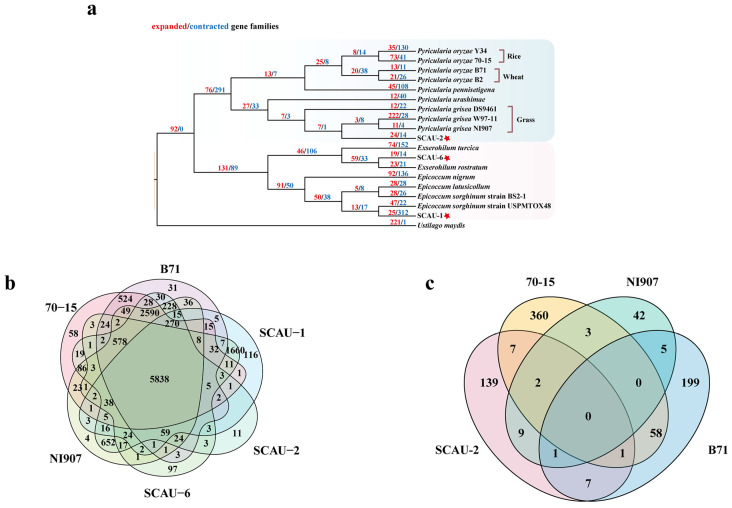
Phylogenetic and selection pressure analysis. (**a**) Constructing a phylogenetic tree based on single-copy orthologous gene sequences using the Maximum Likelihood (ML) method. Red numbers represent the number of expanded gene families and blue numbers represent the number of contracted gene families. The red pentagram represents the strains isolated in this study. (**b**) Clustering analysis of gene families from six pathogenic fungi. (**c**) Venn diagrams showing positive selection genes in *Pyricularia* strains SCAU-2, 70-15, NI907, and B71.

To investigate the phylogenetic relationships between the three fungal isolates and the fungi belonging to the same genus, we constructed a phylogenetic tree using the maximum likelihood (ML) method. As shown in Figure 5a, SCAU-1 was clustered with the *E. sorghinum* strain USPMTOX48 into one clade, SCAU-6 was clustered with a strain belonging to *E. rostratum*, and SCAU-2 was clustered with the grass-infecting *P. grisea* strains NI907, W97-11, and DS9461. Compared to SCAU-2, SCAU-1 and SCAU-6 have a closer phylogenetic relationship (Figure 5a).

During the evolution of pathogenic fungi, frequent gene loss or gain is a mechanism for species adaptation to hosts, which is largely driven by selective pressure from the host plants [70,71,72]. In SCAU-1, a total of 25 gene families were expanded and 312 gene families were contracted. However, compared to SCAU-1, SCAU-2 and SCAU-6 have fewer expanded (24 and 19, respectively) and contracted (14 and 14, respectively) gene families (Figure 5a). Further study of the expanded gene families showed that significantly (*p* < 0.01) expanded genes in SCAU-1 can encode the glycoside hydrolase family GH47, the carbohydrate esterase family CE1, auxiliary activities (AA3 and AA8), and PDR in ABC class transporters. In SCAU-2, the significantly (*p* < 0.01) expanded genes are mainly associated with the carbohydrate esterase family (CE4, CE7, and CE10). In SCAU-6, the significantly (*p* < 0.01) expanded genes are related to the functions of glycoside hydrolases (GH16 and GH109), carbohydrate esterases (CE10), auxiliary activities (AA8), and DHA1 in MFS class transporters (Table S7).

Next, we analyzed the shared and unique gene families in SCAU-1, SCAU-2, and SCAU-6 compared with *P. oryzae* 70-15 (rice-infecting), *P. oryzae* B71 (wheat-infecting), and *P. grisea* NI907 (grass-infecting). Using OrthoFinder Version 2.5.5 software, we conducted a comparative genomic study on these six strains and found that there were 5838 shared gene families among them. SCAU-1 and SCAU-6 had 116 and 97 unique gene families, respectively, while SCAU-2 had only 11 unique gene families, and most of the unique genes in these three genomes are unannotated. Notably, among the unique genes in SCAU-2, nine genes encode effector proteins, and three of them did not have corresponding matches in the existing databases. Additionally, SCAU-2 shares 652 common gene families with NI907 and only has 30 and 19 common gene families, respectively, with B71 and 70-15 (Figure 5b), which reflects the fact that SCAU-2 is indeed closer to the grass-infecting *P. grisea* strains than those infecting other hosts.

To understand the effect of selective pressures on the genome of SCAU-2 during its evolutionary process, we employed the branch-site model to analyze positive selection on orthologous single-copy genes in the *P*. *oryzae* strains 70-15 and B71 and the *P. grisea* strains NI907 and SCAU-2. As shown in Figure 5c, a total of 431 positively selected genes were identified in the 70-15 branch, 271 in the B71 branch, 166 in the SCAU-2 branch, and 62 in the NI907 branch. No genes were detected under positive selection across all four branches. We identified 139, 360, 42, and 199 unique positively selected genes in SCAU-2, 70-15, NI907, and B71, respectively (Figure 5c). We found that in the B71 vs. SCAU-2 branches and the 70-15 vs. SCAU-2 branches, a total of seven genes were positively selected together. A total of nine genes were commonly under positive selection between the NI907 vs. SCAU-2 branches (Figure 5c). According to NR annotations, most of the positively selected genes are hypothetical proteins (Table S8). Specifically, on the SCAU-2 vs. 70-15 branches, positively selected genes encode a terpene synthase metal-binding domain-containing protein, D-alanine-poly (phosphoribitol) ligase subunit 1, and an inorganic phosphate transporter, PHO87. On the B71 vs. SCAU-2 branches, positively selected genes encode a CCR4-Not complex subunit and aspartyl-tRNA synthetase. Genes encoding a protein kinase domain-containing protein and cysteine desulfurase were under positive selection on the NI907 vs. SCAU-2 branches (Table S8). Additionally, 58 genes were found to be under positive selection in both 70-15 and B71 lineages (Figure 5c), which is noticeably more than the number of genes under positive selection common to other strains. These results suggest that SCAU-2 may face the same or similar selective pressures as these three *Pyricularia* strains during its evolutionary process.

In SCAU-2, we identified 139 unique positively selected genes, based on which we conducted an enrichment analysis. In terms of cellular components (CCs), the genes were mainly enriched in the 3-isopropylmalate dehydratase complex, myosin filaments, the microtubule organizing center attachment site, etc. (Table S9). In molecular function (MF), the enriched terms include transcriptional repressor activity, transcription factor activity, *N*,*N*-dimethylaniline monooxygenase activity, etc. (Table S9). The terms enriched in biological processes (BPs) include toxin metabolic processes, as well as secondary metabolic processes, organic heteropentacyclic compound metabolic processes, regulation of hormone levels, etc. (Table S9). Through KEGG enrichment analysis, the positively selected genes in SCAU-2 were mainly enriched in pathways related to pyruvate metabolism, microbial metabolism in diverse environments, naphthalene degradation, glycolysis/gluconeogenesis, carbon metabolism, the degradation of aromatic compounds, etc. (Table S10).

## 4. Discussion

In this study, we isolated a *P. grisea* strain, SCAU-2, from the leaf blast of barnyard grass, which displayed weaker pathogenicity in the tested rice cultivars (Figure 2), indicating the infectivity preference of this isolate. We also isolated two other strains belonging to *E. sorghinum* and *E. rostratum*, respectively, which were able to infect the detached leaves of rice cultivars and barnyard grass (Figure 2). Therefore, the presence of *Epicoccum* or *Exserohilum* spp. in barnyard grass growing near rice farms could pose a risk for the emergence of new disease(s) in rice. In addition, these fungal strains could be used as a resource for investigations into the evolutionary mechanism of host selection/expansion, based on comparative genomics.

For deciphering pathogen–host specificity, we determined the presence or absence of a few representative *Avr* genes in three pathogens and compared their site variations with reference sequences (Table S1). *Avr-Pia*, *Avr-Pita2*, and *ACE1* were only present in SCAU-2 and not in SCAU-1 or SCAU-6 (Figure S2). We are particularly intrigued to notice that the ACE1 protein of SCAU-2 was conserved among *P. grisea* strains and separated from the cluster of *P. oryzae* strains (Figure S8). *ACE1* is a special *Avr* gene as it encodes a hybrid polyketide synthase/nonribosomal peptide synthetase (PKS-NRPS) belonging to a cluster of secondary metabolism genes specifically expressed at an early stage of infection [73]. *P. oryzae* isolates carrying the functional *ACE1* gene are unable to infect rice cultivars carrying the corresponding *R* gene *Pi33* [74]. In this study, we identified a variant of *ACE1* from a barnyard grass-infecting strain, SCAU-2, belonging to *P. grisea*. Further verification is required to see whether the ACE1 protein from *P. grisea* is functional and whether it contributes to the determination of host specificity. In addition, our results also indicate that the *ACE1* gene could be used as an improved marker gene (compared to the ITS fragment) for determining *P. oryzae* and *P. grisea* species.

Apart from detecting and comparing the *Avr* genes, we further conducted whole-genome sequencing of these three strains. Transposable elements may be related to the virulence variation and genomic plasticity of fungal strains [75]. We found that all three fungal strains contain a large number of retrotransposons (LINE, SINE, and LTR) and fewer DNA transposons. Our study also found that LINE only exists in SCAU-2 and SINE was only found in SCAU-1 (Table 3). Consistent with previous studies [76], LINEs and SINEs do not exist in SCAU-6. To determine the pathogenic genes in the three fungal pathogens, we annotated the genes in the three fungal pathogens with databases such as PHI, DFVF, and CAZy (Figure 3). Based on the annotation results from the PHI database, *MAK2* [77], which is highly similar to the established pathogenicity factor *PMK1* in *P. oryzae*, was identified in all three isolates (Table S3). This suggests that SCAU-1 and SCAU-6 have the potential to induce rice diseases through a mechanism similar to that of *P. oryzae*. Cell wall-degrading enzymes are very important and enable pathogens to successfully invade the host [78]. We found that the number and type of cell wall-degrading enzymes were more abundant in SCAU-6 and SCAU-1 than in SCAU-2 (Table S5), which was also the case for multidrug transporters. This can at least partially explain why SCAU-2 exhibits reduced virulence on rice leaves compared to SCAU-1 and SCUA-6.

In the effector protein analysis, we found that SCAU-1 and SCAU-6 contained more apoplastic effectors than cytoplasmic effectors, while SCAU-2 had a similar number of cytoplasmic and apoplastic effectors (Table 3). Cytoplasmic effectors migrate to plant cells through the biotrophic interface complex (BIC), while apoplastic effectors are dispersed in the extracellular space between the fungal cell wall and the non-invasive extra-invasive hyphal membrane (EIHM) and function on the outside of plant cells [79]. In our study, the differences in the types and numbers of effectors of the three isolates indicated that they adopted different infection strategies when infecting host plants. In recent years, studies have shown that effector proteins can act as host-specific factors to mediate the host specificity of pathogenic fungi and evolve under host selection pressure to avoid being recognized by the immune system of host plants [80,81]. After annotating the effector proteins of the three strains, we found that most of these effector proteins were classified as hypothetical proteins, with only a small portion belonging to carbohydrate-active enzymes. For example, in SCAU-2, 5701_t and 11239_t exhibited high sequence identity with pectate lyase (NCBI accession no. XP_003711703.1, Identity%: 97%) and glucan 1,3-beta-glucosidase (NCBI accession no. XP_003710843.1, Identity%: 92%) from *P. oryzae*, respectively. Similarly, in SCAU-6, 10684_t exhibited extremely high sequence identity with the carbohydrate esterase family 1 protein from *Bipolaris oryzae* (NCBI accession no. XP_007693070.1, Identity%: 94%). Expansion analysis of gene families indicated that there are six and three genes encoding effector proteins expanded in SCAU-1 and SCAU-6, respectively (Figure 5a). Although the specific functions of these effector proteins are not yet clear, this result may imply that the pathogen has undergone adaptive evolution in the late stages of evolution, enabling it to infect and adapt to a wider range of hosts. Additionally, through the analysis of the 11 unique gene families in SCAU-2, we discovered that there are nine genes encoding effector proteins (Figure 5b), and it is speculated that the presence of these effector proteins might be used to overcome the basal immunity of the host plant.

To decipher the natural selection pressures that SCAU-2 experienced during its evolutionary process, we performed enrichment analysis on the unique genes under positive selection in SCAU-2 and found that these genes are involved in various metabolic pathways (Tables S9 and S10). Among them, the *LEU1* gene, which is involved in the biosynthetic pathway of leucine, has been reported to regulate the growth, pathogenicity, and virulence of *Fusarium graminearum* [82] and *P. oryzae* [83] (Table S9). *N*,*N*-dimethylaniline monooxygenase activity is involved in drug metabolism and detoxification reactions [84]. We also discovered that SCAU-2 is enriched in naphthalene degradation, aromatic compound degradation, toxin metabolism processes, and toxin biosynthesis processes (Tables S9 and S10). Naphthalene is a type of low-molecular-weight polycyclic aromatic hydrocarbon (PAH) that is widely used and has caused serious environmental pollution and cytotoxic and genotoxic effects on living organisms [85]. The enrichment of naphthalene degradation genes in the SCAU-2 genome may reflect the long-term exposure of rice and/or barnyard grass to naphthalene and other aromatic compounds, which exerted selective pressure on the microbes living in this environment. In addition, it suggests that SCAU-2 can be used as a biocontrol agent for soil contaminated with naphthalene.

## 5. Conclusions

Overall, we isolated three fungal strains from barnyard grass and assessed their pathogenicity (host specificity). We further used comparative genomics to explore potential disease-causing genes, as well as the evolutionary relationship between the strains of different species infecting the same host, or among strains of the same species but with different host specificities. Our results reveal that the genome of the *P. grisea* strain SCAU-2, originating from barnyard grass, may have undergone adaptive evolution, influencing the pathogenicity of the strains to rice, and we provide genomic information for *E. sorghinum* and *E. rostratum,* which cause rice diseases. This study provides valuable data support for the future research of pathogenic fungi and is helpful for formulating the prevention and control strategy of rice diseases. Barnyard grass in rice fields should be eliminated as the pathogens causing diseases in barnyard grass may have the potential to infect rice as SCAU-1, SCAU-2, and SCAU-6 do.

## Figures and Tables

**Table 1:** Genome assembly statistics.

Features	SCAU-1	SCAU-2	SCAU-6
All Length (bp)	31,364,396	41,199,273	34,845,801
Contig num	252	1764	439
Contig max len (bp)	1,632,077	300,235	581,263
Contig average len (bp)	124,461.89	23,355.60	79,375.40
Contig N50 (bp)	237,036	59,266	184,926
GC Ratio	52%	50%	50%

**Table 2:** Genome assembly evaluation.

Features	SCAU-1	SCAU-2	SCAU-6
Complete and single-copy	238 (93.3%)	249 (97.6%)	251 (98.4%)
Complete and duplicated	0 (0.0%)	0 (0.0%)	1 (0.4%)
Fragmented	3 (1.2%)	5 (2.0%)	2 (0.8%)
Missing (not recovered in assembly)	14 (5.5%)	1 (0.4%)	1 (0.4%)
Total BUSCO	255 (100%)	255 (100%)	255 (100%)

**Table 3:** Genome component analysis.

Prediction Features	SCAU-1	SCAU-2	SCAU-6
Gene Number	11,242	11,594	11,408
tRNA	130	129	86
rRNA	52	35	37
sRNA	3	3	3
snRNA	27	22	27
DNA transposons	114	439	362
LINE	-	169	-
SINE	34	-	-
LTR	366	1034	874
Gypsy/DIRS1	366	891	596
Ty1/Copia	-	139	278
Secreted Protein Number	960	1214	1065
Effect Protein Number	360	556	474
Cytoplasmic effector	133	280	194
Apoplastic effector	227	276	280

“-” stands for non-existence.

**Table 4:** The number of ABC family and MFS family transporters.

Features	SCAU-1	SCAU-2	SCAU-6
ABC	39	44	40
MFS	98	63	89
Other	669	696	714
Total	806	803	843

## Data Availability

The whole-genome sequencing data and genome assemblies were deposited in the SRA (SRX24268339 for SCAU-1, SRX24268934 for SCAU-2, and SRX24269188 for SCAU-6) and assembly (GCA_040113005.1 for SCAU-1, GCA_040113025.1 for SCAU-2, and GCA_040113035.1 for SCAU-6) at NCBI BioProject PRJNA1100412 (SCAU-1), PRJNA1100628 (SCAU-2), and PRJNA1100706 (SCAU-6). All the experimental data related to this work were included in the manuscript, figures, or Supplementary Information/dataset(s).

## Supplementary Materials

The following supporting information can be downloaded at: https://www.mdpi.com/article/10.3390/jof10120868/s1, Figure S1: The phylogenetic tree was constructed based on ITS sequences; Figure S2: Agarose gel electrophoresis image of amplified *Avr* genes from isolated strains; Figure S3: DNA sequence alignment of *Avr-Pia*; Figure S4: DNA sequence alignment of *Avr-Pita2*; Figure S5: Amino acid sequence alignment of AVR-Pita2; Figure S6: DNA sequence alignment of *ACE1*; Figure S7: Amino acid sequence alignment of ACE1; Figure S8: The phylogenic tree analysis of ACE1; Figure S9: Distribution of gene lengths and annotations in general databases for the isolated strains; Figure S10: Cluster analysis of plant cell wall-degrading enzymes (FCWDEs) and the presence/absence of core genes for secondary metabolite biosynthesis in the three pathogens; Table S1: Primers used in this study; Table S2: Average nucleotide identity (ANI) analysis; Table S3: The coding genes of the three tested strains with a sequence identity greater than 90% to those in the PHI database; Table S4: Comparison of the predicted virulence genes in the three tested strains (identity > 90%); Table S5: Distribution of cell wall degradation enzymes in the three fungal strains; Table S6: Carbohydrate-active enzymes encoded by common genes in 4 databases; Table S7: Annotated results of isolated strain-expanding genes (*p* < 0.01) in the CAZy and TCBD databases; Table S8: Positive selection genes of SCAU-2 and different *Pyricularia* strains; Table S9: GO enrichment analysis of genes under positive selection in SCAU-2; Table S10: KEGG enrichment analysis of genes under positive selection in SCAU-2.
