# Supplementary material for: Comparative Genomics and Pathogenicity Analysis of Three Fungal Isolates Causing Barnyard Grass Blast
Source: J Fungi (Basel). 2024 Dec 13;10(12):868. doi: 10.3390/jof10120868 (PMC11678098; doi:10.3390/jof10120868)
Supplement: Supplementary file 1 [file jof-10-00868-s001.zip › Fig S1-S10.pdf]

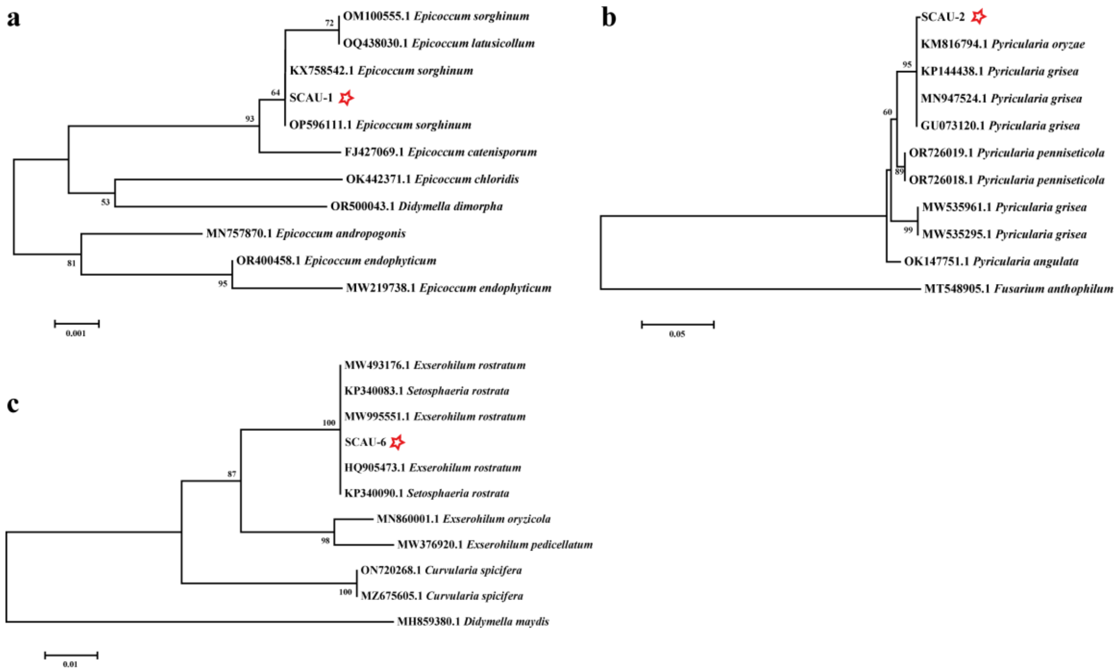

Figure S1. The phylogenetic tree was constructed based on ITS sequence. Based on the ITS sequences, the phylogenetic trees of SCAU-1 (**a**), SCAU-2 (**b**), and SCAU-6 (**c**) were constructed using the neighbor-joining method of MEGA 7 software [51]. The numbers in the figure represent the reliability of each branch structure, with higher values indicating greater reliability, and the red stars represent the species studied in this research.

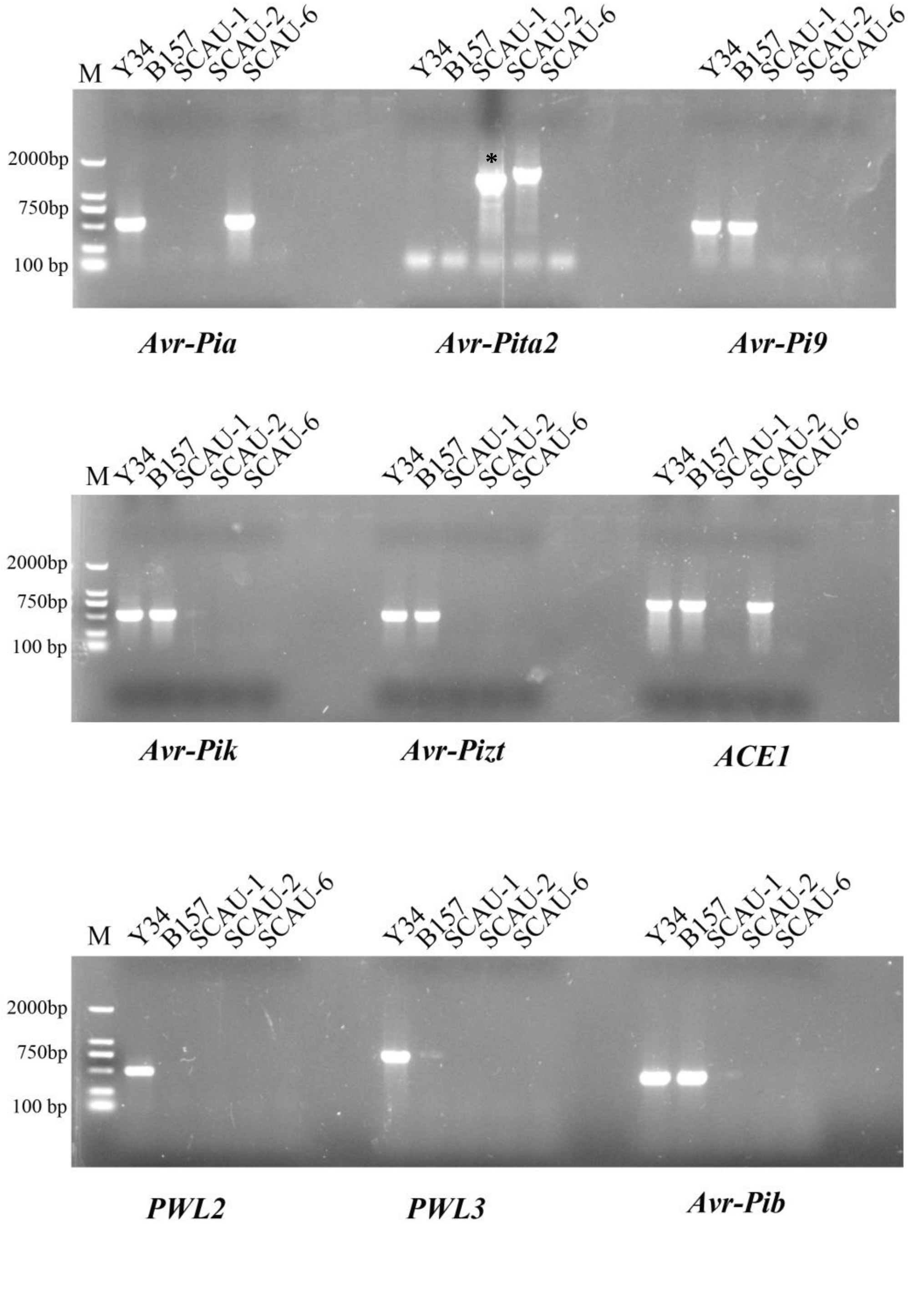

Figure S2. Agarose gel electrophoresis image of amplified *Avr* genes from isolated strains. *Pyricularia oryzae* strains Y34 and B157 served as controls. M: DNA marker DL2000. Asterisk denotes non-specifically amplified band.

```
Ref    : ATGCATTTTTCGACAATTTTCATCCCCTTTGCCTTAGCTGCT :  42
SCAU-2 : ATGCATTTTTCGACAATTTTCATCCCCTTTGCCTTAGCTGCT :  42

Ref    : CTAAAAGTAAGCGCTGCGCCAGCTAGATTTTGCGTCTATTAC :  84
SCAU-2 : CTAAAAGTAAGCGCTGCGCCAGCTAGATTTTGCGTCTATTAC :  84

Ref    : GACGGCCACCTTCCCGCGACACGTGTCCTGCTTATGTACGTT : 126
SCAU-2 : GACGGCCACCTTCCCGCGACACGTGTCCTGCTTATGTACGTT : 126

Ref    : AGAATCGGCACTACAGCGACTATTACGGCCCGTGGGCACGAA : 168
SCAU-2 : AGAATCGGCACTACAGCGACTATTACGGCCCGTGGGCACGAA : 168

Ref    : TTCGAAGTTGAAGCAAAAGACCAGAATTGCAAAGTTATTCTC : 210
SCAU-2 : TTCGAAGTTGAAGCAAAAGACCAGAATTGCAAAGTTATTCTC : 210

Ref    : ACCAATGGCAAACAAGCACCGGATTGGCTTGCTGCCGAGCCT : 252
SCAU-2 : ACCAATGGCAAACAAGCACCGGATTGGCTTGCTGCCGAGCCT : 252

Ref    : TACTAG : 258
SCAU-2 : TACTAG : 258
```

Figure S3. DNA sequence alignment of *Avr-Pia*. The sequence of referenced gene is accessible in NCBI under no. AB498873.1. Sequence alignment was performed by Clustal X2 [49] and shaded by GeneDoc software [50]. The boxes with white character and black background represent that the conserved percentage is 100% in the column.

```
Ref    : ATGCTTTTTTATTCATTTATATTTTATTTTCACACCGTTGCA :  42
SCAU-2 : ATGCTTTTTTATTCATTTATATTTTATTTTCACACCGTTGCA :  42

Ref    : ATTTCGGCCTTCACCAACATTGGCACCTTTTCATACCCAGTT :  84
SCAU-2 : ATTTCGGCCTTCACCAACATTGGCACCTTTTCATACCCAGTT :  84

Ref    : TACAATTCCAATCCAATTCCAAACCATATCCACGGAGATTTA : 126
SCAU-2 : TACAATTCCAATCCAATTCCAAACCATATCCACGGAGATTTA : 126

Ref    : AAAAGGCGGGCTTATATTGAACCCTATTCCCAATGTTCAAAT : 168
SCAU-2 : AAAAGGCGGGCTTATATTGAACCCTATTCCCAATGTTCAAAT : 168

Ref    : TCGCAGGACTCCGAAATTCGTGCCGCGCTAAAAAGTTGTGCC : 210
SCAU-2 : TCGCAGGACTCCGAAATTCGTGCCGCGCTAAAAAGTTGTGCC : 210

Ref    : GAACTCGCCTCGTGGGCCTATCACGCCGTTGAAAATGACAAT : 252
SCAU-2 : GAACTCGCCTCGTGGGCCTATCAGGCCGTTGAAAATGACAAT : 252

Ref    : CGGTTATTTGAATTGATTTTTAAAACTGACAGCACAAATATT : 294
SCAU-2 : CGGTTATTTGAATTGATTTTTAAAACTGACAGCACAAATATT : 294

Ref    : AAAAACTGGGTTCAAAATAATTTTAACGAAATTCACAAGGAA : 336
SCAU-2 : AAAAACTGGGTTCAAAATAATTTTAACGAAATTTACAAGGAA : 336

Ref    : TGTAACAGGGACGCGGACGAAATTTCTCTATCCTGCCACGAT : 378
SCAU-2 : TGTAACAGGGACGCGGACGAAATTTCTCTATCCTGCCACGAT : 378

Ref    : ACAAGTGTTTATACGTGCGTCCGAGAAGGAGTTCATCTTTTG : 420
SCAU-2 : ACAAGTGTTTATACGTGCGTCCGAGAAGGAGTTCATCTTTTG : 420
```

```
Ref    : GGCTATGCAAAGATGTACGAAAAACAAGTTGTTTTATGCCCT : 462
SCAU-2 : GGCTATGCAAAGATGTACGAAAAACAAGTTGTTTTATGCCCT : 462

Ref    : CATTTCTTTGATCACCCCGTAAACAGCAGGGAAATCACTGCC : 504
SCAU-2 : CATTTCTTTGATCACCCCGTAAACAGCAGGGAAATCACTGCC : 504

Ref    : CAAAACCAAGATACAGTTATATTGCATGAAATGCTGCATATA : 546
SCAU-2 : CAAAACCAAGATACAGTTATATTGCATGAAATGCTGCATATA : 546

Ref    : ATTCTAAATGAGTGGGAAGATTATGGTTACGAATGGGATGGG : 588
SCAU-2 : ATTCTAAATGAGTGGGAAGATTATGGTTACGAATGGGATGGG : 588

Ref    : ATTCACAATTTGGATAGTACAACAAGTATTAAAAACCCCGAC : 630
SCAU-2 : ATTCACAATTTGGATAGTACAACAAGTATTAAAAACCCCGAC : 630

Ref    : AGCTATGCTATTTTTGCACAATGTGCACGTTATAAATATTGT : 672
SCAU-2 : AGCTATGCTATTTTTGCACAATGTGCACGTTATAAATATTGT : 672

Ref    : TAA : 675
SCAU-2 : TAA : 675
```

Figure S4. DNA sequence alignment of *Avr-Pita2*. The sequence of referenced gene is accessible in NCBI under no. AB607343.1. Sequence alignment was performed by Clustal X2 [49] and shaded by GeneDoc software [50]. The boxes with white character and black background represent that the conserved percentage is 100% in the column. The boxes with grey background (Ref) or white background (SCAU-2) represent the difference of *Avr-Pita2* gene in referenced strain and SCAU-2.

```
Ref     : MLFYSFIFYFHTVAISAFTNIGTFSYPVYNSNPIPNHIHGDL :  42
SCAU-2  : MLFYSFIFYFHTVAISAFTNIGTFSYPVYNSNPIPNHIHGDL :  42

Ref     : KRRAYIEPYSQCSNSQDSEIRAALKSCAELASWAYHAVENDN :  84
SCAU-2  : KRRAYIEPYSQCSNSQDSEIRAALKSCAELASWAYQAVENDN :  84

Ref     : RLFELIFKTDSTNIKNWVQNNFNEIHKECNRDADEISLSCHD : 126
SCAU-2  : RLFELIFKTDSTNIKNWVQNNFNEIYKECNRDADEISLSCHD : 126

Ref     : TSVYTCVREGVHLLGYAKMYEKQVVLCPHFFDHPVNSREITA : 168
SCAU-2  : TSVYTCVREGVHLLGYAKMYEKQVVLCPHFFDHPVNSREITA : 168

Ref     : QNQDTVILHEMLHIILNEWEDYGYEWDGIHNLDSTTSIKNPD : 210
SCAU-2  : QNQDTVILHEMLHIILNEWEDYGYEWDGIHNLDSTTSIKNPD : 210

Ref     : SYAIFAQCARYKYC : 224
SCAU-2  : SYAIFAQCARYKYC : 224
```

Figure S5. Amino acid sequence alignment of AVR-Pita2. The sequence of referenced protein (Ref) is accessible in NCBI under no. BAK40880.1. Sequence alignment was performed by Clustal X2 [49] and shaded by GeneDoc software [50]. The boxes with white character and black background represent that the conserved percentage is 100% in the column. The boxes with grey background (Ref) or white background (SCAU-2) represent the difference of AVR-Pita2 protein in referenced strain and SCAU-2.

```
SCAU-2 : ATGGGAGACGACATGTGGACCACTAACACTGAGCCTATTGCTATCATCGGCAGTGGCTGTAGATTTCCCGGCGGCTCCACTACACCATCCAAG :  93
NI907  : ATGGGAGACGACATGTGGACCACTAACACTGAGCCTATTGCTATCATCGGCAGTGGCTGTAGATTTCCCGGCGGCTCCACTACACCATCCAAG :  93
W97-11 : ATGGGAGACGACATGTGGACCACTAACACTGAGCCTATTGCTATCATCGGCAGTGGCTGTAGATTTCCCGGCGGCTCCACTACACCATCCAAG :  93
B71    : ATGCGTGACGAGATGTGGAATACTGCCACTGAGCCTATTGCCATCATCGGCAGTGGCTGCAAATTTCCTGGTGGCTCAACGACACCGTCCAAG :  93
B2     : ATGCGTGACGAGATGTGGAATACTGCCACTGAGCCTATTGCCATCATCGGCAGTGGCTGCAAGTTTCCCGGTGGCTCAACGACACCGTCCAAG :  93
Ref    : ATGCGTGACGAGATGTGGAATACTGCCACTGAGCCTATTGCCATCATCGGCAGTGGCTGCAAGTTTCCCGGTGGCTCAACGACACCGTCCAAG :  93
70-15  : ATGCGTGACGAGATGTGGAATACTGCCACTGAGCCTATTGCCATCATCGGCAGTGGCTGCAAGTTTCCCGGTGGCTCAACGACACCGTCCAAG :  93

SCAU-2 : CTCTGGGAACTTTTGAAAGATCCCAAAGACATCGTCAGTGAAATCAAACCTGATCGTTTCGACGTTGACAAATATTTTCATCCAGATCACAAA : 186
NI907  : CTCTGGGAACTTTTGAAAGATCCCAAAGACATCGTCAGTGAAATCAAACCTGATCGTTTCGACGTTGACAAATATTTTCATCCAGATCACAAA : 186
W97-11 : CTCTGGGAACTTTTGAAAGATCCCAAAGACATCGTCAGTGAAATCAAACCTGATCGTTTCGACGTTGACAAATATTTTCATCCAGATCACAAA : 186
B71    : CTCTGGGAGCTTTTGAAAGACCCCAAAGATATCGTCAGTGAAATTAGACCTGACCGATTTGACGTTGACAAATACTTTCATCCGGATCATAAG : 186
B2     : CTCTGGGAGCTTTTGAAAGACCCCAAAGATATCGTCAGTGAAATTAGACCTGACCGATTCGACGTTGACAAATACTTTCATCCGGATCATAAA : 186
Ref    : CTCTGGGAGCTTTTGAAAGACCCCAAAGATATCGTCAGTGAAATTAGACCTGACCGATTCGACGTTGACAAATACTTTCATCCGGATCATAAA : 186
70-15  : CTCTGGGAGCTTTTGAAAGACCCCAAAGATATCGTCAGTGAAATTAGACCTGACCGATTCGACGTTGACAAATACTTTCATCCGGATCATAAA : 186

SCAU-2 : CATCACGGAACCAGCAATGTTCGTCACTCATATTTTTTGGACGAAAACTTCAAGCTTTTCGATGCCAAATTCTTCGGAATTAGGCCCCAAGAA : 279
NI907  : CATCACGGAACCAGCAATGTTCGTCACTCATATTTTTTGGACGAAAACTTCAAGCTTTTCGATGCCAAATTCTTCGGAATTAGGCCCCAAGAA : 279
W97-11 : CATCACGGAACCAGCAATGTTCGTCACTCATATTTTTTGGACGAAAACTTCAAGCTTTTCGATGCCAAATTCTTCGGAATTAGGCCCCAAGAA : 279
B71    : CACCACGGAACCAGCAATGTTCGCCACTCGTATTTTCTCGAAGAAAACTTTAAGCATTTCGATGCCAAATTTTTCGGAATCAGGCCCCAGGAA : 279
B2     : CACCACGGAACCAGCAATGTTCGCCACTCGTATTTTCTGGAAGAAAACTTTAAGCATTTCGATGCCAAATTTTTCGGAATCAGGCCCCAGGAA : 279
Ref    : CACCACGGAACCAGCAATGTTCGCCACTCGTATTTTCTGGAAGAAAACTTTAAGCATTTCGATGCCAAATTTTTCGGAATCAGGCCCCAGGAA : 279
70-15  : CACCACGGAACCAGCAATGTTCGCCACTCGTATTTTCTGGAAGAAAACTTTAAGCATTTCGATGCCAAATTTTTCGGAATCAGGCCCCAGGAA : 279

SCAU-2 : GCTATGGCTATGGATCCCCAGCAACGTTTTCTGTTAGAAACGGTGTATGAAAGCTTGGAGGCGGCTGGGATCACTATCGGTGGTCTCAAAGGC : 372
NI907  : GCTATGGCTATGGATCCCCAGCAACGTTTTCTGTTAGAAACGGTGTATGAAAGCTTGGAGGCGGCTGGGATCACTATCGGTGGTCTCAAAGGC : 372
W97-11 : GCTATGGCTATGGATCCCCAGCAACGTTTTCTGTTAGAAACGGTGTATGAAAGCTTGGAGGCGGCTGGGATCACTATCGGTGGTCTCAAAGGC : 372
B71    : GCCATGGCCATGGACCCCCAGCAACGGTTTCTGTTGGAAACGGTATACGAAAGCCTGGAGGCGGCTGGAATTACCATTAGCGACCTCAAAGGG : 372
B2     : GCCATGGCCATGGACCCCCAGCAACGGTTTCTGTTGGAAACGGTATACGAAAGCCTGGAAGCGGCTGGAATCACCATTAGCGACCTCAAAGGG : 372
Ref    : GCCATGGCCATGGACCCCCAGCAACGGTTTCTGTTGGAAACGGTATACGAAAGCCTGGAAGCGGCTGGAATCACCATTAGCGACCTCAAAGGG : 372
70-15  : GCCATGGCCATGGACCCCCAGCAACGGTTTCTGTTGGAAACGGTATACGAAAGCCTGGAAGCGGCTGGAATCACCATTAGCGACCTCAAAGGG : 372
```

```
SCAU-2 : TCTCAAACCGGAGTTTTTGTCGGAAATATGGGTGTGGACTATTCGGAGCTTCTGTCACAGGATATAGATGCTTTCCCTACCTACTTTGCACCT :  465
NI907  : TCTCAAACCGGAGTTTTTGTCGGAAATATGGGTGTGGACTATTCGGAGCTTCTGTCACAGGATATAGATGCTTTCCCTACCTACTTTGCACCT :  465
W97-11 : TCTCAAACCGGAGTTTTTGTCGGAAATATGGGTGTGGACTATTCGGAGCTTCTGTCACAGGATATAGATGCTTTCCCTACCTACTTTGCACCT :  465
B71    : TCGCAGGCCGGAGTTTTCGTCGGCAATATGGGAGTGGACTATTCGGAGCTTTTGTCGCAGGATATAGATGCGTTTCCGACGTACTTTGCGCCC :  465
B2     : TCGCAGGCCGGAGTTTTCGTCGGCAATATGGGAGTGGACTACTCGGAGCTTTTGTCGCAGGATATAGATGCGTTTCCGACATACTTTGCGCCC :  465
Ref    : TCGCAGGCCGGAGTTTTCGTCGGCAATATGGGAGTGGACTACTCGGAGCTTTTGTCGCAGGATATAGATGCGTTTCCGACATACTTTGCGCCC :  465
70-15  : TCGCAGGCCGGAGTTTTCGTCGGCAATATGGGAGTGGACTACTCGGAGCTTTTGTCGCAGGATATAGATGCGTTTCCGACATACTTTGCGCCC :  465

SCAU-2 : GGAACAGCGAGGAGTATCCTGTCCAACAGAATCTCATACTTTTTCGACTTGCACGGTCCTTCCGTCACTGTCGATACGGCGTGTTCTTCTAGC :  558
NI907  : GGAACAGCGAGGAGTATCCTGTCCAACAGAATCTCATACTTTTTCGACTTGCACGGTCCTTCCGTCACTGTCGATACGGCGTGTTCTTCTAGC :  558
W97-11 : GGAACAGCGAGGAGTATCCTGTCCAACAGAATCTCATACTTTTTCGACTTGCACGGTCCTTCCGTCACTGTCGATACGGCGTGTTCTTCTAGC :  558
B71    : GGCACGGCGAGGAGCATCTTGTCGAACCGCATCTCGTACTTTTTTGACCTGCACGGCCCCTCGGTCACGGTCGATACCGCGTGCTCTTCCAGC :  558
B2     : GGGACGGCGAGGAGCATCTTGTCGAACCGGATCTCGTACTTTTTTGACCTGCACGGCCCCTCGGTCACGGTCGATACCGCATGCTCTTCCAGC :  558
Ref    : GGGACGGCGAGGAGCATCTTGTCGAACCGGATCTCGTACTTTTTTGACCTGCACGGCCCCTCGGTCACGGTCGATACCGCATGCTCTTCCAGC :  558
70-15  : GGGACGGCGAGGAGCATCTTGTCGAACCGGATCTCGTACTTTTTTGACCTGCACGGCCCCTCGGTCACGGTCGATACCGCATGCTCTTCCAGC :  558

SCAU-2 : TTGGTGGCTGTTCACCAAGCGGTACAGAGCCTCCGCCTTGGCGAGACACCGGTTGCTATCGTCTGCGGCGCCAACCTCTTGCTGGGCCCTGCC :  651
NI907  : TTGGTGGCTGTTCACCAAGCGGTACAGAGCCTCCGCCTTGGCGAGACACCGGTTGCTATCGTCTGCGGCGCCAACCTCTTGCTGGGCCCTGCC :  651
W97-11 : TTGGTGGCTGTTCACCAAGCGGTACAGAGCCTCCGCCTTGGCGAGACACCGGTTGCTATCGTCTGCGGCGCCAACCTCTTGCTGGGCCCTGCC :  651
B71    : TTGGTGGCCGTCCACCAGGCGGTGCAGAGCCTCCGCCTCGGCGAGACCCCGGTTGCCATCGTCTGCGGCGCCAACCTGCTGCTGGGGCCGGCC :  651
B2     : TTGGTGGCCGTCCACCAGGCGGTGCAGAGCCTCCGCCTCGGCGAGACCCCGGTTGCCATCGTCTGCGGCGCCAACCTGCTGCTGGGGCCGGCC :  651
Ref    : TTGGTGGCCGTCCACCAGGCGGTGCAGAGCCTCCGCCTCGGCGAGACCCCGGTTGCCATCGTCTGCGGCGCCAACCTGCTGCTGGGGCCGGCC :  651
70-15  : TTGGTGGCCGTCCACCAGGCGGTGCAGAGCCTCCGCCTCGGCGAGACCCCGGTTGCCATCGTCTGCGGCGCCAACCTGCTGCTGGGGCCGGCC :  651

SCAU-2 : CAATATATTGCTGAGAGCAAGCTTCAGATGCTTTCTCCAAACGGCCGCTCACGCATGTGGGATGCATCAGCTGATGGGTATGCTCGCGGCGAG :  744
NI907  : CAATATATTGCTGAGAGCAAGCTTCAGATGCTTTCTCCAAACGGCCGCTCACGCATGTGGGATGCATCAGCTGATGGGTATGCTCGCGGCGAG :  744
W97-11 : CAATATATTGCTGAGAGCAAGCTTCAGATGCTTTCTCCAAACGGCCGCTCACGCATGTGGGATGCATCAGCTGATGGGTATGCTCGCGGCGAG :  744
B71    : CAGTATATTGCCGAGAGCAAGCTCCAGATGCTTTCTCCAAACGGCCGCTCGCGCATGTGGGATGCGTCAGCGGACGGATATGCTCGTGGCGAG :  744
B2     : CAGTATATTGCCGAGAGCAAGCTCCAGATGCTTTCTCCAAACGGCCGCTCGCGCATGTGGGATGCGTCGGCGGACGGATATGCTCGTGGCGAG :  744
Ref    : CAGTATATTGCCGAGAGCAAGCTCCAGATGCTTTCTCCAAACGGCCGCTCGCGCATGTGGGATGCGTCGGCGGACGGATATGCTCGTGGCGAG :  744
70-15  : CAGTATATTGCCGAGAGCAAGCTCCAGATGCTTTCTCCAAACGGCCGCTCGCGCATGTGGGATGCGTCGGCGGACGGATATGCTCGTGGCGAG :  744
```

```
SCAU-2 : GGCTTTGCCTCCATTGTGCTAAAGCCACTTAGTGCCGCTTTGGCCAACGGCGACCATATCGAATGCATTATTCGAGAGACTGGTTGTAATCAG :  837
NI907  : GGCTTTGCCTCCATTGTGCTAAAGCCACTTAGTGCCGCTTTGGCCAACGGCGACCATATCGAATGCATTATTCGAGAGACTGGTTGTAATCAG :  837
W97-11 : GGCTTTGCCTCCATTGTGCTAAAGCCACTTAGTGCCGCTTTGGCCAACGGCGACCATATCGAATGCATTATTCGAGAGACTGGTTGTAATCAG :  837
B71    : GGCTTTGCCTCGATTGTGCTGAAGCCGCTCAGCGTCGCCTTGGCTAACGGCGACCATATCGAATGCATTATTCGAGAGACTGGTTGTAATCAG :  837
B2     : GGCTTTGCCTCCATTGTGCTGAAGCCGCTCAGCGTCGCCTTGGCTAACGGTGACCATATCGAGTGCATTATTCGAGAGACTGGTTGTAATCAG :  837
Ref    : GGCTTTGCCTCCATTGTGCTGAAGCCGCTCAGCGTCGCCTTGGCTAACGGTGACCATATCGAGTGCATTATTCGAGAGACTGGTTGTAATCAG :  837
70-15  : GGCTTTGCCTCCATTGTGCTGAAGCCGCTCAGCGTCGCCTTGGCTAACGGTGACCATATCGAGTGCATTATTCGAGAGACTGGTTGTAATCAG :  837

SCAU-2 : GATGGCCGGACTAAAGgtacaagaaagcccttcgttttatctcaatttttcaggccaattttgcgatggcaggacgggaagatgtgctgactt :  930
NI907  : GATGGCCGGACTAAAGgtacaagaaagcccttcgttttatctcaatttttcaggccaattttgcgatggcaggacgggaagatgtgctgactt :  930
W97-11 : GATGGCCGGACTAAAGgtacaagaaagcccttcgttttatctcaatttttcaggccaattttgcgatggcaggacgggaagatgtgctgactt :  930
B71    : GATGGTCGGACGAAAGgtacaaatgaccccatttgttggtgtttgagttttgacgt-----------------------aaaagatggggttg :  907
B2     : GATGGTCGGACTAAAGgtacaaatgaccccatttgttggtgtttgagttttgacgt-----------------------aaaagatggggttg :  907
Ref    : GATGGTCGGACTAAAGgtacaaatgaccccatttgttggtgtttgagttttgacgt-----------------------aaaagatggggttg :  907
70-15  : GATGGTCGGACTAAAGgtacaaatgaccccatttgttggtgtttgagttttgacgt-----------------------aaaagatggggttg :  907

SCAU-2 : ttttttctactctaatgtgaacaaacacaaaagGTATCACAATGCCCAGTCCACTTGCCCAGTGCAAGCTCATACAGGAGACATACAGGCGTG : 1023
NI907  : ttttttctactctaatgtgaacaaacacaaaagGTATCACAATGCCCAGTCCACTTGCCCAGTGCAAGCTCATACAGGAGACATACAGGCGTG : 1023
W97-11 : ttttttctactctaatgtgaacaaacacaaaagGTATCACAATGCCCAGTCCACTTGCCCAGTGCAAGCTCATACAGGAGACATACAGGCGTG : 1023
B71    : gctaattttcttgattgtgaacaaacacaatagGCATCACAATGCCCAGTCCACTCGCTCAATGCAAACTCATACAGGAGACGTACAAGCGCG : 1000
B2     : gctaattttcttgattgtgaacaaacacaaaagGCATTACAATGCCCAGTCCACTCGCTCAATGCAAACTCATCCAGGAGACGTATAAGCGCG : 1000
Ref    : gctaattttcttgattgtgaacaaacacaaaagGCATTACAATGCCCAGTCCACTCGCTCAATGCAAACTCATCCAGGAGACGTATAAGCGCG : 1000
70-15  : gctaattttcttgattgtgaacaaacacaaaagGCATTACAATGCCCAGTCCACTCGCTCAATGCAAACTCATCCAGGAGACGTATAAGCGCG : 1000

SCAU-2 : CAGGCCTCGACCTGAGCAAGAGCTCAGACAGGCCGCAGTATTTCGAGGCACATGGTACCGGCACCCCAGCGGGAGATCCTGTGGAGGCAGAAG : 1116
NI907  : CAGGCCTCGACCTGAGCAAGAGCTCAGACAGGCCGCAGTATTTCGAGGCACATGGTACCGGCACCCCAGCGGGAGATCCTGTGGAGGCAGAAG : 1116
W97-11 : CAGGCCTCGACCTGAGCAAGAGCTCAGACAGGCCGCAGTATTTCGAGGCACATGGTACCGGCACCCCAGCGGGAGATCCTGTGGAGGCAGAAG : 1116
B71    : CGGGCCTGGACCTCAGCAAAAGCTCAGACAGGCCGCAGTATTTCGAGGCGCACGGCACCGGCACCCCGGCGGGAGATCCCGTCGAGGCGGAAG : 1093
B2     : CGGGCCTGGACCTCAGCAAAAGCTCAGACAGGCCGCAGTATTTCGAGGCGCACGGCACCGGCACCCCGGCGGGAGATCCCGTCGAGGCGGAAG : 1093
Ref    : CGGGCCTGGACCTCAGCAAAAGCTCAGACAGGCCGCAGTATTTCGAGGCGCACGGCACCGGCACCCCGGCGGGAGATCCCGTCGAGGCGGAAG : 1093
70-15  : CGGGCCTGGACCTCAGCAAAAGCTCAGACAGGCCGCAGTATTTCGAGGCGCACGGCACCGGCACCCCGGCGGGAGATCCCGTCGAGGCGGAAG : 1093
```

```
SCAU-2 : CCATCAGCACGGCATTCTTTGGTCCGCAGAGTGGTTATTGCAGAAAATCCGACGATCCGAAACTCTACGTGGGGTCGGTCAAAACTGTGATTG : 1209
NI907  : CCATCAGCACGGCATTCTTTGGTCCGCAGAGTGGTTATTGCAGAAAATCCGACGATCCGAAACTCTACGTGGGGTCGGTCAAAACTGTGATTG : 1209
W97-11 : CCATCAGCACGGCATTCTTTGGTCCGCAGAGTGGTTATTGCAGAAAATCCGACGATCCGAAACTCTACGTGGGGTCGGTCAAAACTGTGATTG : 1209
B71    : CCATCAGCACGGCATTCTTCGGTCCCGAGAGCGGCTTTCGTAGAACATCCCACGACCCAAAACTCTACGTCGGGTCGGTCAAGACCGTGATTG : 1186
B2     : CCATCAGCACGGCATTTTTTGGTCCCGAGAGCGGCTTTCGTAGAACATCCCACGACCCAAAACTCTACGTCGGGTCGGTCAAGACTGTGATTG : 1186
Ref    : CCATCAGCACGGCATTTTTTGGTCCCGAGAGCGGCTTTCGTAGAACATCCCACGACCCAAAACTCTACGTCGGGTCGGTCAAGACTGTGATTG : 1186
70-15  : CCATCAGCACGGCATTTTTTGGTCCCGAGAGCGGCTTTCGTAGAACATCCCACGACCCAAAACTCTACGTCGGGTCGGTCAAGACTGTGATTG : 1186

SCAU-2 : GCCATACAGAAGGCACTGCTGGCCTCGCTGGCTTGATTAAAGCTTCCCTTGCCATGAAAGCTAAAAGCATCCCGCCGAACTTGCACCTGGAAC : 1302
NI907  : GCCATACAGAAGGCACTGCTGGCCTCGCTGGCTTGATTAAAGCTTCCCTTGCCATGAAAGCTAAAAGCATCCCGCCGAACTTGCACCTGGAAC : 1302
W97-11 : GCCATACAGAAGGCACTGCTGGCCTCGCTGGCTTGATTAAAGCTTCCCTTGCCATGAAAGCTAAAAGCATCCCGCCGAACTTGCACCTGGAAC : 1302
B71    : GGCACACAGAAGGCACGGCCGGACTCGCCGGCCTGATCAAGGCTTCGCTCGCCATGAAGGCCAAGAGCATCCCGCCGAATCTGCACCTGGAGC : 1279
B2     : GGCACACAGAAGGCACGGCCGGACTCGCCGGCCTGATCAAGGCCTCGCTCGCCATGAAGGCCAAGAGCATCCCGCCGAACCTGCACCTGGAGC : 1279
Ref    : GGCACACAGAAGGCACGGCCGGACTCGCCGGCCTGATCAAGGCCTCGCTCGCCATGAAGGCCAAGAGCATCCCGCCGAACCTGCACCTGGAGC : 1279
70-15  : GGCACACAGAAGGCACGGCCGGACTCGCCGGCCTGATCAAGGCCTCGCTCGCCATGAAGGCCAAGAGCATCCCGCCGAACCTGCACCTGGAGC : 1279

SCAU-2 : GCGTGAACCCAGCTGTTCAGCCGTTTTATGGAAACCTTGAGATCCCGACACGGCTAATGGACTGGCCCGAGCCCGAGCCCGGCCAGCCCCTCC : 1395
NI907  : GCGTGAACCCAGCTGTTCAGCCGTTTTATGGAAACCTTGAGATCCCGACACGGCTAATGGACTGGCCCGAGCCCGAGCCCGGCCAGCCCCTCC : 1395
W97-11 : GCGTGAACCCAGCTGTTCAGCCGTTTTATGGAAACCTTGAGATCCCGACACGGCTAATGGACTGGCCCGAGCCCGAGCCCGGCCAGCCCCTCC : 1395
B71    : GCGTGAACCCCGCCGTCCAGCCGTTTTATGGAAACCTCGAAATCCCGACGCGGCTGATGGACTGGCCCGAGCCCGCGCCCGGCCAGCCGCTCC : 1372
B2     : GCGTGAACCCCGCCGTCCAGCCGTTTTATGGAAACCTCGAAATCCCGACGCGGCTGATGGACTGGCCCGAGCCCGCGCCCGGCCAGCCGCTCC : 1372
Ref    : GCGTGAACCCCGCCGTCCAGCCGTTTTATGGAAACCTCGAAATCCCGACGCGGCTGATGGACTGGCCCGAGCCCGCGCCCGGCCAGCCGCTCC : 1372
70-15  : GCGTGAACCCCGCCGTCCAGCCGTTTTATGGAAACCTCGAAATCCCGACGCGGCTGATGGACTGGCCCGAGCCCGCGCCCGGCCAGCCGCTCC : 1372

SCAU-2 : GTGCTAGCGTTAACAGCTTTGGATTTGGTGGCGCCAACGCGCACGTCATCTTGGAGAGTTATACACCGCCGGCCGT------TGCAGCTCTCC : 1482
NI907  : GTGCTAGCGTTAACAGCTTTGGATTTGGTGGCGCCAACGCGCACGTCATCTTGGAGAGTTATACACCGCCGGCCGT------TGCAGCTCTCC : 1482
W97-11 : GTGCTAGCGTTAACAGCTTTGGATTTGGTGGCGCCAACGCGCACGTCATCTTGGAGAGTTATACACCGCCGGCCGT------TGCAGCTCTCC : 1482
B71    : GTGCCAGCGTCAACAGCTTCGGGTTTGGCGGCGCCAACGCGCACGTCATCCTGGAGAGCTACAACCCGCCGGCGGCCGAGGTTGCGATGCTCC : 1465
B2     : GTGCCAGCGTCAACAGCTTCGGGTTTGGCGGCGCCAACGCGCACGTCATCTTGGAGAGCTACACG---CCGGCGGCCGAGGTTGCGATGGTCA : 1462
Ref    : GTGCCAGCGTCAACAGCTTCGGGTTTGGCGGCGCCAACGCGCACGTCATCTTGGAGAGCTACACG---CCGGCGGCCGAGGTTGCGATGGTCA : 1462
70-15  : GTGCCAGCGTCAACAGCTTCGGGTTTGGCGGCGCCAACGCGCACGTCATCTTGGAGAGCTACACG---CCGGCGGCCGAGGTTGCGATGGTCA : 1462
```

```
SCAU-2 : CTCCCATGCCGACGTCTGGCCCTGTTTTCTCCCCTTTTGTCTTTTCCGCCAGTTCAGATAAAGCATTAGCAGGAATATTATCTGCCTACGGCG : 1575
NI907  : CTCCCATGCCGACGTCTGGCCCTGTTTTCTCCCCTTTTGTCTTTTCCGCCAGTTCAGATAAAGCATTAGCAGGAATATTATCTGCCTACGGCG : 1575
W97-11 : CTCCCATGCCGACGTCTGGCCCTGTTTTCTCCCCTTTTGTCTTTTCCGCCAGTTCAGATAAAGCATTAGCAGGAATATTATCTGCCTACGGCG : 1575
B71    : CTCCCACGGCTGCCGCTGGCCCCGTCTTCTCCCCTTTTGTCTTTTCGGCCAGCTCAGATAAGGCACTAGCAAGCATGCTATCCGCCTACAGCG : 1558
B2     : CTCCCACGGCTGCCGCTGGCCCCGTCTTTTCCCCTTTTGTCTTTTCGGCCAGCTCGGATAAGGCACTAGCAAGCATGCTATCTGCCTATAGCG : 1555
Ref    : CTCCCACGGCTGCCGCTGGCCCCGTCTTTTCCCCTTTTGTCTTTTCGGCCAGCTCGGATAAGGCACTAGCAAGCATGCTATCTGCCTATAGCG : 1555
70-15  : CTCCCACGGCTGCCGCTGGCCCCGTCTTTTCCCCTTTTGTCTTTTCGGCCAGCTCGGATAAGGCACTAGCAAGCATGCTATCTGCCTATAGCG : 1555

SCAU-2 : AGTATCTAAGTCAGCATCCCACGGTTGACCTACGATCCGTTGCATATACTTTGAGTCAGCATCGCTCCATTTTCGACAAGCGAGCCGTGATTT : 1668
NI907  : AGTATCTAAGTCAGCATCCCACGGTTGACCTACGATCCGTTGCATATACTTTGAGTCAGCATCGCTCCATTTTCGACAAGCGAGCCGTGATTT : 1668
W97-11 : AGTATCTAAGTCAGCATCCCACGGTTGACCTACGATCCGTTGCATATACTTTGAGTCAGCATCGCTCCATTTTCGACAAGCGAGCCGTGATTT : 1668
B71    : ATTACCTGAGTCTGAACCCCACGGTCGACCTGCGGTCCGTCGCCTATACCCTCAGTCAGCATCGCTCCATTTTCGACAAGCGAGCCGCAATCT : 1651
B2     : ATTACCTGAGTCTTAATCCCACGGTTGACCTGCGGTCCGTCGCCTATACCCTCAGTCAGCATCGCTCCGTTTTCGACAAGCGAGCCGCAATCT : 1648
Ref    : ATTACCTGAGTCTTAATCCCACGGTTGACCTGCGGTCCGTCGCCTATACCCTCAGTCAGCATCGCTCCGTTTTCGACAAGCGAGCCGCAATCT : 1648
70-15  : ATTACCTGAGTCTTAATCCCACGGTTGACCTGCGGTCCGTCGCCTATACCCTCAGTCAGCATCGCTCCGTTTTCGACAAGCGAGCCGCAATCT : 1648

SCAU-2 : CTGCAGCCGACCTTGACACCCTCAAGTCCAAGCTCAAGGCACGCAGCGAGGAAGCCAGTCCCAGCGCCAAG------GCTGTTCAATCTCTCG : 1755
NI907  : CTGCAGCCGACCTTGACACCCTCAAGTCCAAGCTCAAGGCACGCAGCGAGGAAGCCAGTCCCAGCGCCAAG------GCTGTTCAATCTCTCG : 1755
W97-11 : CTGCAGCCGACCTTGACACCCTCAAGTCCAAGCTCAAGGCACGCAGCGAGGAAGCCAGTCCCAGCGCCAAG------GCTGTTCAATCTCTCG : 1755
B71    : CTGCACCCGATCTCGACACCCTCAAGACCAAGCTCAAGGCACGCAGTGAGGAAGCCAGTCCTAGCGGCAAGACAGCAGCAGTCCAGTCTCTGG : 1744
B2     : CTGCACCCGATCTCGACACCCTCAAGACCAAGCTCAAGGCACGCAGTGAGGAAGCCAGTCCTAGCGGCAAGACAGCAGCAGTCCAGTCTCTGG : 1741
Ref    : CTGCACCCGATCTCGACACCCTCAAGACCAAGCTCAAGGCACGCAGTGAGGAAGCCAGTCCTAGCGGCAAGACAGCAGCAGTCCAGTCTCTGG : 1741
70-15  : CTGCACCCGATCTCGACACCCTCAAGACCAAGCTCAAGGCACGCAGTGAGGAAGCCAGTCCTAGCGGCAAGACAGCAGCAGTCCAGTCTCTGG : 1741

SCAU-2 : AGCGACGTCCCCGCTACCTCGGCATCTTTACGGGTCAAGGAGCGCAGTGGGCAAGAATGGGAGTAGATATCATCACGGCATCTCCTGCAGCCA : 1848
NI907  : AGCGACGTCCCCGCTACCTCGGCATCTTTACGGGTCAAGGAGCGCAGTGGGCAAGAATGGGAGTAGATATCATCACGGCATCTCCTGCAGCCA : 1848
W97-11 : AGCGACGTCCCCGCTACCTCGGCATCTTTACGGGTCAAGGAGCGCAGTGGGCAAGAATGGGAGTAGATATCATCACGGCATCTCCTGCAGCCA : 1848
B71    : AGCGACGTCCCCGCTACCTGGGCGTCTTTACGGGTCAAGGAGCGCAGTGGGCACGGATGGGCGTCGATGTTATCAATGCATCTCCTGCAGCGA : 1837
B2     : AGCGACGTCCCCGCTACCTGGGCGTCTTTACGGGTCAAGGAGCGCAGTGGGCACGGATGGGCGTCGATGTTATCAATGCATCTCCTGCAGCGA : 1834
Ref    : AGCGACGTCCCCGCTACCTGGGCGTCTTTACGGGTCAAGGAGCGCAGTGGGCACGGATGGGCGTCGATGTTATCAATGCATCTCCTGCAGCGA : 1834
70-15  : AGCGACGTCCCCGCTACCTGGGCGTCTTTACGGGTCAAGGAGCGCAGTGGGCACGGATGGGCGTCGATGTTATCAATGCATCTCCTGCAGCGA : 1834
```

```
SCAU-2 : GAGCCATTTTTGAAGAGCTGGAACAGAGCCTCCAGACGTTGCCCGAAAAGGAGCGACCGTCTTGGTCCATGTTGAAAGAGCTACTCGCGCCCC :  1941
NI907  : GAGCCATTTTTGAAGAGCTGGAACAGAGCCTCCAGACGTTGCCCGAAAAGGAGCGACCGTCTTGGTCCATGTTGAAAGAGCTACTCGCGCCCC :  1941
W97-11 : GAGCCATTTTTGAAGAGCTGGAACAGAGCCTCCAGACGTTGCCCGAAAAGGAGCGACCGTCTTGGTCCATGTTGAAAGAGCTACTCGCGCCCC :  1941
B71    : GGGCTATTTTTGAAGACCTGGAACAGAGCCTCAAAACGCTGCCCGAAGAGGACCGACCATCCTGGTCCATGCTGGAGGAGCTGCTGGCGCCCC :  1930
B2     : GGGCTATTTTTGAAGACCTGGAACAGAGCCTCAAAACCCTGCCCGAAGAGGACCGACCATCTTGGTCCATGCTGGAGGAGCTGCTGGCGCCCC :  1927
Ref    : GGGCTATTTTTGAAGACCTGGAACAGAGCCTCAAAACCCTGCCCGAAGAGGACCGACCATCTTGGTCCATGCTGGAGGAGCTGCTGGCGCCCC :  1927
70-15  : GGGCTATTTTTGAAGACCTGGAACAGAGCCTCAAAACCCTGCCCGAAGAGGACCGACCATCTTGGTCCATGCTGGAGGAGCTGCTGGCGCCCC :  1927

SCAU-2 : CGGAGACTTCGCGCGTTTATCAGGCCCATATCTCACAGACCGTCTGCACTGCGGTCCAAATCCTGCTCGTCCAGCTTTTGCGCGCAGCGGGAG :  2034
NI907  : CGGAGACTTCGCGCGTTTATCAGGCCCATATCTCACAGACCGTCTGCACTGCGGTCCAAATCCTGCTCGTCCAGCTTTTGCGCGCAGCGGGAG :  2034
W97-11 : CGGAGACTTCGCGCGTTTATCAGGCCCATATCTCACAGACCGTCTGCACTGCGGTCCAAATCCTGCTCGTCCAGCTTTTGCGCGCAGCGGGAG :  2034
B71    : CCGAGACTTCGCGTGTCTACCAGGCCAACATCTCACAGACCGTCTGCACTGCCGTCCAAGTCATGATGGTGCAGCTCCTGCGCGCCGCCGGCA :  2023
B2     : CCGAGACTTCGCGCGTCTACCAGGCCAACATCTCACAGACCGTCTGCACTGCCGTCCAAGTCATGATGGTGCAGCTCCTGCGCGCCGCCGGCA :  2020
Ref    : CCGAGACTTCGCGCGTCTACCAGGCCAACATCTCACAGACCGTCTGCACTGCCGTCCAAGTCATGATGGTGCAGCTCCTGCGCGCCGCCGGCA :  2020
70-15  : CCGAGACTTCGCGCGTCTACCAGGCCAACATCTCACAGACCGTCTGCACTGCCGTCCAAGTCATGATGGTGCAGCTCCTGCGCGCCGCCGGCA :  2020

SCAU-2 : TTGAGTTTTCGTGCGTGGTAGGCCATTCTTCGGGTGAAATAGCTGCTGCGTACACAGCTGGTTATCTGTCCGCCAAGGATGCCGTGCGCGCCG :  2127
NI907  : TTGAGTTTTCGTGCGTGGTAGGCCATTCTTCGGGTGAAATAGCTGCTGCGTACACAGCTGGTTATCTGTCCGCCAAGGATGCCGTGCGCGCCG :  2127
W97-11 : TTGAGTTTTCGTGCGTGGTAGGCCATTCTTCGGGTGAAATAGCTGCTGCGTACACAGCTGGTTATCTGTCCGCCAAGGATGCCGTGCGCGCCG :  2127
B71    : TCGAATTTTCGTGCGTCGTGGGCCATTCCTCGGGCGAGATGGCGGCTGCGTACACGGCCGGCTACCTGTCTGCCAGGGACGCCGTGCGCGCCG :  2116
B2     : TCGAATTTTCGTGCGTCGTGGGCCATTCCTCGGGCGAGATGGCGGCTGCGTACACGGCCGGCTACCTGTCTGCCAGGGATGCCGTGCGCGCCG :  2113
Ref    : TCGAATTTTCGTGCGTCGTGGGCCATTCCTCGGGCGAGATGGCGGCTGCGTACACGGCCGGCTACCTGTCTGCCAGGGATGCCGTGCGCGCCG :  2113
70-15  : TCGAATTTTCGTGCGTCGTGGGCCATTCCTCGGGCGAGATGGCGGCTGCGTACACGGCCGGCTACCTGTCTGCCAGGGATGCCGTGCGCGCCG :  2113

SCAU-2 : CATATTTTCGAGGTGTCCACACGCATTTGGCAAAAGGGGCAAATGGCCAACCGGGAGGCATGATTGCGGTCGGAACAACCCTTGAGGACGCCA :  2220
NI907  : CATATTTTCGAGGTGTCCACACGCATTTGGCAAAAGGGGCAAATGGCCAACCGGGAGGCATGATTGCGGTCGGAACAACCCTTGAGGACGCCA :  2220
W97-11 : CATATTTTCGAGGTGTCCACACGCATTTGGCAAAAGGGGCAAATGGCCAACCGGGAGGCATGATTGCGGTCGGAACAACCCTTGAGGACGCCA :  2220
B71    : CGTACTTTCGAGGCGTCCACTCCCAGTTGGCAAAAGGGTCAAACGGCCAGCCGGGGGGCATGATTGCGGTCGGCACGAATTTCGAGGACGCCG :  2209
B2     : CGTACTTTCGAGGCGTCCACTCGCAGTTGGCAAAAGGGTCAAACGGCCAGCCGGGCGGCATGATTGCGGTCGGCACGAATTTCGAGGACGCCG :  2206
Ref    : CGTACTTTCGAGGCGTCCACTCGCAGTTGGCAAAAGGGTCAAACGGCCAGCCGGGCGGCATGATTGCGGTCGGCACGAATTTCGAGGACGCCG :  2206
70-15  : CGTACTTTCGAGGCGTCCACTCGCAGTTGGCAAAAGGGTCAAACGGCCAGCCGGGCGGCATGATTGCGGTCGGCACGAATTTCGAGGACGCCG :  2206
```

```
SCAU-2 : AGGAGCTTTGCGAAGTCGACGACTTCAAGGGACGCTTATGCGTCGCCGCCAGCAATTCGAACGACAGCGTAACGCTTTCGGGTGATCTTGATG :  2313
NI907  : AGGAGCTTTGCGAAGTCGACGACTTCAAGGGACGCTTATGCGTCGCCGCCAGCAATTCGAACGACAGCGTAACGCTTTCGGGTGATCTTGATG :  2313
W97-11 : AGGAGCTTTGCGAAGTCGACGACTTCAAGGGACGCTTATGCGTCGCCGCCAGCAATTCGAACGACAGCGTAACGCTTTCGGGTGATCTTGATG :  2313
B71    : AGGAGCTGTGCGAGCTCGACGACTTCAAGGGCCGCTTGTGCGTCGCCGCAAGCAATTCGGCCGAGCTCGTGACGCTCTCGGGCGACCTTGATG :  2302
B2     : AGGAGCTGTGCGAGCTCGACGACTTCAAGGGCCGCTTGTGCGTCGCCGCAAGCAACTCGGCCGAGCTCGTGACGCTCTCGGGCGACCTTGATG :  2299
Ref    : AGGAGCTGTGCGAGCTCGACGACTTCAAGGGCCGCTTGTGCGTCGCCGCAAGCAACTCGGCCGAGCTCGTGACGCTCTCGGGCGACCTTGATG :  2299
70-15  : AGGAGCTGTGCGAGCTCGACGACTTCAAGGGCCGCTTGTGCGTCGCCGCAAGCAACTCGGCCGAGCTCGTGACGCTCTCGGGCGACCTTGATG :  2299

SCAU-2 : CTGTGAAAGAGGTTAAAAAGGTCCTGGACGCCGAGGGAAAATTCAACAAGCAACTTCAGGTTGATAAGGGATACCACTCGCACCATATGTTAC :  2406
NI907  : CTGTGAAAGAGGTTAAAAAGGTCCTGGACGCCGAGGGAAAATTCAACAAGCAACTTCAGGTTGATAAGGGATACCACTCGCACCATATGTTAC :  2406
W97-11 : CTGTGAAAGAGGTTAAAAAGGTCCTGGACGCCGAGGGAAAATTCAACAAGCAACTTCAGGTTGATAAGGGATACCACTCGCACCATATGTTAC :  2406
B71    : CCGTGCAAGAGGTCAAGAAGATCCTGGACGCCGAGGAGAAGTTCAACAAGCAGCTTCAGGTCGATAAGGGATACCACTCGCATCACATGCTGC :  2395
B2     : CCGTGCAAGAGGTCAAGAAGATCCTGGACGCCGAGGAGAAGTTCAACAAGCAGCTTCAGGTCGACAAGGGATACCACTCGCACCACATGCTGC :  2392
Ref    : CCGTGCAAGAGGTCAAGAAGATCCTGGACGCCGAGGAGAAGTTCAACAAGCAGCTTCAGGTCGACAAGGGATACCACTCGCACCACATGCTGC :  2392
70-15  : CCGTGCAAGAGGTCAAGAAGATCCTGGACGCCGAGGAGAAGTTCAACAAGCAGCTTCAGGTCGACAAGGGATACCACTCGCACCACATGCTGC :  2392

SCAU-2 : CCTGCAGTGAGCCGTATATCACTTCTTTGCAAAATTGTGATATTCAAGCTCGGGTGCCGGGCGACGCCAAAGCATGCCGCTGGATTTCGAGCG :  2499
NI907  : CCTGCAGTGAGCCGTATATCACTTCTTTGCAAAATTGTGATATTCAAGCTCGGGTGCCGGGCGACGCCAAAGCATGCCGCTGGATTTCGAGCG :  2499
W97-11 : CCTGCAGTGAGCCGTATATCACTTCTTTGCAAAATTGTGATATTCAAGCTCGGGTGCCGGGCGACGCCAAAGCATGCCGCTGGATTTCGAGCG :  2499
B71    : CGTGCAGCGAGCCGTACGTCGCGTCTCTGCAAAAGTGCGGCATCCAAGCTCAGGTGCCGGGCGATGCGACGGCGTGCCGCTGGATCTCGAGCG :  2488
B2     : CGTGCAGCGAGCCGTACGTCGCGTCTCTGCAAAAGTGCGGCATCCAAGCGCAGGTGCCGGGCGATGCGACGGCGTGCCGCTGGATCTCGAGCG :  2485
Ref    : CGTGCAGCGAGCCGTACGTCGCGTCTCTGCAAAAGTGCGGCATCCAAGCGCAGGTGCCGGGCGATGCGACGGCGTGCCGCTGGATCTCGAGCG :  2485
70-15  : CGTGCAGCGAGCCGTACGTCGCGTCTCTGCAAAAGTGCGGCATCCAAGCGCAGGTGCCGGGCGATGCGACGGCGTGCCGCTGGATCTCGAGCG :  2485

SCAU-2 : TCTATGTTGATGATATGGCCTCACTCGACTGCCGAGTCCAGGACAAATATTGGGTTGAGAATTTGGCCAAGCCTGTGCTCTTTTCTCAGGCGC :  2592
NI907  : TCTATGTTGATGATATGGCCTCACTCGACTGCCGAGTCCAGGACAAATATTGGGTTGAGAATTTGGCCAAGCCTGTGCTCTTTTCTCAGGCGC :  2592
W97-11 : TCTATGTTGATGATATGGCCTCACTCGACTGCCGAGTCCAGGACAAATATTGGGTTGAGAATTTGGCCAAGCCTGTGCTCTTTTCTCAGGCGC :  2592
B71    : TCTACGTTGATGACATGACCAATCTGGACTGCCGAGTCCAGGACAGATACTGGATTGAGAATCTGGCCAAGCCCGTCATGTTTTCTCAGGCGC :  2581
B2     : TCTACGTTGATGACATGACCAATCTGGACTGCCGAGTCCAGGACAGATACTGGATTGAGAATCTGGCCAAGCCCGTCATGTTTTCCCAGGCGC :  2578
Ref    : TCTACGTTGATGACATGACCAATCTGGACTGCCGAGTCCAGGACAGATACTGGATTGAGAATCTGGCCAAGCCCGTCATGTTTTCCCAGGCGC :  2578
70-15  : TCTACGTTGATGACATGACCAATCTGGACTGCCGAGTCCAGGACAGATACTGGATTGAGAATCTGGCCAAGCCCGTCATGTTTTCCCAGGCGC :  2578
```

```
SCAU-2 : TGTCCTATGCCCTGGGCGCTGATGACAAGTTTGATTGTGTTATCGAGGTTGGCCCTCACCCAGCGCTGAAGGGACCGGCCAGCCAGATAATCC :  2685
NI907  : TGTCCTATGCCCTGGGCGCTGATGACAAGTTTGATTGTGTTATCGAGGTTGGCCCTCACCCAGCGCTGAAGGGACCGGCCAGCCAGATAATCC :  2685
W97-11 : TGTCCTATGCCCTGGGCGCTGATGACAAGTTTGATTGTGTTATCGAGGTTGGCCCTCACCCAGCGCTGAAGGGACCGGCCAGCCAGATAATCC :  2685
B71    : TCTCCCATGCCCTGGGCGGCGATGACAAGTTTGACTGCGTCATCGAGGTCGGCCCGCACCCGGCGCTCAAGGGACCAGCCAGCCAGACGATCC :  2674
B2     : TCTCCCATGCCCTGGGCGGCGATGACAAGTTTGACTCCGTCATCGAGGTCGGCCCGCACCCGGCGCTCAAGGGACCAGCCAGCCAGACCATCC :  2671
Ref    : TCTCCCATGCCCTGGGCGGCGATGACAAGTTTGACTCCGTCATCGAGGTCGGCCCGCACCCGGCGCTCAAGGGACCAGCCAGCCAGACCATCC :  2671
70-15  : TCTCCCATGCCCTGGGCGGCGATGACAAGTTTGACTCCGTCATCGAGGTCGGCCCGCACCCGGCGCTCAAGGGACCAGCCAGCCAGACCATCC :  2671

SCAU-2 : AGAGTTGCCTTGGTGAAAAGCTGCCGTATTTTGGATGCTTAAACAGAGGCACCAACAGCAACGAAGCCATGGCAGAATGTCTGGGTGGTATTT :  2778
NI907  : AGAGTTGCCTTGGTGAAAAGCTGCCGTATTTTGGATGCTTAAACAGAGGCACCAACAGCAACGAAGCCATGGCAGAATGTCTGGGTGGTATTT :  2778
W97-11 : AGAGTTGCCTTGGTGAAAAGCTGCCGTATTTTGGATGCTTAAACAGAGGCACCAACAGCAACGAAGCCATGGCAGAATGTCTGGGTGGTATTT :  2778
B71    : AGGCCTGCCTCGGCGAAAGGCTGCCGTATTTTGGATGCCTGAGCAGGGGCACCGACAGCAACGAAGCCTTTGCAGAGTTTTTGGGTGGGGTCT :  2767
B2     : AGGCCTGCCTCGGCGAAAGGCTGCCGTATTTTGGATGCCTGAGCAGGGGCACCGACAGCAACGAAGCCTTTGCAGAGTTTTTGGGTGGGGTCT :  2764
Ref    : AGGCCTGCCTCGGCGAAAGGCTGCCGTATTTTGGATGCCTGAGCAGGGGCACCGACAGCAACGAAGCCTTTGCAGAGTTTTTGGGTGGGGTCT :  2764
70-15  : AGGCCTGCCTCGGCGAAAGGCTGCCGTATTTTGGATGCCTGAGCAGGGGCACCGACAGCAACGAAGCCTTTGCAGAGTTTTTGGGTGGGGTCT :  2764

SCAU-2 : GGTCGTCCTTTGGTAGTTCGGCCGTCAACCTTGCCGCCTACGAAAAGTTTGCCTCTGGCAATTGTGACCAACGCCTACTCAAAGAGCTTCCAT :  2871
NI907  : GGTCGTCCTTTGGTAGTTCGGCCGTCAACCTTGCCGCCTACGAAAAGTTTGCCTCTGGCAATTGTGACCAACGCCTACTCAAAGAGCTTCCAT :  2871
W97-11 : GGTCGTCCTTTGGTAGTTCGGCCGTCAACCTTGCCGCCTACGAAAAGTTTGCCTCTGGCAATTGTGACCAACGCCTACTCAAAGAGCTTCCAT :  2871
B71    : GGTCGACCTTTGGCAGTTCGGCCGTCGACCTGGCCGCCTATGAAAGATTTGCAACTGGCGGTTGTGACCAACGCTTGGTCAAAGAGCTCCCGT :  2860
B2     : GGTCGACCTTTGGCAGTTCGGCCGTCGACCTGGCCGCCTATGAAAGATTTGCAACTGGCGGCTGTGACCAACGCTTGGTCAAGGGGCTCCCGT :  2857
Ref    : GGTCGACCTTTGGCAGTTCGGCCGTCGACCTGGCCGCCTATGAAAGATTTGCAACTGGCGGCTGTGACCAACGCTTGGTCAAGGGGCTCCCGT :  2857
70-15  : GGTCGACCTTTGGCAGTTCGGCCGTCGACCTGGCCGCCTATGAAAGATTTGCAACTGGCGGCTGTGACCAACGCTTGGTCAAGGGGCTCCCGT :  2857

SCAU-2 : CTTACAAGTGGGATCATGACGTCGAGTATTATTTCCAATCTCGTCTCTCCAAGGTTGTGTTGCACAGAGGCAGCTCGCCCAATGAGTTGCTCG :  2964
NI907  : CTTACAAGTGGGATCATGACGTCGAGTATTATTTCCAATCTCGTCTCTCCAAGGTTGTGTTGCACAGAGGCAGCTCGCCCAATGAGTTGCTCG :  2964
W97-11 : CTTACAAGTGGGATCATGACGTCGAGTATTATTTCCAATCTCGTCTCTCCAAGGTTGTGTTGCACAGAGGCAGCTCGCCCAATGAGTTGCTCG :  2964
B71    : CCTACACGTGGGATCATGATGTCGAACATTATTTCCAGTCTCGCCTGTCCAAGGTCGTGCTGCACAGAAGCACACCGCCGAACGAGCTGCTCG :  2953
B2     : CCTACACGTGGGATCACGATGTCGAACACTATTTTCAGTCTCGCCTGTCCAAGGTCGTGCTGCACAGAAGCACACCGCCGAACGAGCTGCTCG :  2950
Ref    : CCTACACGTGGGATCACGATGTCGAACACTATTTTCAGTCTCGCCTGTCCAAGGTCGTGCTGCACAGAAGCACACCGCCGAACGAGCTGCTCG :  2950
70-15  : CCTACACGTGGGATCACGATGTCGAACACTATTTTCAGTCTCGCCTGTCCAAGGTCGTGCTGCACAGAAGCACACCGCCGAACGAGCTGCTCG :  2950
```

```
SCAU-2 : GCACTAGACTCCCCGATGACAGTGCGGCCGAGGTGCGGTGGCGGAACAGTCTAAATCCGGCAGAGGTTCCATGGTTGCTACAGCATTCGGCCC :  3057
NI907  : GCACTAGACTCCCCGATGACAGTGCGGCCGAGGTGCGGTGGCGGAACAGTCTAAATCCGGCAGAGGTTCCATGGTTGCTACAGCATTCGGCCC :  3057
W97-11 : GCACTAGACTCCCCGATGACAGTGCGGCCGAGGTGCGGTGGCGGAACAGTCTAAATCCGGCAGAGGTTCCATGGTTGCTACAGCATTCGGCCC :  3057
B71    : GCACCAGACTCCCCGATGATACTGCGGGCGAGGTGCGGTGGCGCAACAGTCTACATCCGGGAGAGCTACCATGGCTGTTGCAGCATTCGGCAC :  3046
B2     : GCACCAGACTCCCCGATGATACTGCGGGCGAGGTACGGTGGCGCAACAGTCTACATCCGGGAGAGCTACCATGGCTGTTGCAGCATTCGGCAC :  3043
Ref    : GCACCAGACTCCCCGATGATACTGCGGGCGAGGTGCGGTGGCGCAACAGTCTACATCCGGGAGAGCTACCATGGCTGTTGCAGCATTCGGCAC :  3043
70-15  : GCACCAGACTCCCCGATGATACTGCGGGCGAGGTGCGGTGGCGCAACAGTCTACATCCGGGAGAGCTACCATGGCTGTTGCAGCATTCGGCAC :  3043

SCAU-2 : AGGGTCAGACTGTTTTCCCCGGGGACAGGCTACATCGCAACCGTGCTTGAGGCGGTCAAACAGCTATTTGAGAGTAACGGGGTTCAAACGGTAG :  3150
NI907  : AGGGTCAGACTGTTTTCCCCGGGGACAGGCTACATCGCAACCGTGCTTGAGGCGGTCAAACAGCTATTTGAGAGTAACGGGGTTCAAACGGTAG :  3150
W97-11 : AGGGTCAGACTGTTTTCCCCGGGGACAGGCTACATCGCAACCGTGCTTGAGGCGGTCAAACAGCTATTTGAGAGTAACGGGGTTCAAACGGTAG :  3150
B71    : AGGGTCAGACTGTCTTCCCGGGAACAGGCTACATCGCAACCACGCTTGAGGCGGTCAAGCAGTTGTTTGATAGCAGCGGGGTGCAAACGGTGG :  3139
B2     : AGGGTCAGACTGTCTTCCCGGGGACGGGCTACATCGCAACCACGCTCGAGGCGGTCAAGCAGTTGTTTGATAGCAGCGGGGTGCAAACGGTGG :  3136
Ref    : AGGGTCAGACTGTCTTCCCGGGGACGGGCTACATCGCAACCACGCTCGAGGCGGTCAAGCAGTTGTTTGATAGCAGCGGGGTGCAAACGGTGG :  3136
70-15  : AGGGTCAGACTGTCTTCCCGGGGACGGGCTACATCGCAACCACGCTCGAGGCGGTCAAGCAGTTGTTTGATAGCAGCGGGGTGCAAACGGTGG :  3136

SCAU-2 : AGCTACGAGATTTTGTCATCGGCAACGCTCTAGTCATCGAGGCAAACGCCGGTGTGGAAACGCTCTTTTCATTAACTGGCATCAATAGCCAGG :  3243
NI907  : AGCTACGAGATTTTGTCATCGGCAACGCTCTAGTCATCGAGGCAAACGCCGGTGTGGAAACGCTCTTTTCATTAACTGGCATCAATAGCCAGG :  3243
W97-11 : AGCTACGAGATTTTGTCATCGGCAACGCTCTAGTCATCGAGGCAAACGCCGGTGTGGAAACGCTCTTTTCATTAACTGGCATCAATAGCCAGG :  3243
B71    : AGATACGAGACATGGTCATCGGCAACGCTCTCGTCATCGAGGCAAACACCGGGGTCGAGACGCTCTTTTCGTTGACCTCCATAAACACCCAGA :  3232
B2     : AGATACGAGACATGGTCATCGGCAACGCTCTCGTCATCGAGGCAAACACCGGGGTCGAGACGCTCTTTTCGTTGACCTCCATAAACACCCAGA :  3229
Ref    : AGATACGAGACATGGTCATCGGCAACGCTCTCGTCATCGAGGCAAACACCGGGGTCGAGACGCTCTTTTCGTTGACCTTCATAAACACCCAGA :  3229
70-15  : AGATACGAGACATGGTCATCGGCAACGCTCTCGTCATCGAGGCAAACACCGGGGTCGAGACGCTCTTTTCGTTGACCTTCATAAACACCCAGA :  3229

SCAU-2 : CAGACCGCATCACCGCCCACTTTTCATTCTCTAGCCAGCAGGGCAACAGCACCAAGCTAGTGGAAAATGCATCTGGAGACCTTACTGTGGTTC :  3336
NI907  : CAGACCGCATCACCGCCCACTTTTCATTCTCTAGCCAGCAGGGCAACAGCACCAAGCTAGTGGAAAATGCATCTGGAGACCTTACTGTGGTTC :  3336
W97-11 : CAGACCGCATCACCGCCCACTTTTCATTCTCTAGCCAGCAGGGCAACAGCACCAAGCTAGTGGAAAATGCATCTGGAGACCTTACTGTGGTTC :  3336
B71    : CGGACCGCATCACCGCCCACTTTTCATTCTGCAGCCAGCAGGGCGGCAGCACCAAGCTGGTGGAGAATGCATCTGGAGACCTGGTGGTGCTTC :  3325
B2     : CGGACCGCATCACCGCCCACTTTTCATTCTGCAGCCAGCAGGGCGGCAGCACCAAGCTGGTGGAGAATGCATCTGGAGACCTGGTGGTGCTTC :  3322
Ref    : CGGACCGCATCACCGCCCACTTTTCATTCTGCAGCCAGCAGGGCGGCAGCACCAAGCTGGTGGAGAATGCATCTGGAGACCTGGTGGTGCTTC :  3322
70-15  : CGGACCGCATCACCGCCCACTTTTCATTCTGCAGCCAGCAGGGCGGCAGCACCAAGCTGGTGGAGAATGCATCTGGAGACCTGGTGGTGCTTC :  3322
```

```
SCAU-2 : TTGGTAAGCCCTCGCAGGATGCACTACCTAAGAACTTCCCTTCAGTTACGCAGATGAAGGATATCGACGAGGCGCGCTTTTATGAGGCCATTG :  3429
NI907  : TTGGTAAGCCCTCGCAGGATGCACTACCTAAGAACTTCCCTTCAGTTACGCAGATGAAGGATATCGACGAGGCGCGCTTTTATGAGGCCATTG :  3429
W97-11 : TTGGTAAGCCCTCGCAGGATGCACTACCTAAGAACTTCCCTTCAGTTACGCAGATGAAGGATATCGACGAGGCGCGCTTTTATGAGGCCATTG :  3429
B71    : TCGGTGAGCCCTCGGAGGATGCGTTGCCCAGGAGTTTCCACCCAGGCACGCAGATGAAGGACATTGACGAGGAGCGCTTCTACGAGGCCATTG :  3418
B2     : TCGGTGAGCCCTCGGAGGATGCGCTGCCCAGGAGTTTCCACCCAGGCACGCAGATGAAGGACATTGACGAGGAGCGCTTCTACGAGGCCATTG :  3415
Ref    : TCGGTGAGCCCTCGGAGGATGCGCTGCCCAGGAGTTTCCACCCAGGCACGCAGATGAAGGACATTGACGAGGAGCGCTTCTACGAGGCCATTG :  3415
70-15  : TCGGTGAGCCCTCGGAGGATGCGCTGCCCAGGAGTTTCCACCCAGGCACGCAGATGAAGGACATTGACGAGGAGCGCTTCTACGAGGCCATTG :  3415

SCAU-2 : ACAAACTTGGCTACGGCTACGAGGGGCCTTTCAGGGCCCTTTCAAGACTGCAACGCCGAATGGGGGCAGCCACTGGTTTTGTGGCGGTTCCTG :  3522
NI907  : ACAAACTTGGCTACGGCTACGAGGGGCCTTTCAGGGCCCTTTCAAGACTGCAACGCCGAATGGGGGCAGCCACTGGTTTTGTGGCGGTTCCTG :  3522
W97-11 : ACAAACTTGGCTACGGCTACGAGGGGCCTTTCAGGGCCCTTTCAAGACTGCAACGCCGAATGGGGGCAGCCACTGGTTTTGTGGCGGTTCCTG :  3522
B71    : ACAAACTCGGCTACGGCTACGAGGGTCCCTTCAGGGCGCTTTCGCAGCTGCAACGCCGGATGGGGGCTGCCACTGGTCTCGTGGCGATCCCGG :  3511
B2     : ACAAACTCGGCTACGGCTACGAGGGTCCCTTCAGGGCGCTTTCGCAGCTGCAACGCCGGATGGGGGCTGCCACTGGTCTCGTGGCGATCCCCG :  3508
Ref    : ACAAACTCGGCTACGGCTACGAGGGTCCCTTCAGGGCGCTTTCGCAGCTGCAACGCCGGATGGGGGCTGCCACTGGTCTCGTGGCGATCCCCG :  3508
70-15  : ACAAACTCGGCTACGGCTACGAGGGTCCCTTCAGGGCGCTTTCGCAGCTGCAACGCCGGATGGGGGCTGCCACTGGTCTCGTGGCGATCCCCG :  3508

SCAU-2 : AAAAGACCAAACACTTTGACCAGATGGTCTTCCACCCTGCAGCCCTCGATGCCATGGTGCAAACAATTCTACTGGCCTACTGCTACCCTGGCG :  3615
NI907  : AAAAGACCAAACACTTTGACCAGATGGTCTTCCACCCTGCAGCCCTCGATGCCATGGTGCAAACAATTCTACTGGCCTACTGCTACCCTGGCG :  3615
W97-11 : AAAAGACCAAACACTTTGACCAGATGGTCTTCCACCCTGCAGCCCTCGATGCCATGGTGCAAACAATTCTACTGGCCTACTGCTACCCTGGCG :  3615
B71    : AAAAGACGAAGCACTTTGACCAGATGGTCCTCCACCCCGCAGCCCTGGACGCCATGGTGCAGACCGTCCTGCTGGCCTACTGCTACCCCGGCG :  3604
B2     : AAAAGACGAAGCACTTTGACCAGATGGTCCTCCACCCCGCAGCCCTGGACGCCATGGTACAGACCGTCCTGCTGGCCTACTGCTACCCTGGCG :  3601
Ref    : AAAAGACGAAGCACTTTGACCAGATGGTCCTCCACCCCGCAGCCCTGGACGCCATGGTACAGACCGTCCTGCTGGCCTACTGCTACCCTGGCG :  3601
70-15  : AAAAGACGAAGCACTTTGACCAGATGGTCCTCCACCCCGCAGCCCTGGACGCCATGGTACAGACCGTCCTGCTGGCCTACTGCTACCCTGGCG :  3601

SCAU-2 : ATACACGTCTCCAGGGCATTTCTCTCCCTACCGGGATTGACTGCATTCGATTCAACTATGGGATGCTCAGTCAAGCGGCAAGACCTGGTTCTC :  3708
NI907  : ATACACGTCTCCAGGGCATTTCTCTCCCTACCGGGATTGACTGCATTCGATTCAACTATGGGATGCTCAGTCAAGCGGCAAGACCTGGTTCTC :  3708
W97-11 : ATACACGTCTCCAGGGCATTTCTCTCCCTACCGGGATTGACTGCATTCGATTCAACTATGGGATGCTCAGTCAAGCGGCAAGACCTGGTTCTC :  3708
B71    : ACACCCGCCTCCAGGGAATCTCCCTCCCCACCGGCATCGACTGCATCCGATTCAACTATGGCATGCTCAGCGAGGCGGCGAGACCCGGCTGTC :  3697
B2     : ACACCCGCCTCCAGGGAATCTCCCTCCCCACCGGCATCGACTGCATCCGATTCAACTATGGCATGCTCAGCGAGGCGGCGAGACCCGGCTGTC :  3694
Ref    : ACACCCGCCTCCAGGGAATCTCCCTCCCCACCGGCATCGACTGCATCCGATTCAACTATGGCATGCTCAGCGAGGCGGCGAGACCCGGCTGTC :  3694
70-15  : ACACCCGCCTCCAGGGAATCTCCCTCCCCACCGGCATCGACTGCATCCGATTCAACTATGGCATGCTCAGCGAGGCGGCGAGACCCGGCTGTC :  3694
```

```
SCAU-2 : AGCTCCCCTTCATGTCCTTTACAGCCTTTGAGGGCGATGATGTCTTGGCTGGGGCCGGAAGTGACGTTGGTGGCGACGTCGACGTCTTTTCCG :  3801
NI907  : AGCTCCCCTTCATGTCCTTTACAGCCTTTGAGGGCGATGATGTCTTGGCTGGGGCCGGAAGTGACGTTGGTGGCGACGTCGACGTCTTTTCCG :  3801
W97-11 : AGCTCCCCTTCATGTCCTTTACAGCCTTTGAGGGCGATGATGTCTTGGCTGGGGCCGGAAGTGACGTTGGTGGCGACGTCGACGTCTTTTCCG :  3801
B71    : AGCTCCCGTTCCTCTCCTGCACTGCTTTCGAAGGCGATGACGTGTTGGGCGGGGTCGGAGGCGACGTGGGCGGCGATGTCGACGTCTTCTCCG :  3790
B2     : AGCTCCCGTTCCTCTCCTGCACTGCTTTCGAAGGCGATGACGTCTTGGGCGGGGTCGGAGGCGACGTGGGCGGCGATGTCGACGTCTTCTCCG :  3787
Ref    : AGCTCCCGTTCCTCTCCTGCACTGCTTTCGAAGGCGATGACGTCTTGGGCGGGGTCGGAGGCGACGTGGGCGGCGATGTCGACGTCTTCTCCG :  3787
70-15  : AGCTCCCGTTCCTCTCCTGCACTGCTTTCGAAGGCGATGACGTCTTGGGCGGGGTCGGAGGCGACGTGGGCGGCGATGTCGACGTCTTCTCCG :  3787

SCAU-2 : AGGACAAGAGCTTTGCACTGGTTCAGCTCCAAGGCCTGCACACAAAGCCACTTTCTCCACCGTCGGCCGCCACCGATCTGCAGATCTTTTCCG :  3894
NI907  : AGGACAAGAGCTTTGCACTGGTTCAGCTCCAAGGCCTGCACACAAAGCCACTTTCTCCACCGTCGGCCGCCACCGATCTGCAGATCTTTTCCG :  3894
W97-11 : AGGACAAGAGCTTTGCACTGGTTCAGCTCCAAGGCCTGCACACAAAGCCACTTTCTCCACCGTCGGCCGCCACCGATCTGCAGATCTTTTCCG :  3894
B71    : AGGACAAGAGGTTCGCGCTGATTCAGCTCCAAGGCCTCCACACAAAGCCGCTGTCCCCACCGTCGGCCGCCACCGACCTGCAAATCTTTTCCG :  3883
B2     : AGGACAAGAGGTTCGCGCTGATTCAGCTCCAAGGCCTCCACACAAAGCCGCTGTCCCCACCGTCGGCCGCCACCGACCTGCAAATCTTTTCCG :  3880
Ref    : AGGACAAGAGGTTCGCGCTGATTCAGCTCCAAGGCCTCCACACAAAGCCGCTGTCCCCACCGTCGGCCGCCACCGACCTGCAAATCTTTTCCG :  3880
70-15  : AGGACAAGAGGTTCGCGCTGATTCAGCTCCAAGGCCTCCACACAAAGCCGCTGTCCCCACCGTCGGCCGCCACCGACCTGCAAATCTTTTCCG :  3880

SCAU-2 : AGATGGAGTGGAAGATTATAATCCCCCGAGGGAGCCGACATAGAGGTCCGGGGTGAGAAGCGAGCCTATGTCGCCGACTTGTTCACATCCATAG :  3987
NI907  : AGATGGAGTGGAAGATTATAATCCCCCGAGGGAGCCGACATAGAGGTCCGGGGTGAGAAGCGAGCCTATGTCGCCGACTTGTTCACATCCATAG :  3987
W97-11 : AGATGGAGTGGAAGATTATAATCCCCCGAGGGAGCCGACATAGAGGTCCGGGGTGAGAAGCGAGCCTATGTCGCCGACTTGTTCACATCCATAG :  3987
B71    : AGATGGAGTGGAAGACGGCGTCGCCTGAGGGGGCCGACATGGAGGTGCGCGGCGAGAAGCGCGCCTACGTCGCCGACCTGTACTCGTCCATGG :  3976
B2     : AGATGGAGTGGAAGACGGCATCGCCCGAGGGGGCCGACATGGAGGTGCGCGGCGAGAAGCGCGCCTACGTCGCCGACCTGTACTCGTCCATGG :  3973
Ref    : AGATGGAGTGGAAGACGGCATCGCCCGAGGGGGCCGACATGGAGGTGCGCGGCGAGAAGCGCGCCTACGTCGCCGACCTGTACTCGTCCATGG :  3973
70-15  : AGATGGAGTGGAAGACGGCATCGCCCGAGGGGGCCGACATGGAGGTGCGCGGCGAGAAGCGCGCCTACGTCGCCGACCTGTACTCGTCCATGG :  3973

SCAU-2 : AGCGCGTAGCCTATTTCTACATGCGTCATGTGGACCGTGAGATTGGCAAAGACCGCTCCCGCCTGGCTGCTCACCATGTTCGGTTTCTCGAGT :  4080
NI907  : AGCGCGTAGCCTATTTCTACATGCGTCATGTGGACCGTGAGATTGGCAAAGACCGCTCCCGCCTGGCTGCTCACCATGTTCGGTTTCTCGAGT :  4080
W97-11 : AGCGCGTAGCCTATTTCTACATGCGTCATGTGGACCGTGAGATTGGCAAAGACCGCTCCCGCCTGGCTGCTCACCATGTTCGGTTTCTCGAGT :  4080
B71    : AGCGCGTGGCCTACTTTTACATGCGCCACGTGGATCGCGAGATTGGCAAGGACCGCTCCGGCCTGGCTCCCCACCAAGTTCGCTTCCTCGAGT :  4069
B2     : AGCGCGTGGCCTACTTTTACATGCGCCACGTGGATCGCGAGATTGGCAAGGACCGCTCCGGCCTGGCTCCCCACCAAGTTCGCTTCCTCGAGT :  4066
Ref    : AGCGCGTGGCCTACTTTTACATGCGCCACGTGGATCGCGAGATTGGCAAGGACCGCTCCGGCCTGGCTCCCCACCAAGTTCGCTTCCTCGAGT :  4066
70-15  : AGCGCGTGGCCTACTTTTACATGCGCCACGTGGATCGCGAGATTGGCAAGGACCGCTCCGGCCTGGCTCCCCACCAAGTTCGCTTCCTCGAGT :  4066
```

```
SCAU-2 : GGGTTGACCACATGTGCGGTCGCGTTGAGCAGGGGACGCTCCCTCACATCAACCGCAAATGGGACTACGATACTCGGGATGATATCCTGAAAA :  4173
NI907  : GGGTTGACCACATGTGCGGTCGCGTTGAGCAGGGGACGCTCCCTCACATCAACCGCAAATGGGACTACGATACTCGGGATGATATCCTGAAAA :  4173
W97-11 : GGGTTGACCACATGTGCGGTCGCGTTGAGCAGGGGACGCTCCCTCACATCAACCGCAAATGGGACTACGATACTCGGGATGATATCCTGAAAA :  4173
B71    : GGGTGGATCACATGTGCGGTCGCGTCGAGGCGGGCACGCTTCCTCACATCAGCCGCAAGTGGGACCACGACACTCGGCAAGACATCCTAAAGA :  4162
B2     : GGGTGGATCACATGTGCGGTCGCGTCGAGGCGGGCACGCTTCCTCACATCAGCCGCAAGTGGGACCACGACACTCGGCAAGACATCCTAAAGA :  4159
Ref    : GGGTGGATCACATGTGCGGTCGCGTCGAGGCGGGCACGCTTCCTCACATCAGCCGCAAGTGGGACCACGACACTCGGCAAGACATCCTAAAGA :  4159
70-15  : GGGTGGATCACATGTGCGGTCGCGTCGAGGCGGGCACGCTTCCTCACATCAGCCGCAAGTGGGACCACGACACTCGGCAAGACATCCTAAAGA :  4159

SCAU-2 : TCATTGCAAAATACCCCGACAGCATAGATCTTGAGTTGATGCATGCGGTGGGAGAGAATCTTTGTTCTGTTTTCAGAGGAGAGATGAATCCGC :  4266
NI907  : TCATTGCAAAATACCCCGACAGCATAGATCTTGAGTTGATGCATGCGGTGGGAGAGAATCTTTGTTCTGTTTTCAGAGGAGAGATGAATCCGC :  4266
W97-11 : TCATTGCAAAATACCCCGACAGCATAGATCTTGAGTTGATGCATGCGGTGGGAGAGAATCTTTGTTCTGTTTTCAGAGGAGAGATGAATCCGC :  4266
B71    : TCATTGCGAAATACCCCGACAGCATCGACCTCGAGCTGATGCACGCGGTGGGGGAGAACCTCTGCTCCGTCTTTAGGGGAGAGATGAATGTGC :  4255
B2     : TCATTGCGAAATACCCCGACAGCATCGACCTCGAGCTGATGCACGCGGTTGGGGAGAATCTCTGCTCCGTCTTTAGGGGGGAGATGAACGCGC :  4252
Ref    : TCATTGCGAAATACCCCGACAGCATCGACCTCGAGCTGATGCACGCGGTCGGGGAGAATCTCTGCTCCGTCTTTAGGGGGGAGATGAACGCGC :  4252
70-15  : TCATTGCGAAATACCCCGACAGCATCGACCTCGAGCTGATGCACGCGGTCGGGGAGAATCTCTGCTCCGTCTTTAGGGGGGAGATGAACGCGC :  4252

SCAU-2 : TGGAGCCCATGGTTAAAAACAATATGCTGAACAGGTTTTACACCGATGCCCTTGGTATGTCACCATATACCGAGGACCTCGCTCGTATGGTGG :  4359
NI907  : TGGAGCCCATGGTCAAAAACAATATGCTGAACAGGTTTTACACCGATGCCCTTGGTATGTCACCATATACCGAGGACCTCGCTCGTATGGTGG :  4359
W97-11 : TGGAGCCCATGGTTAAAAACAATATGCTGAACAGGTTTTACACCGATGCCCTTGGTATGTCACCATATACCGAGGACCTCGCTCGTATGGTGG :  4359
B71    : TGGAGCCCATGGTCAAGAAGAACATGCTCAACCGGTTCTACTCGGACGCCCTCGGCATGTCGCCGTACACCGAGGACCTCGCCCGCATGGTGG :  4348
B2     : TGGAGCCCATGGTCAAGAAGAACATGCTCAACCGGTTCTACTCGGACGCCCTCGGCATGTCGCCGTACACCGAGGACCTCGCCCGCATGGTGG :  4345
Ref    : TGGAGCCCATGGTCAAGAAGAACATGCTCAACCGGTTCTACTCGGACGCCCTCGGCATGTCGCCGTACACCGAGGACCTCGCCCGCATGGTGG :  4345
70-15  : TGGAGCCCATGGTCAAGAAGAACATGCTCAACCGGTTCTACTCGGACGCCCTCGGCATGTCGCCGTACACCGAGGACCTCGCCCGCATGGTGG :  4345

SCAU-2 : AACATATCACGCATCGCTATCCTCACATGAACATACTCGAAGTCGGTGCAGGAACTGGTGGTGCGACCAAGGTTATGCTCCGGAAGCTGAAAG :  4452
NI907  : AACATATCACGCATCGCTATCCTCACATGAACATACTCGAAGTCGGTGCAGGAACTGGTGGTGCGACCAAGGTTATGCTCCGGAAGCTGAAAG :  4452
W97-11 : AACATATCACGCATCGCTATCCTCACATGAACATACTCGAAGTCGGTGCAGGAACTGGTGGTGCGACCAAGGTTATGCTCCGGAAGCTGAAAG :  4452
B71    : GTCACATCACGCACCGCTACCCCCACATGAACATACTCGAGGTCGGTGCGGGAACCGGCGGTGCGACAAAGGTGATGCTCAGGAGGCTCCAGG :  4441
B2     : GTCACATCACGCACCGCTACCCCCACATGAACATACTCGAGGTCGGTGCGGGAACCGGCGGTGCGACAAAGGTGATGCTCAGGAGGCTCCAGG :  4438
Ref    : GTCACATCACGCACCGCTACCCCCACATGAACATACTCGAGGTCGGTGCGGGAACCGGCGGTGCGACAAAGGTGATGCTCAGGAGGCTCCAGG :  4438
70-15  : GTCACATCACGCACCGCTACCCCCACATGAACATACTCGAGGTCGGTGCGGGAACCGGCGGTGCGACAAAGGTGATGCTCAGGAGGCTCCAGG :  4438
```

```
SCAU-2 : ATGCTTTTGCCTCGTATACGTATACCGACATCTCCAGCGGCTTCTTTGCCGACGCTCGTGAGGTGTTCAAGGCGCACGAAAGCAAAATGGTGT :  4545
NI907  : ATGCTTTTGCCTCGTATACGTATACCGACATCTCCAGCGGCTTCTTTGCCGACGCTCGTGAGGTGTTCAAGGCGCACGAAAGCAAAATGGTGT :  4545
W97-11 : ATGCTTTTGCCTCGTATACGTATACCGACATCTCCAGCGGCTTCTTTGCCGACGCTCGTGAGGTGTTCAAGGCGCACGAAAGCAAAATGGTGT :  4545
B71    : ACGCCTTTGCCTCGTACACGTACACGGACATCTCGAGCGGCTTTTTTGCCGACGCTCGCCAGGTGTTCAAGGCGCACGAGAGCAAGATGTTTGT :  4534
B2     : ACGCCTTTGCCTCGTACACGTACACGGACATCTCGAGCGGCTTTTTTGCCGACGCTCGTCAGGTGTTCAAGGCGCACGAGAGCAAGATGTTTGT :  4531
Ref    : ACGCCTTTGCCTCGTACACGTACACGGACATCTCGAGCGGCTTTTTTGCCGACGCTCGTCAGGTGTTCAAGGCGCACGAGAGCAAGATGTTTGT :  4531
70-15  : ACGCCTTTGCCTCGTACACGTACACGGACATCTCGAGCGGCTTTTTTGCCGACGCTCGTCAGGTGTTCAAGGCGCACGAGAGCAAGATGTTTGT :  4531

SCAU-2 : TTAAAACTCTGGATATAGAGAAGGAGATCGCCGATCAGGGTTACGAGGAGAACTCGTTCGACCTTGTTATTGCCAACCTCGTCGTGCACGCGA :  4638
NI907  : TTAAAACTCTGGATATAGAGAAGGAGATCGCCGATCAGGGTTACGAGGAGAACTCGTTCGACCTTGTTATTGCCAACCTCGTCGTGCACGCGA :  4638
W97-11 : TTAAAACTCTGGATATAGAGAAGGAGATCGCCGATCAGGGTTACGAGGAGAACTCGTTCGACCTTGTTATTGCCAACCTCGTCGTGCACGCGA :  4638
B71    : TCAAGACGCTGGATATCGAAAAGGACATTGTCGACCAGGGCTACGAGGAGAACTCGTTCGACCTGGTCATTGCCAACCTGGTGGTGCACGCCA :  4627
B2     : TCAAGACGCTGGATATCGAAAAGGACATTGTCGACCAGGGCTACGAGGAGAACTCGTTCGACCTGGTCATTGCCAACTTGGTGGTGCACGCCA :  4624
Ref    : TCAAGACGCTGGATATCGAAAAGGACATTGTCGACCAGGGCTACGAGGAGAACTCGTTCGACCTGGTCATTGCCAACTTGGTGGTGCACGCCA :  4624
70-15  : TCAAGACGCTGGATATCGAAAAGGACATTGTCGACCAGGGCTACGAGGAGAACTCGTTCGACCTGGTCATTGCCAACTTGGTGGTGCACGCCA :  4624

SCAU-2 : CAGCCGACCTGGACGAAACCATGGCCCGCCTCCGTCGCCTCGTCAAGCCAGGGGGTATCTTGTACTCCTAGAAATCACCAACAACGACCCTC :  4731
NI907  : CAGCCGACCTGGACGAAACCATGGCCCGCCTCCGTCGCCTCGTCAAGCCAGGGGGTATCTTGTACTCCTAGAAATCACCAACAACGACCCTC :  4731
W97-11 : CAGCCGACCTGGACGAAACCATGGCCCGCCTCCGTCGCCTCGTCAAGCCAGGGGGTATCTTGTACTCCTAGAAATCACCAACAACGACCCTC :  4731
B71    : CGGCCGACCTGGACGCCACTATGGGCCGTCTCCGTCGCCTCGTTAAGCCCGGTGGTCATCTCGTACTCTTGGAAATCACCACCAACGACCCTC :  4720
B2     : CGGCCGACCTGGACGCCACCATGGGCCGCCTCCGTCGCCTCGTCAAGCCCGGTGGTCATCTCGTACTCTTGGAAATCACCACCAACGACCCCC :  4717
Ref    : CGGCCGACCTGGACGCCACCATGGGCCGCCTCCGTCGCCTCGTCAAGCCCGGTGGTCATCTCGTACTCTTGGAAATCACCACCAACGACCCCC :  4717
70-15  : CGGCCGACCTGGACGCCACCATGGGCCGCCTCCGTCGCCTCGTCAAGCCCGGTGGTCATCTCGTACTCTTGGAAATCACCACCAACGACCCCC :  4717

SCAU-2 : TCAGGTTTGGATTCATATTCGGGCCACTTCCAGGCTGGTGGCTTGGAGGTGAAGACGGACGTGTCCATTCTCCATGTGTCGAGGTCGAATGGT :  4824
NI907  : TCAGGTTTGGATTCATATTCGGGCCACTTCCAGGCTGGTGGCTTGGAGGTGAAGACGGACGTGTCCATTCTCCATGTGTCGAGGTCGAATGGT :  4824
W97-11 : TCAGGTTTGGATTCATATTCGGGCCACTTCCAGGCTGGTGGCTTGGAGGTGAAGACGGACGTGTCCATTCTCCATGTGTCGAGGTCGAATGGT :  4824
B71    : TGAGGTTCGGGTTCATCTTTGGACCCCTCCCGGGCTGGTGGCTTGGAGGCGAAGACGGACGCGTCCATTCCCCCTGCGTCGACGTCGAATGGT :  4813
B2     : TGAGGTTCGGGTTCATCTTTGGACCACTACCGGGCTGGTGGCTTGGAGGCGAAGACGGACGCGTCCATTCCCCGTGTGTCGACGTCGAATGGT :  4810
Ref    : TGAGGTTCGGGTTCATCTTTGGACCACTACCGGGCTGGTGGCTTGGAGGCGAAGACGGACGTGTCCATTCCCCGTGTGTCGACGTCGAATGGT :  4810
70-15  : TGAGGTTCGGGTTCATCTTTGGACCACTACCGGGCTGGTGGCTTGGAGGCGAAGACGGACGTGTCCATTCCCCGTGTGTCGACGTCGAATGGT :  4810
```

```
SCAU-2 : GGGATCGTGTTATGAAACGGTCTGGTTTCTCCGGTGCAGAGATCGTTACGCCGCACCATTCCTTGGGCCCACTTTCAGTGATAATGACACAAG :  4917
NI907  : GGGATCGTGTTATGAAACGGTCTGGTTTCTCCGGTGCAGAGATCGTTACGCCGCACCATTCCTTGGGCCCACTTTCAGTGATAATGACACAAG :  4917
W97-11 : GGGATCGTGTTATGAAACGGTCTGGTTTCTCCGGTGCAGAGATCGTTACGCCGCACCATTCCTTGGGCCCACTTTCAGTGATAATGACACAAG :  4917
B71    : GGGATCGGGTCATGAAACGGAATGGTTTCTCCGGTGCAGACATCGTCACGCCGCACCACACCCTAGGCCCACTCTCGGTGATTATGACACAAG :  4906
B2     : GGGATCGGGTCATGAAACGGAATGGTTTCTCCGGTGCAGACATCGTCACGCCGCACCACACCCTGGGCCCACTCTCGGTGATTATGACACAGG :  4903
Ref    : GGGATCGGGTCATGAAACGGAATGGTTTCTCCGGTGCAGACATCGTCACGCCGCACCACACCCTGGGCCCACTCTCGGTGATTATGACACAGG :  4903
70-15  : GGGATCGGGTCATGAAACGGAATGGTTTCTCCGGTGCAGACATCGTCACGCCGCACCACACCCTGGGCCCACTCTCGGTGATTATGACACAGG :  4903

SCAU-2 : CTGTCGACGACCGTGTGAGACTCCTCAAGGAGCCAACTACCGCTGATTACAAAGAATTCACCATCGATCCCGAACGATTGACCATAGTCGGAG :  5010
NI907  : CTGTCGACGACCGTGTGAGACTCCTCAAGGAGCCAACTACCGCTGATTACAAAGAATTCACCATCGATCCCGAACGATTGACCATAGTCGGAG :  5010
W97-11 : CTGTCGACGACCGTGTGAGACTCCTCAAGGAGCCAACTACCGCTGATTACAAAGAATTCACCATCGATCCCGAACGATTGACCATAGTCGGAG :  5010
B71    : CCGTCGACAACCGCGTGCAACTCCTCAGGCAGCCGACTAGTGCCGACTTTGGAGATTTCGCCATCGATCCCGAGAGATTAACCATAGTGGGAG :  4999
B2     : CCGTCGACCACCGCGTGCAACTCCTCAGGCAGCCGACCAGTGCCGACTTTGGAGATTTCACCATCGATCCCGAGAGGTTGACCATAGTCGGAG :  4996
Ref    : CCGTCGACCACCGCGTGCAACTCCTCAGGCAGCCGACCAGTGCCGACTTTGGAGATTTCACCATCGATCCCGAGAGGTTGACCATAGTCGGAG :  4996
70-15  : CCGTCGACCACCGCGTGCAACTCCTCAGGCAGCCGACCAGTGCCGACTTTGGAGATTTCACCATCGATCCCGAGAGGTTGACCATAGTCGGAG :  4996

SCAU-2 : GTGCCTCACAGTTGGCAAAGGGCTTGGAGCAGCTGTTGAAATCACACTATCAGACCGTCACATGGATACCCAACCTGGAGGATGTCTCATCGC :  5103
NI907  : GTGCCTCACAGTTGGCAAAGGGCTTGGAGCAGCTGTTGAAATCACACTATCAGACCGTCACATGGATACCCAACCTGGAGGATGTCTCATCGC :  5103
W97-11 : GTGCCTCACAGTTGGCAAAGGGCTTGGAGCAGCTGTTGAAATCACACTATCAGACCGTCACATGGATACCCAACCTGGAGGATGTCTCATCGC :  5103
B71    : GCGTCAAGCCGTTGGCAGAGGGCTTGGAGCAGCTGCTCAAGCCACGCTATCAGACCGTCACATGGATACCCACCTTGGAGGAGGTGTCGTCGC :  5092
B2     : GCGTCAAGCCGTTGGCAGAGGGCTTGGAGCAGCTGCTCAAGCCACGCTACCAGAGCGTCGCATGGATACCCACTTTGGAGGAGGTGTCGTCGC :  5089
Ref    : GCGTCAAGCCGTTGGCAGAGGGCTTGGAGCAGCTGCTCAAGCCACGCTACCAGAGCGTCGCATGGATACCCACTTTGGAGGAGGTGTCGTCGC :  5089
70-15  : GCGTCAAGCCGTTGGCAGAGGGCTTGGAGCAGCTGCTCAAGCCACGCTACCAGAGCGTCGCATGGATACCCACTTTGGAGGAGGTGTCGTCGC :  5089

SCAU-2 : AAAGTCTACCCGTCATGGGAAGCGTGCTGTCTCTCGTTGAGCTTGACGAGCCCTTGTTCAAAGACATGACGGTTCAGACGCTCGAGGGCTTCA :  5196
NI907  : AAAGTCTACCCGTCATGGGAAGCGTGCTGTCTCTCGTTGAGCTTGACGAGCCCTTGTTCAAAGACATGACGGTTCAGACGCTCGAGGGCTTCA :  5196
W97-11 : AAAGTCTACCCGTCATGGGAAGCGTGCTGTCTCTCGTTGAGCTTGACGAGCCCTTGTTCAAAGACATGACGGTTCAGACGCTCGAGGGCTTCA :  5196
B71    : AGAGCCTCCCCGTCATGGGAAGCGTGCTGTCCCTCGTGGAGCTCGACGAGCCCCTGTTCAAAGACATGACGGCCCAGACGCTCGAGGGCTTCA :  5185
B2     : ACAGCCTCCCGGTCATGGGAAGCGTGCTGTCCCTCGTGGAGCTCGACGAGCCCCTGTTCAAAGACATGACGGCCCAGACGCTCGAGGGCTTCA :  5182
Ref    : ACAGCCTCCCGGTCATGGGAAGCGTGCTGTCCCTCGTGGAGCTCGACGAGCCCCTGTTCAAAGACATGACGGCCCAGACGCTCGAGGGCTTCA :  5182
70-15  : ACAGCCTCCCGGTCATGGGAAGCGTGCTGTCCCTCGTGGAGCTCGACGAGCCCCTGTTCAAAGACATGACGGCCCAGACGCTCGAGGGCTTCA :  5182
```

```
SCAU-2 : AGCTTGTGTTTCAACAATCCAGAAGCGTTTACTGGATCACCTGCGGAGCTTCGGGTGCAAACCCCTACTCCAACATGGCTGCCGGCGTAGCAA :  5289
NI907  : AGCTTGTGTTTCAACAATCCAGAAGCGTTTACTGGATCACCTGCGGAGCTTCGGGTGCAAACCCCTACTCCAACATGGCTGCCGGCGTAGCAA :  5289
W97-11 : AGCTTGTGTTTCAACAATCCAGAAGCGTTTACTGGATCACCTGCGGAGCTTCGGGTGCAAACCCCTACTCCAACATGGCTGCCGGCGTAGCAA :  5289
B71    : AGCTCGTGTTTCAGCAGTCCAGGAGCGTTTATTGGATCACCTGCGGAGCCTCGGGGGCGAACCCGTACTCCAACATGGCTGCCGGTGTGGCGA :  5278
B2     : AGTTCGTGTTTCAGCAGTCCAGGAGCGTTTATTGGATCACCTGCGGAGCCTCGGGGGCGAACCCGTACTCCAACATGGCCGCCGGTGTGGCGA :  5275
Ref    : AGTTCGTGTTTCAGCAGTCCAGGAGCGTTTATTGGATCACCTGCGGAGCCTCGGGGGCGAACCCGTACTCCAACATGGCCGCCGGTGTGGCGA :  5275
70-15  : AGTTCGTGTTTCAGCAGTCCAGGAGCGTTTATTGGATCACCTGCGGAGCCTCGGGGGCGAACCCGTACTCCAACATGGCCGCCGGTGTGGCGA :  5275

SCAU-2 : GGACGGTTGGTCTCGAGATGCGGCACCTGCGCCTTGGATTTCTTGATTTTGAAAACTCCCAAGATGCCACCGTTCACAACTTGTCCGAGAGCT :  5382
NI907  : GGACGGTTGGTCTCGAGATGCGGCACCTGCGCCTTGGATTTCTTGATTTTGAAAACTCCCAAGATGCCACCGTTCACAACTTGTCCGAGAGCT :  5382
W97-11 : GGACGGTTGGTCTCGAGATGCGGCACCTGCGCCTTGGATTTCTTGATTTTGAAAACTCCCAAGATGCCACCGTTCACAACTTGTCCGAGAGCT :  5382
B71    : GGACCGTCGCGCTCGAGATGCGACACCTGCGCCTTGGATTCCTAGACTTTGAAGACGCCAAAGATGCCACCGTTCAGAGGCTGGCTGACAGGT :  5371
B2     : GGACCGTCGCGCTCGAGATGCGACACCTGCGCCTTGGATTCCTAGACTTTGAAGACGCCAAAGATGCCACCGTTCAGAGGCTGGCCGACAGGT :  5368
Ref    : GGACCGTCGCGCTCGAGATGCGACACCTGCGCCTTGGATTCCTAGACTTTGAAGACGCCAAAGATGCCACCGTTCAGAGGCTGGCCGACAGGT :  5368
70-15  : GGACCGTCGCGCTCGAGATGCGACACCTGCGCCTTGGATTCCTAGACTTTGAAGACGCCAAAGATGCCACCGTTCAGAGGCTGGCCGACAGGT :  5368

SCAU-2 : TTCTGCAGTTCGAAATTCTGGGCACCTTGGAACAGCAAGGGAAGCTGGATCGCCTCACATGGTACCAAGAGCCTGAATTGAGATTCGACGGAA :  5475
NI907  : TTCTGCAGTTCGAAATTCTGGGCACCTTGGAACAGCAAGGGAAGCTGGATCGCCTCACATGGTACCAAGAGCCTGAATTGAGATTCGACGGAA :  5475
W97-11 : TTCTGCAGTTCGAAATTCTGGGCACCTTGGAACAGCAAGGGAAGCTGGATCGCCTCACATGGTACCAAGAGCCTGAATTGAGATTCGACGGAA :  5475
B71    : TCCTCGAGTTTGAAATCCTGGGCACCTTGGAGCAGCAAGGCAAGCTGGATCGCCTGACGTGGTACCAGGAGCCGGAGCTGAGGTTCGACGGAA :  5464
B2     : TCCTCGAGTTTGAAATCCTGGGCACCTTGGAGCAGCAAGGCAAGCTGGATCACCTGACGTGGTACCAGGAGCCGGAGCTGAGGTTCGACGGAA :  5461
Ref    : TCCTCGAGTTTGAAATCCTGGGCACCTTGGAGCAGCAAGGCAAGCTGGATCACCTGACGTGGTACCAGGAGCCGGAGCTGAGGTTCGACGGAA :  5461
70-15  : TCCTCGAGTTTGAAATCCTGGGCACCTTGGAGCAGCAAGGCAAGCTGGATCACCTGACGTGGTACCAGGAGCCGGAGCTGAGGTTCGACGGAA :  5461

SCAU-2 : GCAATTTCCTCGTTCCCCGTATCAGACTGAGCAAAGACCGCAATGCTCGATACAACTCGCGCAGGCGGCAGTTGAGGAAGAATGTTAATCCTC :  5568
NI907  : GCAATTTCCTCGTTCCCCGTATCAGACTGAGCAAAGACCGCAATGCTCGATACAACTCGCGCAGGCGGCAGTTGAGGAAGAATGTTAATCCTC :  5568
W97-11 : GCAATTTCCTCGTTCCCCGTATCAGACTGAGCAAAGACCGCAATGCTCGATACAACTCGCGCAGGCGGCAGTTGAGGAAGAATGTTAATCCTC :  5568
B71    : AGAACCTTCTCGTTCCCCGAATGAAACTGAGCAAAGACCGCAATGGTCGATACAACTCGCGCAGGCGGCAATTGACCAAGAATGTCAATCCCC :  5557
B2     : AGAACCTTCTCGTTCCTCGAATGAAACTGAGCAAAGACCGCAATGGTCGATACAACTCGCGCAGGCGGCAATTGACCAAGAATGTCAATCCCC :  5554
Ref    : AGAACCTTCTCGTTCCTCGAATGAAACTGAGCAAAGACCGCAATGGTCGATACAACTCGCGCAGGCGGCAATTGACCAAGAATGTCAATCCCC :  5554
70-15  : AGAACCTTCTCGTTCCTCGAATGAAACTGAGCAAAGACCGCAATGGTCGATACAACTCGCGCAGGCGGCAATTGACCAAGAATGTCAATCCCC :  5554
```

```
SCAU-2 : GTGAGGTCTCGGTTTCTTTGGTTCCTAGTGGAAAGG------GCTTTGTTCTCGAAGAGTCTCTACACACCTCGTTGTCATCCACTAAGCATG :  5655
NI907  : GTGAGGTCTCGGTTTCTTTGGTTCCTAGTGGAAAGG------GCTTTGTTCTCGAAGAGTCTCTACACACCTCGTTGTCATCCACTAAGCATG :  5655
W97-11 : GTGAGGTCTCGGTTTCTTTGGTTCCTAGTGGAAAGG------GCTTTGTTCTCGAAGAGTCTCTACACACCTCGTTGTCATCCACTAAGCATG :  5655
B71    : GCGAGGTTCCGGTTTCTTTGGTCCCTACTACTAGCGGAAAGGACTTTGTTCTCAAGGAGTCTCTGT---CGTCGTCCTCCACCAAGCACGGGG :  5647
B2     : GCGAGGTTCCGGTTTCTTTGGTCCCTACTACTAGCGGAAAGGACTTTGTTCTCAAGGAGTCTCTGT---CGTCGTCCTCCACCAAGCACGGGG :  5644
Ref    : GCGAGGTTCCGGTTTCTTTGGTCCCTACTACTAGCGGAAAGGACTTTGTTCTCAAGGAGTCTCTGT---CGTCGTCCTCCACCAAGCACGGGG :  5644
70-15  : GCGAGGTTCCGGTTTCTTTGGTCCCTACTACTAGCGGAAAGGACTTTGTTCTCAAGGAGTCTCTGT---CGTCGTCCTCCACCAAGCACGGGG :  5644

SCAU-2 : GCCAGGATATGGTGACTTTAAGGGTCCATTATGCCATGCATCGGTCGGTGAGACTGGAATCCTCGGATTACCTTTTCCTCGTTCTGGGCACCG :  5748
NI907  : GCCAGGATATGGTGACTTTAAGGGTCCATTATGCCATGCATCGGTCGGTGAGACTGGAATCCTCGGATTACCTTTTCCTCGTTCTGGGCACCG :  5748
W97-11 : GCCAGGATATGGTGACTTTAAGGGTCCATTATGCCATGCATCGGTCGGTGAGACTGGAATCCTCGGATTACCTTTTCCTCGTTCTGGGCACCG :  5748
B71    : CCCAGGACACGGTGTCGTTGAGAGTCCATTACGCCAGTCAACGGTCGCTGAGGCTGGAGTCCTCAGACTACCTCTTCCTCGTCCTGGGCACCA :  5740
B2     : CCCAGGACACGGTGTCGTTGAGAGTCCATTACGCCAGTCAACGGTCGCTGAGGCTGGAGTCCTCAGACTACCTCTTCCTCGTCCTGGGCACCA :  5737
Ref    : CCCAGGACACGGTGTCGTTGAGAGTCCATTACGCCAGTCAACGGTCGCTGAGGCTGGAGTCCTCAGACTACCTCTTCCTCGTCCTGGGCACCA :  5737
70-15  : CCCAGGACACGGTGTCGTTGAGAGTCCATTACGCCAGTCAACGGTCGCTGAGGCTGGAGTCCTCAGACTACCTCTTCCTCGTCCTGGGCACCA :  5737

SCAU-2 : ACATCTCTTCGGGCGAAGCCATGTTTGCCCTGGCAGATTCGCAAAAGTCGATTGTGCAGGTAGACCGGCAGTGGACAGCACCATACGTTGGCA :  5841
NI907  : ACATCTCTTCGGGCGAAGCCATGTTTGCCCTGGCAGATTCGCAAAAGTCGATTGTGCAGGTAGACCGGCAGTGGACAGCACCATACGTTGGCA :  5841
W97-11 : ACATCTCTTCGGGCGAAGCCATGTTTGCCCTGGCAGATTCGCAAAAGTCGATTGTGCAGGTAGACCGGCAGTGGACAGCACCATACGTTGGCA :  5841
B71    : ACCTCTCCTCGGGCGAAGCCATGTTTGCCCTGGCAGACTCCAACAGGTCGATTGTGCACGTAGACCGGCAGTGGACGACGTCGTACCTTGGCA :  5833
B2     : ACCTCTCTTCGGGCGAAGCCATGTTTGCCCTGGCAGACTCCAACAGGTCGATTGTGCACGTAGACCGGCAGTGGACGACGTCGTACCTTGGCA :  5830
Ref    : ACCTCTCTTCGGGCGAAGCCATGTTTGCCCTGGCAGACTCCAACAGGTCGATTGTGCACGTAGACCGGCAGTGGACGACGTCGTACCTTGGCA :  5830
70-15  : ACCTCTCTTCGGGCGAAGCCATGTTTGCCCTGGCAGACTCCAACAGGTCGATTGTGCACGTAGACCGGCAGTGGACGACGTCGTACCTTGGCA :  5830

SCAU-2 : ACCTAGCTGATGGCAAGCACGCGCTGGCTGGTCTATACACGCAGATTATTGCGTCAACGGTGGTGGCGGTCCTTTCCTCGGGCGACTCGCTCG :  5934
NI907  : ACCTAGCTGATGGCAAGCACGCGCTGGCTGGTCTATACACGCAGATTATTGCGTCAACGGTGGTGGCGGTCCTTTCCTCGGGCGACTCGCTCG :  5934
W97-11 : ACCTAGCTGATGGCAAGCACGCGCTGGCTGGTCTATACACGCAGATTATTGCGTCAACGGTGGTGGCGGTCCTTTCCTCGGGCGACTCGCTCG :  5934
B71    : ACCTAGACCACGGCAGGCACGCACTGGCTGGTCTGTACACGCAGATCATGGCGTCGACGGTGGTGGCGGGGCTTTCCGCGGGCGACTCCCTCG :  5926
B2     : ACCTGGATCACGGCAGGCACGCACTGGCTGGTCTGTACACGCAGATCATGGCGTCGACGGTGGTGGCGGGGCTTTCCGCGGGCGACTCCCTCG :  5923
Ref    : ACCTGGATCACGGCAGGCACGCACTGGCTGATCTGTACACGCAGATCATGGCGTCGACGGTGGTGGCGGGGCTTTCCGCGGGCGACTCCCTCG :  5923
70-15  : ACCTGGATCACGGCAGGCACGCACTGGCTGATCTGTACACGCAGATCATGGCGTCGACGGTGGTGGCGGGGCTTTCCGCGGGCGACTCCCTCG :  5923
```

```
SCAU-2 : TCGTACTCGACGCAGAAACGTCGCTCTCGCTAGCCCTCTCGGCTCGCTGTGATTCAAAGGGTGTACGACTCACGCTACTCAGCACCACCAGTC :  6027
NI907  : TCGTACTCGACGCAGAAACGTCGCTCTCGCTAGCCCTCTCGGCTCGCTGTGATTCAAAGGGTGTACGACTCACGCTACTCAGCACCACCAGTC :  6027
W97-11 : TCGTACTCGACGCAGAAACGTCGCTCTCGCTAGCCCTCTCGGCTCGCTGTGATTCAAAGGGTGTACGACTCACGCTACTCAGCACCACCAGTC :  6027
B71    : TGGTGCTCGACGCGGAAACGCCCCTCTCGCTGGCCCTCTCGGCTCGCTGCGCCGCAAAGGGGGTGCGACTCACCCTGCTCAGCACCACCACCG :  6019
B2     : TGGTGCTCGACGCGGAAACGCCCCTCTCGCAGGCCCTCTCGGCTCGCTGCGCCGCAAAGGGGGTGCGACTCACCCTGCTCAGCACCACCACCG :  6016
Ref    : TGGTGCTCGACGCGGAAACGCCCCTCTCGCAGGCCCTCTCGGCTCGCTGCGCCGCAAAGGGGGTGCGACTCACCCTGCTCAGCACCACCACCG :  6016
70-15  : TGGTGCTCGACGCGGAAACGCCCCTCTCGCAGGCCCTCTCGGCTCGCTGCGCCGCAAAGGGGGTGCGACTCACCCTGCTCAGCACCACCACCG :  6016

SCAU-2 : CCGACTCGGACTCTAG---------TGCTGCGAACAAGACAGTTCGTATCCACCCCTTCGAATCTCGCAGATCCATCGAGTCAAAACTGCCGA :  6111
NI907  : CCGACTCGGACTCTAG---------TGCTGCGAACAAGACAGTTCGTATCCACCCCTTCGAATCTCGCAGATCCATCGAGTCAAAACTGCCGA :  6111
W97-11 : CCGACTCGGACTCTAG---------TGCTGCGAACAAGACAGTTCGTATCCACCCCTTCGAATCTCGCAGATCCATCGAGTCAAAACTGCCGA :  6111
B71    : CCACCTCGCACTCGGAGGCGGACGGTACCAACAAGACGAACGTCCGTATCCACCCCTTGGAGTCTCGCCGATCCATCGAGTCGAAACTGCCGT :  6112
B2     : CCACCTCGCACTCGGAGGCGGACGGTACCAACAAGACGAACGTCCGTATCCACCCCTTGGAGTCTCGCCGATCCATCGAGTCGAAACTGCCGT :  6109
Ref    : CCACCTCGCACTCGGAGGCGGACGGTACCAACAAGACGAACGTCCGTATCCACCCCTTGGAGTCTCGCCGATCCATCGAGTCGAAACTGCCGT :  6109
70-15  : CCACCTCGCACTCGGAGGCGGACGGTACCAACAAGACGAACGTCCGTATCCACCCCTTGGAGTCTCGCCGATCCATCGAGTCGAAACTGCCGT :  6109

SCAU-2 : CGAGCACCACTTGCTTCCTCAACCTTTCCAGTAGCAAGGAAAACGTGACCGCGGATGTCATCAACAGCTACATTCCAACGCAGTGCCGGGTGG :  6204
NI907  : CGAGCACCACTTGCTTCCTCAACCTTTCCAGTAGCAAGGAAAACGTGACCGCGGATGTCATCAACAGCTACATTCCAACGCAGTGCCGGGTGG :  6204
W97-11 : CGAGCACCACTTGCTTCCTCAACCTTTCCAGTAGCAAGGAAAACGTGACCGCGGATGTCATCAACAGCTACATTCCAACGCAGTGCCGGGTGG :  6204
B71    : CCAACGCCACCTGCTTCCTCGACCTCTCGACCAGCAACGGCAGCGAGGCCGCGGCCGTCATCAACAGCTACATCCCGGCGCAGTGCCGCGTGG :  6205
B2     : CCAACGCCACCTGCTTCCTCGACCTCTCGACCAGCAACGGCAGCGAGGCCGCGGCCGTCATCAACAGCTACATCCCGGCGCAGTGCCGCGTGG :  6202
Ref    : CCAACGCCACCTGCTTCCTCGACCTCTCGACCAACAACGGCAGCGAGGCCGCGGCCGTCATCAACAGCTACATCCCGGCGCAGTGCCGCGTGG :  6202
70-15  : CCAACGCCACCTGCTTCCTCGACCTCTCGACCAACAACGGCAGCGAGGCCGCGGCCGTCATCAACAGCTACATCCCGGCGCAGTGCCGCGTGG :  6202

SCAU-2 : AGACCCGTGATTCACTGACTGCGCTTGTAGGCCAAATCACCAGGTCCACCTCCATGGG---TCTGACTTCTGCAGTCGCAGATATCTTGCGTA :  6294
NI907  : AGACCCGTGATTCACTGACTGCGCTTGTAGGCCAAATCACCAGGTCCACCTCCATGGG---TCTGACTTCTGCAGTCGCAGATATCTTGCGTA :  6294
W97-11 : AGACCCGTGATTCACTGACTGCGCTTGTAGGCCAAATCACCAGGTCCACCTCCATGGG---TCTGACTTCTGCAGTCGCAGATATCTTGCGTA :  6294
B71    : AGACGCGCGACACACTGACGGCGACCGCAGGCCAGGTCACCAGGTCCACCTCCACCGGCGGCCTGGGCCCTGCGGTCGGCGACGTCTTGCCTG :  6298
B2     : AGACGCGCGACACACTGACGGCGACCGCATGCCAGGTCACCAGGTCCACCTCCACCGGCGGCCTGGGCCCTGCGGTCGGCGACGTCTTGCCTG :  6295
Ref    : AGACGCGCGACACACTGACGGCGACCGCATGCCAGGTCACCAGGTCCACCTCCACCGGCGGCCTGGGCCCTGCGGTCGGCGACGTCTTGCCTG :  6295
70-15  : AGACGCGCGACACACTGACGGCGACCGCATGCCAGGTCACCAGGTCCACCTCCACCGGCGGCCTGGGCCCTGCGGTCGGCGACGTCTTGCCTG :  6295
```

```
SCAU-2 : CTTGCTGGGCTAACGTTCAGGCTGTGAGACGAGACTTGACACCTTTTTCGGGCGCTGTCTTTACTCCCACCGAGCTCACTGCTACAGTTG--- : 6384
NI907  : CTTGCTGGGCTAACGTTCAGGCTGTGAGACGAGACTTGACACCTTTTTCGGGCGCTGTCTTTACTCCCACCGAGCTCACTGCTACAGTTG--- : 6384
W97-11 : CTTGCTGGGCTAACGTTCAGGCTGTGAGACGAGACTTGACACCTTTTTCGGGCGCTGTCTTTACTCCCACCGAGCTCACTGCTACAGTTG--- : 6384
B71    : CCTGCTGGGCCAACGTCGAGGCCGCGGGGCGAGCCTTGTCCTTCTTCTCGGCCGCCGTCGTCACTCCCACTGAGCTCACCGCCGCGGCCGGGA : 6391
B2     : CCTGCTGGGCCAACGTCGAGGCCGCGGGGCGAGACTTGTCCTTCTTCTCGGCCGCCGTCGTCACTCCCACCGAGCTCACCGCCGCGGCCGGGA : 6388
Ref    : CCTGCTGGGCCAACGTCGAGGCCGCGGGGCGAGACTTGTCCTTCTTCTCGGCCGCCGTCGTCACTCCCACCGAGCTCACCGCCGCGGCCGGGA : 6388
70-15  : CCTGCTGGGCCAACGTCGAGGCCGCGGGGCGAGACTTGTCCTTCTTCTCGGCCGCCGTCGTCACTCCCACCGAGCTCACCGCCGCGGCCGGGA : 6388

SCAU-2 : ------GGAAGATGAGTCCCAAAGTTGGCAATGATGCCCTTTCGGTTATTACTGACTGGACTGCAGAAGAAGAACTAGGCGTGTTAATCCAGC : 6471
NI907  : ------GGAAGATGAGTCCCAAAGTTGGCAATGATGCCCTTTCGGTTATTACTGACTGGACTGCAGAAGAAGAACTAGGCGTGTTAATCCAGC : 6471
W97-11 : ------GGAAGATGAGTCCCAAAGTTGGCAATGATGCCCTTTCGGTTATTACTGACTGGACTGCAGAAGAAGAACTAGGCGTGTTAATCCAGC : 6471
B71    : ACGGGAAGACGAGCGCCCCCAGAGTTGGCGATGATGCCCTTTTGCTTATTACGGACTGGACCGCCGAAGCAGAGGTCGGGGTGCTGGTGCAGC : 6484
B2     : ACGGGAAGACGAGCGCCCCCAGAGTTGGCGATGATGCCCTTTTGCTTATCACGGACTGGACCGCCGAAGCAGAGGTCGGTGTGCTGGTGCAAC : 6481
Ref    : ACGGGAAGACGAGCGCCCCCAGAGTTGGCGATGATGCCCTTTTGCTTATCACGGACTGGACCGCCGAAGCAGAGGTCGGTGTGCTGGTGCAAC : 6481
70-15  : ACGGGAAGACGAGCGCCCCCAGAGTTGGCGATGATGCCCTTTTGCTTATCACGGACTGGACCGCCGAAGCAGAGGTCGGTGTGCTGGTGCAAC : 6481

SCAU-2 : CTGCCGACTCCATGGTAAGATTCAAACAGGACAAGACCTATTGGTTGGTTGGTCTCACCGGAGGGCTTGCTCTCTCACTGTGTCGTTGGATGG : 6564
NI907  : CTGCCGACTCCATGGTAAGATTCAAACAGGACAAGACCTATTGGTTGGTTGGTCTCACCGGAGGGCTTGCTCTCTCACTGTGTCGTTGGATGG : 6564
W97-11 : CTGCCGACTCCATGGTAAGATTCAAACAGGACAAGACCTATTGGTTGGTTGGTCTCACCGGAGGGCTTGCTCTCTCACTGTGTCGTTGGATGG : 6564
B71    : CTGCCGACTCCATGGTAAGATTCAAACAGGACAAGACCTATTGGTTGGTTGGCCTCACCGGAGGCCTTGCCCTCTCGCTCTGCCGTTGGATGG : 6577
B2     : CTGCCGACTCCATGGTGAGATTCAGACAGGACAAGACTTATTGGTTAGTTGGTCTCACCGGAGGCCTTGCCCTCTCGCTCTGCCGTTGGATGG : 6574
Ref    : CTGCCGACTCCATGGTGAGATTCAGACAGGACAAGACTTATTGGTTAGTTGGTCTCACCGGAGGCCTTGCCCTCTCGCTCTGCCGTTGGATGG : 6574
70-15  : CTGCCGACTCCATGGTGAGATTCAGACAGGACAAGACTTATTGGTTAGTTGGTCTCACCGGAGGCCTTGCCCTCTCGCTCTGCCGTTGGATGG : 6574

SCAU-2 : TGAATCGAGGTGCCAGATATGTCGTTATGACCAGCCGCAATCCAGTGATTGATAAAGAATGGCTTCATACCGTGGAATCTTGTGGTGCCACGG : 6657
NI907  : TGAATCGAGGTGCCAGATATGTCGTTATGACCAGCCGCAATCCAGTGATTGATAAAGAATGGCTTCATACCGTGGAATCTTGTGGTGCCACGG : 6657
W97-11 : TGAATCGAGGTGCCAGATATGTCGTTATGACCAGCCGCAATCCAGTGATTGATAAAGAATGGCTTCATACCGTGGAATCTTGTGGTGCCACGG : 6657
B71    : TGAACCGAGGGGCCAGATACGTCGTCATGACGAGCCGCAATCCAAAGATTGATAAGGAGTGGCTTCAAGGCGTGGAATCTTGTGGTGCTACGG : 6670
B2     : TGAACCGAGGTGCCAGATATGTCGTCATGACGAGCCGCAATCCAAAGATTGATAAGGAGTGGCTTCAAGGCGTGGAATCTTGTGGTGCTACGG : 6667
Ref    : TGAACCGAGGTGCCAGATATGTCGTCATGACGAGCCGCAATCCAAAGATTGATAAGGAGTGGCTTCAAGGCGTGGAATCTTGTGGTGCTACGG : 6667
70-15  : TGAACCGAGGTGCCAGATATGTCGTCATGACGAGCCGCAATCCAAAGATTGATAAGGAGTGGCTTCAAGGCGTGGAATCTTGTGGTGCTACGG : 6667
```

```
SCAU-2 : TAAAAATATTTTCCAAgtaagttggtttctctccttttccttgatgtgatgttcaaagcacagt-cact----gacccgtgtaaccctttttac :  6745
NI907  : TAAAAATATTTTCCAAgtaagttggtttctctccttttccttgatgtgatgttcaaagcacagt-cact----gacccgtgtaaccctttttac :  6745
W97-11 : TAAAAATATTTTCCAAgtaagttggtttctctccttttccttgatgtgatgttcaaagcacagt-cact----gacccgtgtaaccctttttac :  6745
B71    : TCAAGATATTTTCCAAgtaagttttgctttcgttgttcgtgatgt-catgtctaaactccagtctactaaaccacacaaccacttttgtttt :  6762
B2     : TCAAGATATTTTCCAAgtaagttttgcttttgttattcgcgatgt-catgtctaaactccagtctactaaaccgtacaaccactttt-cgtttt :  6758
Ref    : TCAAGATATTTTCCAAgtaagttttgcttttgttattcgcgatgt-catgtctaaactccagtctactaaaccgtacaaccactttt-cgtttt :  6758
70-15  : TCAAGATATTTTCCAAgtaagttttgcttttgttattcgcgatgt-catgtctaaactccagtctactaaaccgtacaaccactttt-cgtttt :  6758

SCAU-2 : tcttccccaaaatagCGACGTGACCGATCGCGCAGCAGTTAACTCAGCCTACCGCATTATCTCTGCCACCTTGCCACCGATCGCCGGCGTCG :  6838
NI907  : tcttccccaaaatagCGACGTGACCGATCGCGCAGCAGTTAACTCAGCCTACCGCATTATCTCTGCCACCTTGCCACCGATCGCCGGCGTCG :  6838
W97-11 : tcttccccaaaatagCGACGTGACCGATCGCGCAGCAGTTAACTCAGCCTACCGCATTATCTCTGCCACCTTGCCACCGATCGCCGGCGTCG :  6838
B71    : actcttcacccaatagCGACGTGACCGACCGTGCAGCAGTCAACTCGGCCTACCGTACCATTTCTGCCACCCTGCCTCCGATTGCCGGCGTCG :  6855
B2     : acccttgccccaatagCGACGTGACCGACCGTGCAGCAGTCAACTCAGCCTACCGTACCATTTCTGCCACCCTGCCTCCGATTGCCGGCGTCG :  6851
Ref    : acccttgccccaatagCGACGTGACCGACCGTGCAGCAGTCAACTCAGCCTACCGTACCATTTCTGCCACCCTGCCTCCGATTGCCGGCGTCG :  6851
70-15  : acccttgccccaatagCGACGTGACCGACCGTGCAGCAGTCAACTCAGCCTACCGTACCATTTCTGCCACCCTGCCTCCGATTGCCGGCGTCG :  6851

SCAU-2 : TCCAAGGCGCCATGGTCCTCCGGGATACCATGTTTGCCGAGACAACTATGGAAACGATAGAAACCGTTCTCGGGCCCAAGGTCCGCGGCAGCA :  6931
NI907  : TCCAAGGCGCCATGGTCCTCCGGGATACCATGTTTGCCGAGACAACTATGGAAACGATAGAAACCGTTCTCGGGCCCAAGGTCCGCGGCAGCA :  6931
W97-11 : TCCAAGGCGCCATGGTCCTCCGGGATACCATGTTTGCCGAGACAACTATGGAAACGATAGAAACCGTTCTCGGGCCCAAGGTCCGCGGCAGCA :  6931
B71    : TCCAAGGTGCCATGGTTCTCCGAGATACCATGTTTGCCGAGACAACCATGGAGACCATCGAAAGCATCCTCGGGCCCAAGGTCCGCGGTAGCA :  6948
B2     : TCCAAGGTGCCATGGTTCTCCGAGATACCATGTTTGCCGAGACAACCATGGAGACCATCGAAAGCATCCTCGGGCCCAAGGTCCGCGGTAGCA :  6944
Ref    : TCCAAGGTGCCATGGTTCTCCGAGATACCATGTTTGCCGAGACAACCATGGAGACCATCGAAAGCATCCTCGGGCCCAAGGTCCGCGGTAGCA :  6944
70-15  : TCCAAGGTGCCATGGTTCTCCGAGATACCATGTTTGCCGAGACAACCATGGAGACCATCGAAAGCATCCTCGGGCCCAAGGTCCGCGGTAGCA :  6944

SCAU-2 : TCTACCTCGACGAGATCTTCTATTCGACGCCTCTGGACTTTTTCGTCTTTCTGTCCTCTGTGACCTCTACATCCGGAAACCCCGGACAAAGCA :  7024
NI907  : TCTACCTCGACGAGATCTTCTATTCGACGCCTCTGGACTTTTTCGTCTTTCTGTCCTCTGTGACCTCTACATCCGGAAACCCCGGACAAAGCA :  7024
W97-11 : TCTACCTCGACGAGATCTTCTATTCGACGCCTCTGGACTTTTTCGTCTTTCTGTCCTCTGTGACCTCTACATCCGGAAACCCCGGACAAAGCA :  7024
B71    : TCTACCTCGACGAGATCTTCTACTCGACGCCTCTGGACTTTTTCGTCTTCCTGTCCTCTGTCACCGCCACATCTGGAAACCCCGGACAGAGCA :  7041
B2     : TCTACCTCGACGAGATATTCTACTCGACGCCTCTGGACTTTTTCGTCTTCCTGTCCTCTGTCACCGCCACATCTGGAAACCCCGGACAGAGCA :  7037
Ref    : TCTACCTCGACGAGATATTCTACTCGACGCCTCTGGACTTTTTCGTCTTCCTGTCCTCTGTCACCGCCACATCTGGAAACCCCGGACAGAGCA :  7037
70-15  : TCTACCTCGACGAGATATTCTACTCGACGCCTCTGGACTTTTTCGTCTTCCTGTCCTCTGTCACCGCCACATCTGGAAACCCCGGACAGAGCA :  7037
```

```
SCAU-2 : TTTATGCCGGAGCTAACATGTTTATGAATTCGCTTGCAGCGCAGCGCCGGAAGCGAGGTGTTGCTGGTTCGTCGGTAGAAATTGGTTGCATCA :  7117
NI907  : TTTATGCCGGAGCTAACATGTTTATGAATTCGCTTGCAGCGCAGCGCCGGAAGCGAGGTGTTGCTGGTTCGTCGGTAGAAATTGGTTGCATCA :  7117
W97-11 : TTTATGCCGGAGCTAACATGTTTATGAATTCGCTTGCAGCGCAGCGCCGGAAGCGAGGTGTTGCTGGTTCGTCGGTAGAAATTGGTTGCATCA :  7117
B71    : TCTACGCCGGAGCCAACATGTTTATGAACTCGCTTGCGGCCCAGCGCCGGAAGCGAGGCGTTGCCGGCTCCTCGGTGGAGATCGGCTGCATCA :  7134
B2     : TCTACGCCGGAGCCAACATGTTTATGAACTCGCTTGCGGCCCAGCGCCGGAAGCGAGGCGTTGCCGGCTCCTCGGTGGAGATCGGCTGCATCA :  7130
Ref    : TCTACGCCGGAGCCAACATGTTTATGAACTCGCTTGCGGCCCAGCGCCGGAAGCGAGGCGTTGCCGGCTCCTCGGTGGAGATCGGCTGCATCA :  7130
70-15  : TCTACGCCGGAGCCAACATGTTTATGAACTCGCTTGCGGCCCAGCGCCGGAAGCGAGGCGTTGCCGGCTCCTCGGTGGAGATCGGCTGCATCA :  7130

SCAU-2 : TGGGCAACGGCTCCGTCACGAGCATTTTGAGCTACGAGCACCAAAAATACCTCTTCTCGGTTGGGAATACTTGGTTGAGCGAACAAGACTTCC :  7210
NI907  : TGGGCAACGGCTCCGTCACGAGCATTTTGAGCTACGAGCACCAAAAATACCTCTTCTCGGTTGGGAATACTTGGTTGAGCGAACAAGACTTCC :  7210
W97-11 : TGGGCAACGGCTCCGTCACGAGCATTTTGAGCTACGAGCACCAAAAATACCTCTTCTCGGTTGGGAATACTTGGTTGAGCGAACAAGACTTCC :  7210
B71    : TGGGCAACGGCTCCGTGACGACCATTCTGAGCTACGAGCACCAAAAGTACCTCTTCTCGGTCGGCAACACGTGGCTGGCCGAGCAGGACTTCC :  7227
B2     : TGGGCAACGGCTCCGTGACGACCATTCTGAGCTACGAGCACCAAAAGTACCTCTTCTCGGTCGGCAACACGTGGCTGGCCGAGCAGGACTTCC :  7223
Ref    : TGGGCAACGGCTCCGTGACGACCATTCTGAGCTACGAGCACCAAAAGTACCTCTTCTCGGTCGGCAACACGTGGCTGGCCGAGCAGGACTTCC :  7223
70-15  : TGGGCAACGGCTCCGTGACGACCATTCTGAGCTACGAGCACCAAAAGTACCTCTTCTCGGTCGGCAACACGTGGCTGGCCGAGCAGGACTTCC :  7223

SCAU-2 : TTACTATGTTTGGCGAGGCGGTTCTCGCGAGCCCTCCGGACTCGTCGGACTCGGTCACCTCAGTAACTGGTCTGCGTCTTCAGTTCAATGACG :  7303
NI907  : TTACTATGTTTGGCGAGGCGGTTCTCGCGAGCCCTCCGGACTCGTCGGACTCGGTCACCTCAGTAACTGGTCTGCGTCTTCAGTTCAATGACG :  7303
W97-11 : TTACTATGTTTGGCGAGGCGGTTCTCGCGAGCCCTCCGGACTCGTCGGACTCGGTCACCTCAGTAACTGGTCTGCGTCTTCAGTTCAATGACG :  7303
B71    : TCACCATGTTTGGCGAGGCGGTCCTCGCGAGCCCACCCGACGCACCGGACTCGGTCACCTCGGTGACTGGTCTCCGTCTGCAGTTCAACGACG :  7320
B2     : TCACCATGTTTGGCGAGGCGGTCCTCGCGAGCCCACCCGACGCGCCGGACTCGGTCACCTCAGTGACTGGTCTCCGTCTGCAGTTCAATGACG :  7316
Ref    : TCACCATGTTTGGCGAGGCGGTCCTCGCGAGCCCACCCGACGCGCCGGACTCGGTCACCTCGGTGACTGGTCTCCGTCTGCAGTTCAATGACG :  7316
70-15  : TCACCATGTTTGGCGAGGCGGTCCTCGCGAGCCCACCCGACGCGCCGGACTCGGTCACCTCGGTGACTGGTCTCCGTCTGCAGTTCAATGACG :  7316

SCAU-2 : ACAAGCCAGATATTACGTGGTTCAGCAATCCAATCTTCCAACATCTAGTCCTGCAAAGTGGCAACGCCATGCAGACATCTTTGAGCGTGGTGA :  7396
NI907  : ACAAGCCAGATATTACGTGGTTCAGCAATCCAATCTTCCAACATCTAGTCCTGCAAAGTGGCAACGCCATGCAGACATCTTTGAGCGTGGTGA :  7396
W97-11 : ACAAGCCAGATATTACGTGGTTCAGCAATCCAATCTTCCAACATCTAGTCCTGCAAAGTGGCAACGCCATGCAGACATCTTTGAGCGTGGTGA :  7396
B71    : ACAAGCCGGATATCACGTGGTTCAGCAATCCGATTTTCCAACACTTGGTTCTGCAAAGTGGCAACGCCATGCAGACTTCCTTGAGCGTGGCGA :  7413
B2     : ACAAGCCGGATATCACGTGGTTCAGCAATCCAATTTTCCAACACTTGGTTCTGCAAAGTGGCAACGCCATGCAGACTTCCTTGAGCGTGGCGA :  7409
Ref    : ACAAGCCGGATATCACGTGGTTCAGCAATCCAATTTTCCAACACTTGGTTCTGCAAAGTGGCAACGCCATGCAGACTTCCTTGAGCGTGGCGA :  7409
70-15  : ACAAGCCGGATATCACGTGGTTCAGCAATCCAATTTTCCAACACTTGGTTCTGCAAAGTGGCAACGCCATGCAGACTTCCTTGAGCGTGGCGA :  7409
```

```
SCAU-2 : GGCAGGGTACTCCCGTAAAGACCCTTCTTCAAGACGCCAAGAGCTCCGAAGAAGTGCTTGAGATTCTGAAAGgtatgttatctatgcaccctt :  7489
NI907  : GGCAGGGTACTCCCGTAAAGACCCTTCTTCAAGACGCCAAGAGCTCCGAAGAAGTGCTTGAGATTCTGAAAGgtatgttatctatgcaccctt :  7489
W97-11 : GGCAGGGTACTCCCGTAAAGACCCTTCTTCAAGACGCCAAGAGCTCCGAAGAAGTGCTTGAGATTCTGAAAGgtatgttatctatgcaccctt :  7489
B71    : GGCAGGGTACTCCGGTAAAGAGCCTTCTTCAAGAGGCCAAGAGCTCTGAGGAAGTGCTCGACATTCTCAAAGgtattgttgcttatctgctca :  7506
B2     : GGCAGGGTACTCCGGTAAAGAGCCTTCTTCAAGAGGCCAAGAGCTCTGAGGAAGTGCTCGACATTCTCAAAGgtattgttgctcatctgctca :  7502
Ref    : GGCAGGGTACTCCGGTAAAGAGCCTTCTTCAAGAGGCCAAGAGCTCTGAGGAAGTGCTCGACATTCTCAAAGgtattgttgctcatctgctca :  7502
70-15  : GGCAGGGTACTCCGGTAAAGAGCCTTCTTCAAGAGGCCAAGAGCTCTGAGGAAGTGCTCGACATTCTCAAAGgtattgttgctcatctgctca :  7502

SCAU-2 : tctccaggctataaactagcacaggcgaatttggcaaagctgggtagtatactgacatattacacgactctagACGCATTCCAGGCAAAATTG :  7582
NI907  : tctccaggctataaactagcacaggcgaatttggcaaagctgggtagtatactgacatattacacgactctagACGCATTCCAGGCAAAATTG :  7582
W97-11 : tctccaggctataaactagcacaggcgaatttggcaaagctgggtagtatactgacatattacacgactctagACGCATTCCAGGCAAAATTG :  7582
B71    : tcctt------------------------tttctccgagctggttttactgacatgttccacgcgaatccagACGCATTCACGGCAAAGCTG :  7575
B2     : tcctt------------------------tttctccgagctggttttactgacatgttccacgcaaatccagACGCATTCACGGCAAAGCTG :  7571
Ref    : tcctt------------------------tttctccgagctggttttactgacatgttccacgcaaatccagACGCATTCACGGCAAAGCTG :  7571
70-15  : tcctt------------------------tttctccgagctggttttactgacatgttccacgcaaatccagACGCATTCACGGCAAAGCTG :  7571

SCAU-2 : GTCTCGTCCCTCCAAGCCCGACCCAGACAGCAACATCCTAGAGGTGGATCTGGAGACACTTGGCATGGATTCACTAGTCGCCGTAGACCTAAGG :  7675
NI907  : GTCTCGTCCCTCCAAGCCCGACCCAGACAGCAACATCCTAGAGGTGGATCTGGAGACACTTGGCATGGATTCACTAGTCGCCGTAGACCTAAGG :  7675
W97-11 : GTCTCGTCCCTCCAAGCCCGACCCAGACAGCAACATCCTAGAGGTGGATCTGGAGACACTTGGCATGGATTCACTAGTCGCCGTAGACCTAAGG :  7675
B71    : GTCTCGTCGCTCCAGGCTTGACCCAGACAGCAACCTCCTAGAGGTGGATCTGGAGACGCTTGGCATGGATTCACTGGTCGCCGTCGACCTGAGA :  7668
B2     : GTCTCGTCGCTCCAGGCCCGACCCAGACAGCAACCTCCTAGAGGTGGATCTGGAGACGCTTGGCATGGATTCACTGGTCGCCGTCGACCTGCGA :  7664
Ref    : GTCTCGTCGCTCCAGGCCCGACCCAGACAGCAACCTCCTAGAGGTGGATCTGGAGACGCTTGGCATGGATTCACTGGTCGCCGTCGACCTGCGA :  7664
70-15  : GTCTCGTCGCTCCAGGCCCGACCCAGACAGCAACCTCCTAGAGGTGGATCTGGAGACGCTTGGCATGGATTCACTGGTCGCCGTCGACCTGCGA :  7664

SCAU-2 : TCTTGGTTCCTAGCAGAGCTCTCTGTCGATGTTCCCGTCTTGAAAATTCTCAATGGAAGCACAGCCCGGTCGCTGCTCGAATTTGTCCAGGGA :  7768
NI907  : TCTTGGTTCCTAGCAGAGCTCTCTGTCGATGTTCCCGTCTTGAAAATTCTCAATGGAAGCACAGCCCGGTCGCTGCTCGAATTTGTCCAGGGA :  7768
W97-11 : TCTTGGTTCCTAGCAGAGCTCTCTGTCGATGTTCCCGTCTTGAAAATTCTCAATGGAAGCACAGCCCGGTCGCTGCTCGAATTTGTCCAGGGA :  7768
B71    : TCATGGTTCTTGGCAGAGCTTTCCGTCGATGTTCCCGTCTTGAAGATTCTGAACGGCAGCACGGCCCGGCTGCTGCTTGAATTTGTGCAGGGC :  7761
B2     : TCATGGTTCTTGGCAGAGCTTTCCGTCGATGTTCCCGTCTTGAAGATTCTGAACGGCAGCACTGCCCGGCTGCTGCTTGAATTTGTGCAGGGC :  7757
Ref    : TCATGGTTCTTGGCAGAGCTTTCCGTCGATGTTCCCGTCTTGAAGATTCTGAACGGCAGCACTGCCCGGCTGCTGCTTGAATTTGTGCAGGGC :  7757
70-15  : TCATGGTTCTTGGCAGAGCTTTCCGTCGATGTTCCCGTCTTGAAGATTCTGAACGGCAGCACTGCCCGGCTGCTGCTTGAATTTGTGCAGGGC :  7757
```

```
SCAU-2 : TTGATTCCCGCATCGATGACTCCGAAGCTCGACGGCTTAGATGGGGCCGATACAGCACCACAACAAGCACCGCCAGTGACGAAAACTC----- :  7856
NI907  : TTGATTCCCGCATCGATGACTCCGAAGCTCGACGGCTTAGATGGGGCCGATACAGCACCACAACAAGCACCGCCAGTGACGAAAACTC----- :  7856
W97-11 : TTGATTCCCGCATCGATGACTCCGAAGCTCGACGGCTTAGATGGGGCCGATACAGCACCACAACAAGCACCGCCAGTGACGAAAACTC----- :  7856
B71    : CTGATTCCCGCGTCCATGACTCCGAAGCTCGACGGCTCAGATGGAGCCGACGCCGCAGCACAAGAAGAAGCGCCGCCAGTGGCACCGCCAGTG :  7854
B2     : CTGATTCCCGCGTCCATGACTCCGAAGCTCGACGGCTCAGATGGAGCCGACGCCGCAGCACAA---GAAGCGCCGCCAGTGGCACCGCCAGTG :  7847
Ref    : CTGATTCCCGCGTCCATGACTCCGAAGCTCGACGGCTCAGATGGAGCCGACGCCGCAGCACAA---GAAGCGCCGCCAGTGGCACCGCCAGTG :  7847
70-15  : CTGATTCCCGCGTCCATGACTCCGAAGCTCGACGGCTCAGATGGAGCCGACGCCGCAGCACAA---GAAGCGCCGCCAGTGGCACCGCCAGTG :  7847

SCAU-2 : ----------AACCCGAAGTTTCAATAAAACTTCCAGGATCAGTGTCAAATTATCCACCGGTGGCTTCCATCAAGCAATCCGACTCCGCTTCC :  7939
NI907  : ----------AACCCGAAGTTTCAATAAAACTTCCAGGATCAGTGTCAAATTATCCACCGGTGGCTTCCATCAAGCAATCCGACTCCGCTTCC :  7939
W97-11 : ----------AACCCGAAGTTTCAATAAAACTTCCAGGATCAGTGTCAAATTATCCACCGGTGGCTTCCATCAAGCAATCCGACTCCGCTTCC :  7939
B71    : ACGAAGCCCAAGCCCGACGTTTCGGTCAAG---------GTGCCGCCGCCCCATCAACCGGTGGCTTCCCTCAAGCCATCTGGCCCCGCTTCT :  7938
B2     : ACGAAGCCCAAGCCCGACGTTTCGGTCAAG---------GTGCCGCCGCCCCATCAACCGGTGGCTTCCCTCAAGCCATCTGGCCCCGCTTCT :  7931
Ref    : ACGAAGCCCAAGCCCGACGTTTCGGTCAAG---------GTGCCGCCGCCCCATCAACCGGTGGCTTCCCTCAAGCCATCTGGCCCCGCTTCT :  7931
70-15  : ACGAAGCCCAAGCCCGACGTTTCGGTCAAG---------GTGCCGCCGCCCCATCAACCGGTGGCTTCCCTCAAGCCATCTGGCCCCGCTTCT :  7931

SCAU-2 : TCGTCCTCGCCATCACCAGAAGCCCGCTCCCCAGATCAGCCTCGGTCGGTTGCTTCATCAATGACTGATGACAGGCTCGATCTCAGCACCCCC :  8032
NI907  : TCGTCCTCGCCATCACCAGAAGCCCGCTCCCCAGATCAGCCTCGGTCGGTTGCTTCATCAATGACTGATGACAGGCTCGATCTCAGCACCCCC :  8032
W97-11 : TCGTCCTCGCCATCACCAGAAGCCCGCTCCCCAGATCAGCCTCGGTCGGTTGCTTCATCAATGACTGATGACAGGCTCGATCTCAGCACCCCC :  8032
B71    : CCGACCTCTCCATCGTCGGCAACAGCATCCCCAGGGCGGTCCCGCTCCGTTGCGTCACCAGTGACGGCCGACACG------CCCGTCTCTCCC :  8025
B2     : CCGACCTCTCCATCGTCGGCAACAGCATCCCCAGGGCGGTCCCGCTCCGTTGCCTCACCAGTGACGGCGGACACG------CCCGTCTCTCCC :  8018
Ref    : CCGACCTCTCCATCGTCGGCAACAGCATCCCCAGGGCGGTCCCGCTCCGTTGCCTCACCAGTGACGGCGGACACG------CCCGTCTCTCCC :  8018
70-15  : CCGACCTCTCCATCGTCGGCAACAGCATCCCCAGGGCGGTCCCGCTCCGTTGCCTCACCAGTGACGGCGGACACG------CCCGTCTCTCCC :  8018

SCAU-2 : ACGACATCCGCATCGTTTGCATCGCTCGATGATTCCAGGAAGCTCATACGAACAGTCCCTGTCTCCTTCGGTCAGGCTCGTTTTTGGTTTTTG :  8125
NI907  : ACGACATCCGCATCGTTTGCATCGCTCGATGATTCCAGGAAGCTCATACGAACAGTCCCTGTCTCCTTCGGTCAGGCTCGTTTTTGGTTTTTG :  8125
W97-11 : ACGACATCCGCATCGTTTGCATCGCTCGATGATTCCAGGAAGCTCATACGAACAGTCCCTGTCTCCTTCGGTCAGGCTCGTTTTTGGTTTTTG :  8125
B71    : ACGACTTCTGCATCGATGGCGTCGCTCAACGACTCGAGGAAGCTCATACGGACCGTCCCCGTCTCCTTTGGCCAGTCGCGCTTCTGGTTCCTG :  8118
B2     : ACGACTTCTGCATCGATGGCGTCGCTCAACGACTCGAGGAAGCTCATACGGACCGTCCCCGTCTCCTTTGGCCAGTCGCGCTTCTGGTTCCTG :  8111
Ref    : ACGACTTCTGCATCGATGGCGTCGCTCAACGACTCGAGGAAGCTCATACGGACCGTCCCCGTCTCCTTTGGCCAGTCGCGCTTCTGGTTCCTG :  8111
70-15  : ACGACTTCTGCATCGATGGCGTCGCTCAACGACTCGAGGAAGCTCATACGGACCGTCCCCGTCTCCTTTGGCCAGTCGCGCTTCTGGTTCCTG :  8111
```

```
SCAU-2 : AGGTCATACAATCCAGACCCGCTGGCGTTCAACATCACGTCGTTAATGCGCATTACTGGTCCCTTGCGTACTCCCGACTTTGCAAAGGCCGTC : 8218
NI907  : AGGTCATACAATCCAGACCCGCTGGCGTTCAACATCACGTCGTTAATGCGCATTACTGGTCCCTTGCGTACTCCCGACTTTGCAAAGGCCGTC : 8218
W97-11 : AGGTCATACAATCCAGACCCGCTGGCGTTCAACATCACGTCGTTAATGCGCATTACTGGTCCCTTGCGTACTCCCGACTTTGCAAAGGCCGTC : 8218
B71    : GGGTCGTACAACCCCGACCCGCTGGCCTTCAACATCACGTCGTTGATGCGCATCAGCGGCCCGTTGAGGACCAACGATTTCGCAAAGGCGGTC : 8211
B2     : GGGTCGTACAACCCCGACCCGCTGGCCTTCAACATCACGTCGTTGATGCGCATCAGCGGCCCGTTGAGGACCAACGATTTCGGCAAGGCCGTC : 8204
Ref    : GGGTCGTACAACCCCGACCCGCTGGCCTTCAACATCACGTCGTTGATGCGCATCAGCGGCCCGTTGAGGACCAACGATTTCGGCAAGGCCGTC : 8204
70-15  : GGGTCGTACAACCCCGACCCGCTGGCCTTCAACATCACGTCGTTGATGCGCATCAGCGGCCCGTTGAGGACCAACGATTTCGGCAAGGCCGTC : 8204

SCAU-2 : GAAAGGGTTCTCAACCACCACGAGGCACTTCGCACCTCTTTTGTGGAAGAGGATAATGGTCCGATGCAGAAAATATGGAGCTCGCCCGCTTTT : 8311
NI907  : GAAAGGGTTCTCAACCACCACGAGGCACTTCGCACCTCTTTTGTGGAAGAGGATAATGGTCCGATGCAGAAAATATGGAGCTCGCCCGCTTTT : 8311
W97-11 : GAAAGGGTTCTCAACCACCACGAGGCACTTCGCACCTCTTTTGTGGAAGAGGATAATGGTCCGATGCAGAAAATATGGAGCTCGCCCGCTTTT : 8311
B71    : GACAAGGTCCTCAACCACCACGAGGCCCTCCGCACCTCGTTCGTGTCGGAGAACGATGCCCCGGTGCAAAAGATTTGGAGCTCGCCCGCGTTT : 8304
B2     : GACAAGGTCCTCAACCACCACGAGGCCCTCCGCACCTCGTTCGTGTCGGAGAACGATGCCCCGGTGCAAAAGATTTGGAGCTCGCCCGCCTTT : 8297
Ref    : GACAAGGTCCTCAACCACCACGAGGCCCTCCGCACCTCGTTCGTGTCGGAGAACGATGCCCCGGTGCAAAAGATTTGGAGCTCGCCCGCCTTT : 8297
70-15  : GACAAGGTCCTCAACCACCACGAGGCCCTCCGCACCTCGTTCGTGTCGGAGAACGATGCCCCGGTGCAAAAGATTTGGAGCTCGCCCGCCTTT : 8297

SCAU-2 : GGACTCGAGCAACGCAAGATAATCGATGACGACAGCGCGGTCGTCAAGGCCTGCAAGGACGTCCAAAACACAGTCTATAACCTTGACCAAGGT : 8404
NI907  : GGACTCGAGCAACGCAAGATAATCGATGACGACAGCGCGGTCGTCAAGGCCTGCAAGGACGTCCAAAACACAGTCTATAACCTTGACCAAGGT : 8404
W97-11 : GGACTCGAGCAACGCAAGATAATCGATGACGACAGCGCGGTCGTCAAGGCCTGCAAGGACGTCCAAAACACAGTCTATAACCTTGACCAAGGT : 8404
B71    : ACGCTCGAGCAGCGCAAGATTGCCGACGACGAGAGCGAGGTTGTCAAGGCCTACACCGAGGTCCAAAACACGCGCTATAACCTCGAGGCGGGC : 8397
B2     : GCACTCGAGCAGCGCAAGATTGCCGACGACGAGAGCGAGGTTTTCAAGGCCTACACCGAGGTCCAAAACACACGCTATAACCTCGAGGCGGGC : 8390
Ref    : GCACTCGAGCAGCGCAAGATTGCCGACGACGAGAGCGAGGTTGTCAAGGCCTACACCGAGGTCCAAAACACACGCTATAACCTCGAGGCGGGC : 8390
70-15  : GCACTCGAGCAGCGCAAGATTGCCGACGACGAGAGCGAGGTTGTCAAGGCCTACACCGAGGTCCAAAACACACGCTATAACCTCGAGGCGGGC : 8390

SCAU-2 : CAGACGATGCGAATCCTACTTCTCACCAAGTCGCCGACGCAGCATGTACTCGTTCTCGGCTACCATCACATCAACATGGACGGAGTCAGCTTC : 8497
NI907  : CAGACGATGCGAATCCTACTTCTCACCAAGTCGCCGACGCAGCATGTACTCGTTCTCGGCTACCATCACATCAACATGGACGGAGTCAGCTTC : 8497
W97-11 : CAGACGATGCGAATCCTACTTCTCACCAAGTCGCCGACGCAGCATGTACTCGTTCTCGGCTACCATCACATCAACATGGACGGAGTCAGCTTC : 8497
B71    : CAGACCATGCGAATCATGCTTCTCACGAAATCGCCGACCAAGCACGTCCTCGTTCTCGGCTACCATCACATCAACATGGACGGAGTCAGCTTC : 8490
B2     : CAGACCATGCGAATCATGCTTCTCACGAAATCGCCGACCAAGCACGTCCTCGTTCTCGGCTACCATCACATCAACATGGACGGAGTCAGCTTC : 8483
Ref    : CAGACCATGCGAATCATGCTTCTCACGAAATCGCCGACCAAGCACGTCCTCGTTCTCGGCTACCATCACATCAACATGGACGGAGTCAGCTTC : 8483
70-15  : CAGACCATGCGAATCATGCTTCTCACGAAATCGCCGACCAAGCACGTCCTCGTTCTCGGCTACCATCACATCAACATGGACGGAGTCAGCTTC : 8483
```

```
SCAU-2 : GAGGTGCTTTTCAGCGACATCGAAAAGGCATACAACGGCCTAACCTCTGGATCGTTCAGTTATGCAGTTTCCCGACTTTACCATCAAGGAGTTC :  8590
NI907  : GAGGTGCTTTTCAGCGACATCGAAAAGGCATACAACGGCCTAACCTCTGGATCGTTCAGTTATGCAGTTTCCCGACTTTACCATCAAGGAGTTC :  8590
W97-11 : GAGGTGCTTTTCAGCGACATCGAAAAGGCATACAACGGCCTAACCTCTGGATCGTTCAGTTATGCAGTTTCCCGACTTTACCATCAAGGAGTTC :  8590
B71    : GAGGTGCTCTTCAGCGACATCGAAAAGGCCTACAACCGCACCCCTCTGGATCGTTCGGTGATGCAGTTCCCCGACTTTACCATCAGGGAAGCC :  8583
B2     : GAGGTGCTCTTTAGCGACATTGAAAAGGCCTACAACCGCACACCTCTGGATCGTTCGGTGATGCAGTTCCCCGACTTTACCATCAGGGAAGCC :  8576
Ref    : GAGGTGCTCTTTAGCGACATTGAAAAGGCCTACAACCGCACACCTCTGGATCGTTCGGTGATGCAGTTCCCCGACTTTACCATCAGGGAAGCC :  8576
70-15  : GAGGTGCTCTTTAGCGACATTGAAAAGGCCTACAACCGCACACCTCTGGATCGTTCGGTGATGCAGTTCCCCGACTTTACCATCAGGGAAGCC :  8576

SCAU-2 : AACGAGTTCAAGTCTGGCGGTTGGGAATCTGAGCTGCAGTATTGGAGAAGCAAATTCACCAGCCTTCCAGAGGCAACACCACTTTTATCCGTC :  8683
NI907  : AACGAGTTCAAGTCTGGCGGTTGGGAATCTGAGCTGCAGTATTGGAGAAGCAAATTCACCAGCCTTCCAGAGGCAACACCACTTTTATCCGTC :  8683
W97-11 : AACGAGTTCAAGTCTGGCGGTTGGGAATCTGAGCTGCAGTATTGGAGAAGCAAATTCACCAGCCTTCCAGAGGCAACACCACTTTTATCCGTC :  8683
B71    : GGCGAGTACAAGTCTGGGGCGTGGAGATCCGAGCTGCAGTATTGGCAGAGCAAATTCACCAGCCTCCCGGAGCCGACACCTCTCTTGTCCGTC :  8676
B2     : GGCGAGTACAAGTCTGGGGCGTGGAGATCCGAGCTGCAGTATTGGCAGAGCAAATTCACCAGCCTCCCCGAGCCGACACCTCTCTTGTCCGTC :  8669
Ref    : GGCGAGTACAAGTCTGGGGCGTGGAGATCCGAGCTGCAGTATTGGCAGAGCAAATTCACCAGCCTCCCCGAGCCGACACCTCTCTTGTCCGTC :  8669
70-15  : GGCGAGTACAAGTCTGGGGCGTGGAGATCCGAGCTGCAGTATTGGCAGAGCAAATTCACCAGCCTCCCCGAGCCGACACCTCTCTTGTCCGTC :  8669

SCAU-2 : TCTAAGCGAAGGTCGAGGCCCATCAATCTCTCGTACACAACGCATTCAATAAACCGCAGGATTAGCGCCGAGCAATCCAAGGCAATTCAAAGC :  8776
NI907  : TCTAAGCGAAGGTCGAGGCCCATCAATCTCTCGTACACAACGCATTCAATAAACCGCAGGATTAGCGCCGAGCAATCCAAGGCAATTCAAAGC :  8776
W97-11 : TCTAAGCGAAGGTCGAGGCCCATCAATCTCTCGTACACAACGCATTCAATAAACCGCAGGATTAGCGCCGAGCAATCCAAGGCAATTCAAAGC :  8776
B71    : TCGAAGCGAAGGACGAGGCCCGTCAACCTCAGCTACACGACGCACTCGGTGAGCCGCAGGATCAACGCCGAGCAGTCCCAGGCAATCCACACC :  8769
B2     : TCGAAGCGAAGGACGAGGCCCGTCAACCTCAGCTACACGACGCACTCGGTGAGCCGCAGGATCAACGCCGAGCAGTCCCAGGCAATCCACACC :  8762
Ref    : TCGAAGCGAAGGACGAGGCCCGTCAACCTCAGCTACACGACGCACTCGGTGAGCCGCAGGATCAACGCCGAGCAGTCCCAGGCAATCCACACC :  8762
70-15  : TCGAAGCGAAGGACGAGGCCCGTCAACCTCAGCTACACGACGCACTCGGTGAGCCGCAGGATCAACGCCGAGCAGTCCCAGGCAATCCACACC :  8762

SCAU-2 : GTTTCCCGTAAATTCAAGGCCACCCCGTTCCATTTTTACCTAGCCGTCTTCAAAACCTTGATCGCCCGCTTCAGTGGCACCGATGACTTTTGC :  8869
NI907  : GTTTCCCGTAAATTCAAGGCCACCCCGTTCCATTTTTACCTAGCCGTCTTCAAAACCTTGATCGCCCGCTTCAGTGGCACCGATGACTTTTGC :  8869
W97-11 : GTTTCCCGTAAATTCAAGGCCACCCCGTTCCATTTTTACCTAGCCGTCTTCAAAACCTTGATCGCCCGCTTCAGTGGCACCGATGACTTTTGC :  8869
B71    : GTTGGCCGTAAATTCAAGGCCACCCCCTTCCACTTCTACCTCTCGGTTTTCAAGACCTTGATCGCCCGCTTCAGCGGCGCCGACGACTTTTGC :  8862
B2     : GTTGGCCGTAAATTCAAGGCCACCCCCTTCCACTTCTACCTCTCGGTCTTCAAGACCTTGATCGCCCGCTTCAGCGGCGCCGACGACTTTTGC :  8855
Ref    : GTTGGCCGTAAATTCAAGGCCACCCCCTTCCACTTCTACCTCTCGGTCTTCAAGACCTTGATCGCCCGCTTCAGCGGCGCCGACGACTTTTGC :  8855
70-15  : GTTGGCCGTAAATTCAAGGCCACCCCCTTCCACTTCTACCTCTCGGTCTTCAAGACCTTGATCGCCCGCTTCAGCGGCGCCGACGACTTTTGC :  8855
```

```
SCAU-2 : ATTGGCATTGCCGACGCCAACAGAAAGGAAGAAAAAGTCATGGCTGCCGTTGGACTCTATCTCAATCTCCTACCCCTTCGTGTGCGGAGCTCA :  8962
NI907  : ATTGGCATTGCCGACGCCAACAGAAAGGAAGAAAAAGTCATGGCTGCCGTTGGACTCTATCTCAATCTCCTACCCCTTCGTGTGCGGAGCTCA :  8962
W97-11 : ATTGGCATTGCCGACGCCAACAGAAAGGAAGAAAAAGTCATGGCTGCCGTTGGACTCTATCTCAATCTCCTACCCCTTCGTGTGCGGAGCTCA :  8962
B71    : ATCGGGATCGCCGACGCCAACAGAAAGGAAGACAAGGTCATGGGTGCCGTGGGCCTCTATCTCAACCTCTTGCCCCTCCGCGTGCGGAGCGCA :  8955
B2     : ATCGGGATCGCCGACGCCAACAGAAAGGAAGACAAGGTCATGGGTGCCGTGGGCCTCTATCTCAACCTCTTGCCCCTCCGCGTGCGGAGCGCA :  8948
Ref    : ATCGGGATCGCCGACGCCAACAGAAAGGAAGACAAGGTCATGGGTGCCGTGGGCCTCTATCTCAACCTCTTGCCCCTCCGCGTGCGGAGCGCA :  8948
70-15  : ATCGGGATCGCCGACGCCAACAGAAAGGAAGACAAGGTCATGGGTGCCGTGGGCCTCTATCTCAACCTCTTGCCCCTCCGCGTGCGGAGCGCA :  8948

SCAU-2 : CTTGCGCAAACATTCGGGGAAGCTTTGGTAGATATGAAAAGGGTGTCGCAGGAGGCATTTGCCAATTCAAAGGTCCCCTTCGATGTCCTTCTT :  9055
NI907  : CTTGCGCAAACATTCGGGGAAGCTTTGGTAGATATGAAAAGGGTGTCGCAGGAGGCATTTGCCAATTCAAAGGTCCCCTTCGATGTCCTTCTT :  9055
W97-11 : CTTGCGCAAACATTCGGGGAAGCTTTGGTAGATATGAAAAGGGTGTCGCAGGAGGCATTTGCCAATTCAAAGGTCCCCTTCGATGTCCTTCTT :  9055
B71    : CTCGGGCAAACATTCGGTGAGACCCTGGCCGACATGAAGAAGGTGTCGCAGGAGGCGTTTGCCAATTCCAAGGTCCCCTTTGACGTCCTGCTC :  9048
B2     : CTCGGGCAAACATTCGGTGAGACCCTGGCCGACATGAAGAAGGTGTCGCAGGAGGCGTTTGCCAATTCCAAGGTCCCCTTTGACGTCCTGCTC :  9041
Ref    : CTCGGGCAAACATTCGGTGAGACCCTGGCCGACATGAAGAAGGTGTCGCAGGAGGCGTTTGCCAATTCCAAGGTCCCCTTTGACGTCCTGCTC :  9041
70-15  : CTCGGGCAAACATTCGGTGAGACCCTGGCCGACATGAAGAAGGTGTCGCAGGAGGCGTTTGCCAATTCCAAGGTCCCCTTTGACGTCCTGCTC :  9041

SCAU-2 : AACGAGCTCAACGTTCCACGCTCATCATCACATACGCCTCTGTTCCAGACTTTTGTCAACTACCGACGAGGCATCTCCGAAGAGCGGTCGTTC :  9148
NI907  : AACGAGCTCAACGTTCCACGCTCATCATCACATACGCCTCTGTTCCAGACTTTTGTCAACTACCGACGAGGCATCTCCGAAGAGCGGTCGTTC :  9148
W97-11 : AACGAGCTCAACGTTCCACGCTCATCATCACATACGCCTCTGTTCCAGACTTTTGTCAACTACCGACGAGGCATCTCCGAAGAGCGGTCGTTC :  9148
B71    : AACGAGCTCAGCGTCCCGCGCTCGTCGTCACAGACGCCTCTGTTCCAGACATTTGTCAACTACCGACGCGGTGTTTCCGAGGAGCGGTCGTTC :  9141
B2     : AACGAGCTCAGCGTGCCGCGCTCGTCGTCACACACGCCTCTGTTCCAGACGTTTGTCAACTACCGACGCGGTGTTTCCGAGGAGCGGTCGTTC :  9134
Ref    : AACGAGCTCAGCGTGCCGCGCTCGTCGTCACAGACGCCTCTGTTCCAGACGTTTGTCAACTACCGACGCGGTGTTTCCGAGGAGCGGTCGTTC :  9134
70-15  : AACGAGCTCAGCGTGCCGCGCTCGTCGTCACAGACGCCTCTGTTCCAGACGTTTGTCAACTACCGACGCGGTGTTTCCGAGGAGCGGTCGTTC :  9134

SCAU-2 : TGTGGCTGCACCGGCGCGGGCGAGCTTATCTCTGGTGGCCAAGTTGGTTACGATATAAGCCTGGATGTTGTCGAAAACCCTGACGGAGACGCT :  9241
NI907  : TGTGGCTGCACCGGCGCGGGCGAGCTTATCTCTGGTGGCCAAGTTGGTTACGATATAAGCCTGGATGTTGTCGAAAACCCTGACGGAGACGCT :  9241
W97-11 : TGTGGCTGCACCGGCGCGGGCGAGCTTATCTCTGGTGGCCAAGTTGGTTACGATATAAGCCTGGATGTTGTCGAAAACCCTGACGGAGACGCT :  9241
B71    : TGCGGCTGCACCGGCGCGGGCGAGCTCATCTCTGGCGGGCAGATTGGTTACGACATCAGCCTGGACATTGTCGAAAATCCCGGCGGCGACGCT :  9234
B2     : TGCGGCTGCACCGGCGCGGGCGAGCTCATCTCTGGCGGGCAGATTGGTTACGACATCAGCCTGGACATTGTCGAAAACCCCGGCGGCGACGCT :  9227
Ref    : TGCGGCTGCACCGGCGCGGGCGAGCTCATCTCTGGCGGGCAGATTGGTTACGACATCAGCCTGGACATTGTCGAAAACCCCGGCGGCGACGCT :  9227
70-15  : TGCGGCTGCACCGGCGCGGGCGAGCTCATCTCTGGCGGGCAGATTGGTTACGACATCAGCCTGGACATTGTCGAAAACCCCGGCGGCGACGCT :  9227
```

```
SCAU-2 : CTCGTGACTTTGAGTGTCCAGAAAGATCTATACGACATGGATATGGCCAACCTGCTGTTTGACAGCTATTTCCGGCTCGTCGACTCCTTTTCC :  9334
NI907  : CTCGTGACTTTGAGTGTCCAGAAAGATCTATACGACATGGATATGGCCAACCTGCTGTTTGACAGCTATTTCCGGCTCGTCGACTCCTTTTCC :  9334
W97-11 : CTCGTGACTTTGAGTGTCCAGAAAGATCTATACGACATGGATATGGCCAACCTGCTGTTTGACAGCTATTTCCGGCTCGTCGACTCCTTTTCC :  9334
B71    : CTGGTGACTTTGAGTGTCCAAAGGATCTGTACAACGTCGACATGGCGAATCTGCTGCTCGACAGCTACTTCCGGCTCGTCGACTCGTTTGCC :  9327
B2     : CTGGTGACTTTGAGTGTCCAAAGGATCTGTACAACGTCGACATGGCGAATCTGCTGCTCGACAGCTACTTCCGGCTCGTCGACTCCTTTGCC :  9320
Ref    : CTGGTGACTTTGAGTGTCCAAAGGATCTGTACAACGTCGACATGGCGAATCTGCTGCTCGACAGCTACTTCCGGCTCGTCGACTCCTTTGCC :  9320
70-15  : CTGGTGACTTTGAGTGTCCAAAGGATCTGTACAACGTCGACATGGCGAATCTGCTGCTCGACAGCTACTTCCGGCTCGTCGACTCCTTTGCC :  9320

SCAU-2 : AAAAATCCTGCCACCAGTTTGAACCGCCCTGCATTATATGACCCGGTGGCAGTCAAGAAGGCTTTGGAATTGGGCTGCGGGCCCAG---TCAA :  9424
NI907  : AAAAATCCTGCCACCAGTTTGAACCGCCCTGCATTATATGACCCGGTGGCAGTCAAGAAGGCTTTGGAATTGGGCTGCGGGCCCAG---TCAA :  9424
W97-11 : AAAAATCCTGCCACCAGTTTGAACCGCCCTGCATTATATGACCCGGTGGCAGTCAAGAAGGCTTTGGAATTGGGCTGCGGGCCCAG---TCAA :  9424
B71    : AAGAACCCCGCCACCAGCTTGAACCGTCCGGCCATATACGACCCCGTTGCGGTGGACAAGGCTTTGACGTTGGGCTGCGGCCCCACTCTTGAA :  9420
B2     : AAGAACCCCGCCACCAGCTTGAACCGTCCGGCCATATACGACCCCGTTGCGGTGGACAAGGCTTTGACGTTGGGCTGCGGCCCCACTCTTGAA :  9413
Ref    : AAGAACCCCGCCACCAGCTTGAACCGTCCGGCCATATACGACCCCGTTGCGGTGGACAAGGCTTTGACGTTGGGCTGCGGCCCCACTCTTGAA :  9413
70-15  : AAGAACCCCGCCACCAGCTTGAACCGTCCGGCCATATACGACCCCGTTGCGGTGGACAAGGCTTTGACGTTGGGCTGCGGCCCCACTCTTGAA :  9413

SCAU-2 : GACTTGAGCTGGCCAGAAACCCTGGTTCACCGAATCGAGGACATGTCTGTCAGATATGCAACAAAGTTTGCCTTGCGTAATGGTCAGAACGCT :  9517
NI907  : GACTTGAGCTGGCCAGAAACCCTGGTTCACCGAATCGAGGACATGTCTGTCAGATATGCAACAAAGTTTGCCTTGCGTAATGGTCAGAACGCT :  9517
W97-11 : GACTTGAGCTGGCCAGAAACCCTGGTTCACCGAATCGAGGACATGTCTGTCAGATATGCAACAAAGTTTGCCTTGCGTAATGGTCAGAACGCT :  9517
B71    : GACTCGAGCTGGCCAGAGACCCTGATCCACCGCATCGAAAACATGTCTGTCAAGTATGCGACAAAGTTTGCCTTGCGCAACGGCCAAAACGGT :  9513
B2     : GACTCGAGCTGGCCAGAGACCCTGATCCACCGCATCGAAAACATGTCTGTCAAGTATGCGACAAAGTTTGCCTTGCGCAACGGCCAAAACGGT :  9506
Ref    : GATTCGAGCTGGCCAGAGACCCTGATCCACCGCATCGAAAACATGTCTGTCAAGTATGCGACAAAGTTTGCCTTGCGCAACGGCCAAAACGGT :  9506
70-15  : GATTCGAGCTGGCCAGAGACCCTGATCCACCGCATCGAAAACATGTCTGTCAAGTATGCGACAAAGTTTGCCTTGCGCAACGGCCAAAACGGT :  9506

SCAU-2 : GGCCTTACTTATGCTCGGATGATTGCTCGGGTCAATGACATTGCAGCAAAACTTATCAAAGCCATGGTCGGTTCCAACCCTGGGATTGTGGGC :  9610
NI907  : GGCCTTACTTATGCTCGGATGATTGCTCGGGTCAATGACATTGCAGCAAAACTTATCAAAGCCATGGTCGGTTCCAACCCTGGGATTGTGGGC :  9610
W97-11 : GGCCTTACTTATGCTCGGATGATTGCTCGGGTCAATGACATTGCAGCAAAACTTATCAAAGCCATGGTCGGTTCCAACCCTGGGATTGTGGGC :  9610
B71    : GGCCTCACCTACTCTCAGATGATCGCCCGAATCAATGACATTGCGGCAAAACTGATTGACGCAAAGGTCGG------CTCTGGGATCGTGGGC :  9600
B2     : GGCCTCACGTACTCTCAGATGATTGCCCGAATCAATGACATTGCGGCAAAGCTGATTGACGCAAAGGTCGG------TACTGGAATCGTGGGC :  9593
Ref    : GGCCTCACGTACTCTCAGATGATTGCCCGAATCAATGACATTGCGGCAAAGCTGATTGACGCAAAGGTCGG------TACTGGAATCGTGGGC :  9593
70-15  : GGCCTCACGTACTCTCAGATGATTGCCCGAATCAATGACATTGCGGCAAAGCTGATTGACGCAAAGGTCGG------TACTGGAATCGTGGGC :  9593
```

```
SCAU-2 : GTGATGCAAGCTTCGACTATGGACTTTATTTGTTCAATCCTTGCCATTTGGAAGGCCGGTGCTATTTATACACCTTTGGATCCGCGCCTCAAT :  9703
NI907  : GTGATGCAAGCTTCGACTATGGACTTTATTTGTTCAATCCTTGCCATTTGGAAGGCCGGTGCTATTTATACACCTTTGGATCCGCGCCTCAAT :  9703
W97-11 : GTGATGCAAGCTTCGACTATGGACTTTATTTGTTCAATCCTTGCCATTTGGAAGGCCGGTGCTATTTATACACCTTTGGATCCGCGCCTCAAT :  9703
B71    : GTGATGCAGGCTTCCACCATGGACTTTATTTGTTCCATTCTTGCCGTTTGGAAGGCCGGTGCCATTTATACGCCTTTGGATCCACGCCTCAAC :  9693
B2     : GTGATGCAGGCTTCCACCATGGACTTTATTTGTTCCATTCTTGCCGTTTGGAAGGCCGGTGCCATTTATACGCCTTTGGATCCACGCCTCAAC :  9686
Ref    : GTGATGCAGGCTTCCACCATGGACTTTATTTGTTCCATTCTTGCCGTTTGGAAGGCCGGTGCCATTTATACGCCTTTGGATCCACGCCTCAAC :  9686
70-15  : GTGATGCAGGCTTCCACCATGGACTTTATTTGTTCCATTCTTGCCGTTTGGAAGGCCGGTGCCATTTATACGCCTTTGGATCCACGCCTCAAC :  9686

SCAU-2 : TCAGTCGATCGCCTCAGAGCCGTCGTGGACGAATGTCAGCCAATCTGCATCCTGGTAGATGCTACCACCAAGCCTTTATTCGATAGCCTGTCA :  9796
NI907  : TCAGTCGATCGCCTCAGAGCCGTCGTGGACGAATGTCAGCCAATCTGCATCCTGGTAGATGCTACCACCAAGCCTTTATTCGATAGCCTGTCA :  9796
W97-11 : TCAGTCGATCGCCTCAGAGCCGTCGTGGACGAATGTCAGCCAATCTGCATCCTGGTAGATGCTACCACCAAGCCTTTATTCGATAGCCTGTCA :  9796
B71    : TCAACCGATCGCCTCAAGGCCGTCGTGGACGAATGTCAGCCTGCATGCATCCTAGTGGACGCCACCACCAAGCCCTTGTTCGACAGCTTGGCA :  9786
B2     : TCAACCGATCGCCTCAAGGCCGTCGTGGACGAATGTCAGCCTGCATGCATCCTAGTGGACGCCACCACCAAGCCCTTGTTCGACAGCTTGGCA :  9779
Ref    : TCAACCGATCGCCTCAAGGCCGTCGTGGACGAATGTCAGCCTGCATGCATCCTAGTGGACGCCACCACCAAGCCCTTGTTCGACAGCTTGGCA :  9779
70-15  : TCAACCGATCGCCTCAAGGCCGTCGTGGACGAATGTCAGCCTGCATGCATCCTAGTGGACGCCACCACCAAGCCCTTGTTCGACAGCTTGGCA :  9779

SCAU-2 : TCCAAAGCTATCCAGATTGACGTATCGGAGGTTCAAAGTTCCAAAACGCTGGAACAATCACCAAAACTTGCGATACAAGCCAAGGGTGCCTCG :  9889
NI907  : TCCAAAGCTATCCAGATTGACGTATCGGAGGTTCAAAGTTCCAAAACGCTGGAACAATCACCAAAACTTGCGATACAAGCCAAGGGTGCCTCG :  9889
W97-11 : TCCAAAGCTATCCAGATTGACGTATCGGAGGTTCAAAGTTCCAAAACGCTGGAACAATCACCAAAACTTGCGATACAAGCCAAGGGTGCCTCG :  9889
B71    : ACCAACGCCGTCCAGATCGACGTTTCGATGGTGCAAAGCTCCAAAACGCTGGAAGCATCACCAAAGGTTGCCATACACGCAAAGGCGCCCTCG :  9879
B2     : ACCAACGCCGTCCAGATCGACGTTTCGATGGTGCAAAGCTCCAAAACGCTGGAAGCATCACCAAAGGTTGCCATACACGCAAAGGCGCCCTCG :  9872
Ref    : ACCAACGCCGTCCAGATCGACGTTTCGATGGTGCAAAGCTCCAAAACGCTGGAAGCATCACCAAAGGTTGCCATACACGCAAAGGCGCCCTCG :  9872
70-15  : ACCAACGCCGTCCAGATCGACGTTTCGATGGTGCAAAGCTCCAAAACGCTGGAAGCATCACCAAAGGTTGCCATACACGCAAAGGCGCCCTCG :  9872

SCAU-2 : GCGGCTGCCGTCTTTTACACAAGTGGTTCGACCGGCACCCCCAAGGGCATTTCACTGAGCCACACCTCTTTGACGTACAACATCATGGCCGCG :  9982
NI907  : GCGGCTGCCGTCTTTTACACAAGTGGTTCGACCGGCACCCCCAAGGGCATTTCACTGAGCCACACCTCTTTGACGTACAACATCATGGCCGCG :  9982
W97-11 : GCGGCTGCCGTCTTTTACACAAGTGGTTCGACCGGCACCCCCAAGGGCATTTCACTGAGCCACACCTCTTTGACGTACAACATCATGGCCGCG :  9982
B71    : GCGGCTGCCGTGTTTTACACGAGCGGTTCGACCGGCGTCCCCAAGGGCATCACACTAAGCCACGCCTCCCTGACGTACAACATCATGGCGGCG :  9972
B2     : GCGGCTGCCGTCTTTTACACGAGCGGTTCGACCGGCGTCCCCAAGGGCATCACACTAAGCCACGCCTCCCTGACGTACAACATCATGGCGGCG :  9965
Ref    : GCGGCTGCCGTCTTTTACACGAGCGGTTCGACCGGCGTCCCCAAGGGCATCACACTAAGCCACGCCTCCCTGACGTACAACATCATGGCGGCG :  9965
70-15  : GCGGCTGCCGTCTTTTACACGAGCGGTTCGACCGGCGTCCCCAAGGGCATCACACTAAGCCACGCCTCCCTGACGTACAACATCATGGCGGCG :  9965
```

```
SCAU-2 : ACACAGCAGTTCGGCTTCAAAGAGGGCATTGATATAATGCTCCAGCAATCGTCTTTCAGCTTTGATATGTCACTGGCGCAAATGCTAACATCG : 10075
NI907  : ACACAGCAGTTCGGCTTCAAAGAGGGCATTGATATAATGCTCCAGCAATCGTCTTTCAGCTTTGATATGTCACTGGCGCAAATGCTAACATCG : 10075
W97-11 : ACACAGCAGTTCGGCTTCAAAGAGGGCATTGATATAATGCTCCAGCAATCGTCTTTCAGCTTTGATATGTCACTGGCGCAAATGCTAACATCG : 10075
B71    : ACGCAGCAGTTCGGCTTCAAGGAGGGCGTCGACATCATGCTGCAGCAATCGTCCTTTAGCTTCGACATGGCATTGGCGCAGATGCTAACGTCG : 10065
B2     : ACGCAGCAGTTCGGCTTCAAGGAGGGCGTCGACATCATGCTGCAGCAATCGTCCTTTAGCTTCGACATGGCATTGGCGCAGATGCTTACGTCG : 10058
Ref    : ACGCGGCAGTTCGGCTTCAAGGAGGGCGTCGACATCATGCTGCAGCAATCGTCCTTTAGCTTCGACATGGCATTGGCGCAGATGCTTACGTCG : 10058
70-15  : ACGCGGCAGTTCGGCTTCAAGGAGGGCGTCGACATCATGCTGCAGCAATCGTCCTTTAGCTTCGACATGGCATTGGCGCAGATGCTTACGTCG : 10058

SCAU-2 : CTCTCCAATGGCGGCACCCTCGTCGTTGTCCCGTCCCACCTGCGTGGTGATGCTTTGGGCCTAAGCCAGCTAATCGTGGCGGAAAATGTTAGC : 10168
NI907  : CTCTCCAATGGCGGCACCCTCGTCGTTGTCCCGTCCCACCTGCGTGGTGATGCTTTGGGCCTAAGCCAGCTAATCGTGGCGGAAAATGTTAGC : 10168
W97-11 : CTCTCCAATGGCGGCACCCTCGTCGTTGTCCCGTCCCACCTGCGTGGTGATGCTTTGGGCCTAAGCCAGCTAATCGTGGCGGAAAATGTTAGC : 10168
B71    : CTCTCCAACGGCGGCACCCTCGTCGTGGTCCCGTCCCACCTGCGCGGCGACGCGTTGGGCCTGAGCCAGCTCATCGTGGCCGAGAACGTCAGC : 10158
B2     : CTCTCCAACGGCGGCACCCTCGTCGTGGTCCCGTCCCACCTGCGCGGCGACGCGTTGGGCCTGAGCCAGCTCATCGTGGCCGAGAACGTCAGC : 10151
Ref    : CTCTCCAACGGCGGCACCCTCGTCGTGGTCCCGTCCCACCTGCGCGGCGACGCGTTGGGCCTGAGCCAGCTCATCGTGGCCGAGAACGTCAGC : 10151
70-15  : CTCTCCAACGGCGGCACCCTCGTCGTGGTCCCGTCCCACCTGCGCGGCGACGCGTTGGGCCTGAGCCAGCTCATCGTGGCCGAGAACGTCAGC : 10151

SCAU-2 : ATTGTTCAGGCATCACCTACCGAATATAAGTCGCTGATCGGCGTTAACGCCCAGCAGCTCCGAACCTCCAAATGGAGAGTGGCTTTGAGCGGT : 10261
NI907  : ATTGTTCAGGCATCACCTACCGAATATAAGTCGCTGATCGGCGTTAACGCCCAGCAGCTCCGAACCTCCAAATGGAGAGTGGCTTTGAGCGGT : 10261
W97-11 : ATTGTTCAGGCATCACCTACCGAATATAAGTCGCTGATCGGCGTTAACGCCCAGCAGCTCCGAACCTCCAAATGGAGAGTGGCTTTGAGCGGT : 10261
B71    : ATCGTGCAGGCGTCACCCACGGAATACAAGTCGCTGATTGGCGTCAACGCCCAGCATCTCAAGACTTCCAAGTGGAGGGTGGCCTTGAGTGGT : 10251
B2     : ATCGTGCAGGCGTCACCCACGGAATACAAGTCGCTGATTGGCGTCAACGCCCAGCACCTCAAGACTTCCAAGTGGAGGGTGGCCTTGAGTGGT : 10244
Ref    : ATCGTGCAGGCGTCACCCACGGAATACAAGTCGCTGATTGGCGTCAACGCCCAGCACCTCAAGACTTCCAAGTGGAGGGTGGCCTTGAGTGGT : 10244
70-15  : ATCGTGCAGGCGTCACCCACGGAATACAAGTCGCTGATTGGCGTCAACGCCCAGCACCTCAAGACTTCCAAGTGGAGGGTGGCCTTGAGTGGT : 10244

SCAU-2 : GGCGAAAATATGACCCAAAACCTCCTGGAAGTTTTCCGCTCCCTCGGCAAGCCTGACCTCGTCCTTTATAATGGCTACGGTCCAACCGAGGCC : 10354
NI907  : GGCGAAAATATGACCCAAAACCTCCTGGAAGTTTTCCGCTCCCTCGGCAAGCCTGACCTCGTCCTTTATAATGGCTACGGTCCAACCGAGGCC : 10354
W97-11 : GGCGAAAATATGACCCAAAACCTCCTGGAAGTTTTCCGCTCCCTCGGCAAGCCTGACCTCGTCCTTTATAATGGCTACGGTCCAACCGAGGCC : 10354
B71    : GGCGAGAACATGACTCAGAGCCTCCTCGAGGTTTTCCGCTCGCTCGGCAAGCCCGACCTCGTCCTTTTCAACGGCTACGGGCCCACCGAGGCC : 10344
B2     : GGCGAGAACATGACTCAGAGCCTCCTCGAGGTTTTCCGCTCGCTCGGCAAGCCCGACCTCGTCCTTTTCAACGGCTACGGGCCCACCGAGGCC : 10337
Ref    : GGCGAGAACATGACTCAGAGCCTCCTCGAGGTTTTCCGCTCGCTCGGCAAGCCCGACCTCGTCCTTTTCAACGGCTACGGGCCCACCGAGGCC : 10337
70-15  : GGCGAGAACATGACTCAGAGCCTCCTCGAGGTTTTCCGCTCGCTCGGCAAGCCCGACCTCGTCCTTTTCAACGGCTACGGGCCCACCGAGGCC : 10337
```

```
SCAU-2 : ACTATCAACGCCAACACCCGCATAATACCCTACCACGAACCAAACAGCAATCCCGACCTGCCATTGTTGACCTGGGAAAACTACTCCATTTCG : 10447
NI907  : ACTATCAACGCCAACACCCGCATAATACCCTACCACGAACCAAACAGCAATCCCGACCTGCCATTGTTGACCTGGGAAAACTACTCCATTTCG : 10447
W97-11 : ACTATCAACGCCAACACCCGCATAATACCCTACCACGAACCAAACAGCAATCCCGACCTGCCATTGTTGACCTGGGAAAACTACTCCATTTCG : 10447
B71    : ACCATCAACGCCAACACCCGCATAGTGCCTTATCATGAGCCAAACAGCAACCCCGACCTGCCACTGCTGACCTGGCCAAACTATTCCATTTCC : 10437
B2     : ACCATCAACGCCAACACCCGCATAGTGCCTTATCATGAGCCGAACAGCAACCCCGACCTGCCACTGCTGACCTGGCCAAACTATTCCATTTCC : 10430
Ref    : ACCATCAACGCCAACACCCGCATAGTGCCTTATCATGAGCCGAACAGCAACCCCGACCTGCCACTGCTGACCTGGCCAAACTATTCCATTTCC : 10430
70-15  : ACCATCAACGCCAACACCCGCATAGTGCCTTATCATGAGCCGAACAGCAACCCCGACCTGCCACTGCTGACCTGGCCAAACTATTCCATTTCC : 10430

SCAU-2 : GTTGTCGATTTGGAGCTTAACCCGGTTCCAGTCGGCGTTTTTGGTGAAATTTGTATAGGTGGTGCCGGTGTAGGCCTGGGCTATTTCAAAAAC : 10540
NI907  : GTTGTCGATTTGGAGCTTAACCCGGTTCCAGTCGGCGTTTTTGGTGAAATTTGTATAGGTGGTGCCGGTGTAGGCCTGGGCTATTTCAAAAAC : 10540
W97-11 : GTTGTCGATTTGGAGCTTAACCCGGTTCCAGTCGGCGTTTTTGGTGAAATTTGTATAGGTGGTGCCGGTGTAGGCCTGGGCTATTTCAAAAAC : 10540
B71    : ATTGTCGATCTGGAGCTCAACCCGGTCCCGGTCGGCGTCTTCGGCGAGGTTTGTATCGGTGGTGCAGGCGTCGGCCTGGGCTACTTCAAAAAC : 10530
B2     : ATTGTCGATCTGGAGCTCAACCCGGTCCCGGTCGGCGTCTTCGGCGAGGTTTGTATCGGTGGTGCCGGCGTCGGCCTGGGCTACTTCAAAAAC : 10523
Ref    : ATTGTCGATCTGGAGCTCAACCCGGTCCCGGTCGGCGTCTTCGGCGAGGTTTGTATCGGTGGTGCCGGCGTCGGCCTGGGCTACTTCAAAAAC : 10523
70-15  : ATTGTCGATCTGGAGCTCAACCCGGTCCCGGTCGGCGTCTTCGGCGAGGTTTGTATCGGTGGTGCCGGCGTCGGCCTGGGCTACTTCAAAAAC : 10523

SCAU-2 : GAGGAGCTTACATCAAAGGCATTCGTGGCGGATAAGACGGCGCCTCCTGAATTCTTGGCCAAGGGCTGGAAAACCAAATATCGAACGGGCGAC : 10633
NI907  : GAGGAGCTTACATCAAAGGCATTCGTGGCGGATAAGACGGCGCCTCCTGAATTCTTGGCCAAGGGCTGGAAAACCAAATATCGAACGGGCGAC : 10633
W97-11 : GAGGAGCTTACATCAAAGGCATTCGTGGCGGATAAGACGGCGCCTCCTGAATTCTTGGCCAAGGGCTGGAAAACCAAATATCGAACGGGCGAC : 10633
B71    : GATGAACTCACGGCAAAGGCGTTCGTGGCCGACAAGACGGCGCCTGCCGAGTTCGTGGCCAAGGGCTGGAAGACCAAGTTCCGAACGGGCGAC : 10623
B2     : GATGAACTCACGGCAAAGGCATTCGTGGCCGACAAGACGGCGCCTGCCGAGTTCGTGGCCAAGGGCTGGAAGACCAAGTTCCGAACGGGCGAC : 10616
Ref    : GATGAACTCACGGCAAAGGCATTCGTGGCCGACAAGACGGCGCCTGCCGAGTTCGTGGCCAAGGGCTGGAAGACCAAGTTCCGAACGGGCGAC : 10616
70-15  : GATGAACTCACGGCAAAGGCATTCGTGGCCGACAAGACGGCGCCTGCCGAGTTCGTGGCCAAGGGCTGGAAGACCAAGTTCCGAACGGGCGAC : 10616

SCAU-2 : CTTGGACGCCTCAGTCCCGACGGCGGTCTGATTATCGAAGGTCGCATCGATGGCGACACTCAGATCAAGCTCCGGGGAATCCGCATTGACTTG : 10726
NI907  : CTTGGACGCATCAGTCCCGACGGCGGTCTGATTATCGAAGGTCGCATCGATGGCGACACTCAGATCAAGCTCCGGGGAATCCGCATTGACTTG : 10726
W97-11 : CTTGGACGCCTCAGTCCCGACGGCGGTCTGATTATCGAAGGTCGCATCGATGGCGACACTCAGATCAAGCTCCGGGGAATCCGCATTGACTTG : 10726
B71    : CTCGGACGCCTCAGTCCCGACGGCGGTCTGATCATCGAAGGTCGCATCGACGGCGACACTCAGGTCAAGCTCCGGGGCATGCGCATCGACCTG : 10716
B2     : CTCGGACGCCTCAGTCCCGACGGCGGTCTGATCATCGAAGGTCGCATCGACGGCGACACTCAGGTCAAGCTCCGAGGCATGCGCATCGACCTG : 10709
Ref    : CTCGGACGCCTCAGTCCCGACGGCGGTCTGATCATCGAAGGTCGCATCGACGGCGACACTCAGGTCAAGCTCCGGGGCATGCGCATCGACCTG : 10709
70-15  : CTCGGACGCCTCAGTCCCGACGGCGGTCTGATCATCGAAGGTCGCATCGACGGCGACACTCAGGTCAAGCTCCGGGGCATGCGCATCGACCTG : 10709
```

```
SCAU-2 : CAAAATGTTGAATCGGCTATCTTGGAAGCAGGCGCTGGCAAAATCATCGATGTAGCCGTGTCTCTCCGCCGAGGTGGTGCTGACGAGTCGGAC : 10819
NI907  : CAAAATGTTGAATCGGCTATCTTGGAAGCAGGCGCTGGCAAAATCATCGATGTAGCCGTGTCTCTCCGCCGAGGTGGTGCTGACGAGTCGGAC : 10819
W97-11 : CAAAATGTTGAATCGGCTATCTTGGAAGCAGGCGCTGGCAAAATCATCGATGTAGCCGTGTCTCTCCGCCGAGGTGGTGCTGACGAGTCGGAC : 10819
B71    : AAGAACATTGAATCGGCCATCTTGCAAGCCGGCGCCGGCAAGATCATCGACGCCGCCGTGTCCGTCCGCCGAGGTGGTGCCGACGAGTCGGAA : 10809
B2     : AAGAACATTGAATCGGCCATCTTGCAAGCCGGCGCCGGCAAGATCATCGACGCCGCCGTGTCCGTCCGCCGAGGTGGTGCCGACGAGTCGGAA : 10802
Ref    : AAGAACATTGAATCGGCCATCTTGCAAGCCGGCGCCGGCAAGATCATCGACGCCGCCGTGTCCGTCCGCCGAGGTGGTGCCGACGAGTCGGAA : 10802
70-15  : AAGAACATTGAATCGGCCATCTTGCAAGCCGGCGCCGGCAAGATCATCGACGCCGCCGTGTCCGTCCGCCGAGGTGGTGCCGACGAGTCGGAA : 10802

SCAU-2 : CCCCAGTACCTCGTCGGCCATGTCGTACTAGATTCCGACCAGATTTCCCCAGAACAGTCAGCAAGAATTCCTCGCTCAAATCGTTCCCCGCTTG : 10912
NI907  : CCCCAGTACCTCGTCGGCCATGTCGTACTAGATTCCGACCAGATTTCCCCAGAACAGTCAGCAAGAATTCCTCGCTCAAATCGTTCCCCGCTTG : 10912
W97-11 : CCCCAGTACCTCGTCGGCCATGTCGTACTAGATTCCGACCAGATTTCCCCAGAACAGTCAGCAAGAATTCCTCGCTCAAATCGTTCCCCGCTTG : 10912
B71    : CCGCAGTACCTCGTCGGCCACGTCGTGCTGGATGCCGATCAGACCCCCGAGGACAGCCAGCAAGACTTCCTCGCCCAGCTCATTCCCCGCCTC : 10902
B2     : CCGCAGTACCTCGTCGGCCACGTCGTGCTGGATGCCGATCAGACTCCCGAGGACAGCCAGCAAGACTTCCTCGCCCAGCTCATTCCCCGCCTC : 10895
Ref    : CCGCAGTACCTCGTCGGCCACGTCGTGCTGGATGCCGATCAGACTCCCGAGGACAGCCAGCAAGACTTCCTCGCCCAGCTCATTCCCCGCCTC : 10895
70-15  : CCGCAGTACCTCGTCGGCCACGTCGTGCTGGATGCCGATCAGACTCCCGAGGACAGCCAGCAAGACTTCCTCGCCCAGCTCATTCCCCGCCTC : 10895

SCAU-2 : AGGCTCCCCCAGCACATGAAGCCATCCCTGCTCGTGCCCATCCATTCCTTGCCCCAAACCGCCTCCCATAAACTTGACCGTCGTGCTTTGCAG : 11005
NI907  : AGGCTCCCCCAGCACATGAAGCCATCCCTGCTCGTGCCCATCCATTCCTTGCCCCAAACCGCCTCCCATAAACTTGACCGTCGTGCTTTGCAG : 11005
W97-11 : AGGCTCCCCCAGCACATGAAGCCATCCCTGCTCGTGCCCATCCATTCCTTGCCCCAAACCGCCTCCCATAAACTTGACCGTCGTGCTTTGCAG : 11005
B71    : AGGCTGCCCCGGCACATGAAGCCGTCCCTGCTGGTGCCCATCCGCGCCTTGCCCCAAACCGCCTCCCACAAACTTGACCGCCGCGCCCTGCAG : 10995
B2     : AGGCTGCCCCGGCACATGAAGCCGTCCCTGCTCGTGCCCATCCGCGCCTTGCCCCAAACCGCCTCCCACAAACTTGACCGCCGCGCTCTGCAG : 10988
Ref    : AGGCTGCCCCGGCACATGAAGCCGTCCCTGCTCGTGCCCATCCGCGCCTTGCCCCAAACCGCCTCCCACAAACTTGACCGCCGCGCTCTGCAG : 10988
70-15  : AGGCTGCCCCGGCACATGAAGCCGTCCCTGCTCGTGCCCATCCGCGCCTTGCCCCAAACCGCCTCCCACAAACTTGACCGCCGCGCTCTGCAG : 10988

SCAU-2 : GACCTGCCCATCTCGGATGAGAACCAGATTGTCC----------ATCAAGGTGCAGAACTTGGCTCCGATCAGGCCGAGATGTGGAAACTTTGG : 11089
NI907  : GACCTGCCCATCTCGGATGAGAACCAGATTGTCC----------ATCAAGGTGCAGAACTTGGCTCCGATCAGGCCGAGATGTGGAAACTTTGG : 11089
W97-11 : GACCTGCCCATCTCGGATGAGAACCAGATTGTCC----------ATCAAGGTGCAGAACTTGGCTCCGATCAGGCCGAGATGTGGAAACTTTGG : 11089
B71    : CAGCTGCCCATCTCCGACGCGGGCCAGATTGCCAAGCAGTCGCAGCAGGGTGCCGAACTCGGCTCCGATCAGGCTCGGATGTGGAAGCTCTGG : 11088
B2     : CAGCTGCCCATCTCGGACGCGGGCCAGATTGCCAAGCAGTCGCAGCAGGGTGCCGAACTCGGCTCCGATCAGGCTCGGATGTGGAAGCTCTGG : 11081
Ref    : CAGCTGCCCATCTCGGACGCGGGCCAGATTGCCAAGCAGTCGCAGCAGGGTGCCGAACTCGGCTCCGATCAGGCTCGGATGTGGAAGCTCTGG : 11081
70-15  : CAGCTGCCCATCTCGGACGCGGGCCAGATTGCCAAGCAGTCGCAGCAGGGTGCCGAACTCGGCTCCGATCAGGCTCGGATGTGGAAGCTCTGG : 11081
```

```
SCAU-2 : AAACAGGTTATCCCTAGCGACGTCAGTTCCAAGTATTCAATCACGCCCAGGTCTGACTTTTTCCATGTGGGCGGGACCTCGCTTCTTCTTGTG : 11182
NI907  : AAACAGGTTATCCCTAGCGACGTCAGTTCCAAGTATTCAATCACGCCCAGGTCTGACTTTTTCCATGTGGGCGGGACCTCGCTTCTTCTTGTG : 11182
W97-11 : AAACAGGTTATCCCTAGCGACGTCAGTTCCAAGTATTCAATCACGCCCAGGTCTGACTTTTTCCATGTGGGCGGGACCTCGCTTCTTCTTGTG : 11182
B71    : AAGCAGGTCATCCCGCGCGACGTCGTTTCGCAATATTCCATCACGCCGCAGTCTGACTTTTTCCACGTCGGCGGGACCTCGCTCCTCCTTGTG : 11181
B2     : AAGCAGGTCATCCCGCGCGACGTCGTTTCGCAATACTCCATCACGCCGCAGTCTGACTTTTTCCACGTCGGCGGGACCTCGCTCCTCCTTGTG : 11174
Ref    : AAGCAGGTCATCCCGCGCGACGTCGTTTCGCAATACTCCATCACGCCGCAGTCTGACTTTTTCCACGTCGGCGGGACCTCGCTCCTCCTTGTG : 11174
70-15  : AAGCAGGTCATCCCGCGCGACGTCGTTTCGCAATACTCCATCACGCCGCAGTCTGACTTTTTCCACGTCGGCGGGACCTCGCTCCTCCTTGTG : 11174

SCAU-2 : AATCTGCAAAGTTTGATCAGGAAAAAGCACGGCAGCGCTCCACCTTTGCACGCAATGTTTGAATCCAGCACAGTTGCATCCATGACCGACTTG : 11275
NI907  : AATCTGCAAAGTTTGATCAGGAAAAAGCACGGCAGCGCTCCACCTTTGCACGCAATGTTTGAATCCAGCACAGTTGCATCCATGACCGACTTG : 11275
W97-11 : AATCTGCAAAGTTTGATCAGGAAAAAGCACGGCAGCGCTCCACCTTTGCACGCAATGTTTGAATCCAGCACAGTTGCATCCATGACCGACTTG : 11275
B71    : AACCTGCAGAGCCTGATCGCAAGGGAGCACGGCCGCGCGCCACCCCTGCACGCAATGTTCGAGTCAAGCACGGTCGCGGCCATGACCGACTTG : 11274
B2     : AACCTGCAGAGCCTGATCGCAAGGGAGCACGGCCGCGCGCCACCCCTGCACGCAATGTTCGAGTCAAGCACGGTCGCGGCCATGACCGACTTG : 11267
Ref    : AACCTGCAGAGCCTGATCGCAAGGGAGCACGGCCGCGCGCCACCCCTGCACGCAATGTTCGAGTCAAGCACGGTCGCGGCCATGACCGACTTG : 11267
70-15  : AACCTGCAGAGCCTGATCGCAAGGGAGCACGGCCGCGCGCCACCCCTGCACGCAATGTTCGAGTCAAGCACGGTCGCGGCCATGACCGACTTG : 11267

SCAU-2 : GTGCTTTCAGATAATCCATCCGGTAATGGAGCCTTGATCGACTGGGAACAAGAAACTTCGATTCCGATGTTGGCTCCAAACGTTATCCCTGGC : 11368
NI907  : GTGCTTTCAGATAATCCATCCGGTAATGGAGCCTTGATCGACTGGGAACAAGAAACTTCGATTCCGATGTTGGCTCCAAACGTTATCCCTGGC : 11368
W97-11 : GTGCTTTCAGATAATCCATCCGGTAATGGAGCCTTGATCGACTGGGAACAAGAAACTTCGATTCCGATGTTGGCTCCAAACGTTATCCCTGGC : 11368
B71    : GTGCTCTCGGATGACGCATCCGGCAGCACCGCCCTGATCGACTGGGAACAAGAAACCTCGATCCCGACGCTGCCGCCACACATCATCCCCGGC : 11367
B2     : GTGCTCTCGGATGACGCATCCGGCAGCACCGCCCTGATCGACTGGGAACAAGAAACCTCGATCCCGACGCTGCCGCCACACATCATCCCCGGC : 11360
Ref    : GTGCTCTCGGATGACGCATCCGGCAGCACCGCCCTGATCGACTGGGAACAAGAAACCTCGATCCCGACGCTGCCGCCACACATCATCCCCGGC : 11360
70-15  : GTGCTCTCGGATGACGCATCCGGCAGCACCGCCCTGATCGACTGGGAACAAGAAACCTCGATCCCGACGCTGCCGCCACACATCATCCCCGGC : 11360

SCAU-2 : GGCGGTGCCAACAAAGTCTCTTTGCCGCCTAGGGTAGTCCTGATCACAGGTGCAACAGGCTTCCTCGGACGGCAGCTCGTCGAATTCCTCCTG : 11461
NI907  : GGCGGTGCCAACAAAGTCTCTTTGCCGCCTAGGGTAGTCCTGATCACAGGTGCAACAGGCTTCCTCGGACGGCAGCTCGTCGAATTCCTCCTG : 11461
W97-11 : GGCGGTGCCAACAAAGTCTCTTTGCCGCCTAGGGTAGTCCTGATCACAGGTGCAACAGGCTTCCTCGGACGGCAGCTCGTCGAATTCCTCCTG : 11461
B71    : GGGGCAGGGAACAAGGTCTCGGTGCCGCCCAGGGTGGTCCTGCTCACGGGCGCGACGGGGTTCCTGGGCCGCCAGCTCATGGCCTTTTTGCTG : 11460
B2     : GGGGCAGGGAACAAGGTCTCGGTGCCGCCCAGGGTGGTCCTGCTCACGGGCGCGACGGGGTTCCTGGGCCGCCAGCTCATGGCCTTTTTGCTG : 11453
Ref    : GGGGCAGGGAACAAGGTCTCGGTGCCGCCCAGGGTGGTCCTGCTCACGGGCGCGACGGGGTTCCTGGGCCGCCAGCTCATGGCCTTTTTGCTG : 11453
70-15  : GGGGCAGGGAACAAGGTCTCGGTGCCGCCCAGGGTGGTCCTGCTCACGGGCGCGACGGGGTTCCTGGGCCGCCAGCTCATGGCCTTTTTGCTG : 11453
```

```
SCAU-2 : CGTCAGCCCAACATTACGCGCATCCACTGCCTGGCCGTGCGTAACTCTCCT---CCTTCCTCGGAGCTCCCCTTCTCGGATCCCCGCGTTAGC : 11551
NI907  : CGTCAGCCCAACATTACGCGCATCCACTGCCTGGCCGTGCGTAACTCTCCT---CCTTCCTCGGAGCTCCCCTTCTCGGATCCCCGCGTTAGC : 11551
W97-11 : CGTCAGCCCAACATTACGCGCATCCACTGCCTGGCCGTGCGTAACTCTCCT---CCTTCCTCGGAGCTCCCCTTCTCGGATCCCCGCGTTAGC : 11551
B71    : CGCCAGCCCAGCGTCAAGCGCATCCACTGCCTGGCCGTGCGCGGCGCCGCCCTCCCTCGTCGGCGGCCCCCTTCTCGGACCCGCGCGTCAGC : 11553
B2     : CGCCAGCCCAGCGTCAAGCGCATCCACTGCCTGGCCGTGCGCGGCGGCGCTCCTCCCTCGTCGGCGGCCCCCTTCTCGGACCCGCGCGTCAGC : 11546
Ref    : CGCCAGCCCAGCGTCAAGCGCATCCACTGCCTGGCCGTGCGCGGCGGCGCTCCTCCCTCGTCGGCGGCCCCCTTCTCGGACCCGCGCGTCAGC : 11546
70-15  : CGCCAGCCCAGCGTCAAGCGCATCCACTGCCTGGCCGTGCGCGGCGGCGCTCCTCCCTCGTCGGCGGCCCCCTTCTCGGACCCGCGCGTCAGC : 11546

SCAU-2 : ATCCACCATGGGGATCTCAGTGCTCCACGCCTAGGCCTCGGTGAGGATGTCGCTGAATCCCTGTTCGCCGAAGCCGACGTCATCATTCACAAC : 11644
NI907  : ATCCACCATGGGGATCTCAGTGCTCCACGCCTAGGCCTCGGTGAGGATGTCGCTGAATCCCTGTTCGCCGAAGCCGACGTCATCATTCACAAC : 11644
W97-11 : ATCCACCATGGGGATCTCAGTGCTCCACGCCTAGGCCTCGGTGAGGATGTCGCTGAATCCCTGTTCGCCGAAGCCGACGTCATCATTCACAAC : 11644
B71    : ATCCACGCCGGCGACCTCAACGCTCCCCACCTGGGCCTCGGCGAGGCCGTGGCCGAGTCGCTGTTCGCCCAGGCCGACGTCATCATCCACAAC : 11646
B2     : ATCCACGCCGGCGACCTCAACGCTCCCCACCTGGGCCTCGGCGAGGCCGTGGCCGAGTCGCTGTTCGCCCAGGCCGACGTCATCATCCACAAC : 11639
Ref    : ATCCACGCCGGCGACCTCAACGCTCCCCACCTGGGCCTCGGCGAGGCCGTGGCCGAGTTGCTGTTCGCCCAGGCCGACGTCATCATCCACAAC : 11639
70-15  : ATCCACGCCGGCGACCTCAACGCTCCCCACCTGGGCCTCGGCGAGGCCGTGGCCGAGTTGCTGTTCGCCCAGGCCGACGTCATCATCCACAAC : 11639

SCAU-2 : GGCGCCGACGTGTCATTCCTCAAAACGTACGCCAGTCTGCGGCCGGTCAACGTCGGCAGCACACAGGAGCTAGCCCGTCTCGCGGCCCCGCGC : 11737
NI907  : GGCGCCGACGTGTCATTCCTCAAAACGTACGCCAGTCTGCGGCCGGTCAACGTCGGCAGCACACAGGAGCTAGCCCGTCTCGCGGCCCCGCGC : 11737
W97-11 : GGCGCCGACGTGTCATTCCTCAAAACGTACGCCAGTCTGCGGCCGGTCAACGTCGGCAGCACACAGGAGCTAGCCCGTCTCGCGGCCCCGCGC : 11737
B71    : GGCGCCGACGTGTCGTTCCTCAAGACCTACGCCACCCTGCGTGCGACCAACGTCGGCAGCACGCGGGAGCTGGCCCGCCTCGCTGCCCCGCGC : 11739
B2     : GGCGCCGACGTGTCGTTCCTCAAGACCTACGCCACCCTGCGCGCGACCAACGTCGGCAGCACGCGGGAGCTGGCCCGCCTCGCCGCCCCGCGC : 11732
Ref    : GGCGCCGACGTGTCGTTCCTCAAGACCTACGCCACCCTGCGCGCGACCAACGTCGGCAGCACGCGGGAGCTGGCCCGCCTCGCCGCCCCGCGC : 11732
70-15  : GGCGCCGACGTGTCGTTCCTCAAGACCTACGCCACCCTGCGCGCGACCAACGTCGGCAGCACGCGGGAGCTGGCCCGCCTCGCCGCCCCGCGC : 11732

SCAU-2 : CGAATCCCCTTCCACTTCGTATCGAGCGCCAGCATCACGCAGCTCACGGGTAAGGATGAGTTTGGCGAGGCCAGCCTCGCGGCCTGGGCCCCT : 11830
NI907  : CGAATCCCCTTCCACTTCGTATCGAGCGCCAGCATCACGCAGCTCACGGGTAAGGATGAGTTTGGCGAGGCCAGCCTCGCGGCCTGGGCCCCT : 11830
W97-11 : CGAATCCCCTTCCACTTCGTATCGAGCGCCAGCATCACGCAGCTCACGGGTAAGGATGAGTTTGGCGAGGCCAGCCTCGCGGCCTGGGCCCCT : 11830
B71    : CGCATCCCCTTCCACTTCGTGTCGAGCGCCAGCATCACGCAGCTGACGGGGCTGGACGAGTTTGGCGAGGCCAGCATGGCGGCCTGGGCCCCG : 11832
B2     : CGCATCCCCTTCCACTTTGTCTCGAGCGCCAGCATCACGCAGCTGACGGGGCTGGACGAGTTTGGCGAGGCCAGCATGGCGGCCTGGGCCCCG : 11825
Ref    : CGCATCCCCTTCCACTTTGTCTCGAGCGCCAGCATCACGCAGCTGACGGGGCTGGACGAGTTTGGCGAGGCCAGCATGGCGGCCTGGGCCCCG : 11825
70-15  : CGCATCCCCTTCCACTTTGTCTCGAGCGCCAGCATCACGCAGCTGACGGGGCTGGACGAGTTTGGCGAGGCCAGCATGGCGGCCTGGGCCCCG : 11825
```

```
SCAU-2 : CCTGCCGACCCTAGAGCTATGATGGGCGGTTATGTAGCCGCCAAGTGGGCGTCCGAAGTCCTTTTGGAAAAGGCGGCGCGTGCCTGGGGCCTA : 11923
NI907  : CCTGCCGACCCTAGAGCTATGATGGGCGGTTATGTAGCCGCCAAGTGGGCGTCCGAAGTCCTTTTGGAAAAGGCGGCGCGTGCCTGGGGCCTA : 11923
W97-11 : CCTGCCGACCCTAGAGCTATAATGGGCGGTTATGTAGCCGCCAAGTGGGCGTCCGAAGTCCTTTTGGAAAAGGCGGCGCGTGCCTGGGGCCTA : 11923
B71    : CCCACCGACCCCAGAGCCATGAGCGGCGGCTACGCAGCTGCCAAGTGGGCGTCCGAGGTCCTCTTGGAAAAGGCGGCACGGGCCTGGGGTCTG : 11925
B2     : CCCGCCGACCCCAGAGGCATGAGCGGCGGCTACGCGGCCGCCAAGTGGGCGTCCGAGGTCCTCTTGGAAAAGGCGGCGCGGGCCTGGGGTCTG : 11918
Ref    : CCCGCCGACCCCAGAGGCATGAGCGGCGGCTACGCGGCCGCCAAGTGGGCGTCCGAGGTCCTCTTGGAAAAGGCGGCGCGGGCCTGGGGTCTG : 11918
70-15  : CCCGCCGACCCCAGAGGCATGAGCGGCGGCTACGCGGCCGCCAAGTGGGCGTCCGAGGTCCTCTTGGAAAAGGCGGCGCGGGCCTGGGGTCTG : 11918

SCAU-2 : CCTGTTGTGATCCATCGGCCAAGCAGCATTACGGGTCAGAACGCGAATAGCCTCGACCTCATGGGCAACATGTTCAAGTATATCGAGCTACTC : 12016
NI907  : CCTGTTGTGATCCATCGGCCAAGCAGCATTACGGGTCAGAACGCGAATAGCCTCGACCTCATGGGCAACATGTTCAAGTATATCGAGCTACTC : 12016
W97-11 : CCTGTTGTGATCCATCGGCCAAGCAGCATTACGGGTCAGAACGCGAATAGCCTCGACCTCATGGGCAACATGTTCAAGTATATCGAGCTACTC : 12016
B71    : CCCGTCGTGATCCACCGGCCGAGCAGCATCACGGGCGAGGGCACCAATAGCCTCGACCTCATGGGCAACATGTTCAAGTACATTGAGCAGCTC : 12018
B2     : CCCGTCGTTATCCACCGGCCGAGCAGCATCACGGGCGAGGGCACCAATAGCCTCGACCTCATGGGCAACATGTTCAAGTACATTGAGCAGCTC : 12011
Ref    : CCCGTCGTTATCCACCGGCCGAGCAGCATCACGGGCGAGGGCACCAATAGCCTCGACCTCATGGGCAACATGTTCAAGTACATTGAGCAGCTC : 12011
70-15  : CCCGTCGTTATCCACCGGCCGAGCAGCATCACGGGCGAGGGCACCAATAGCCTCGACCTCATGGGCAACATGTTCAAGTACATTGAGCAGCTC : 12011

SCAU-2 : GAGGCTGTGCCCGAATCTGATTCTTGGAAAGGAAATTTTGATTTCGTTTCGGTCGAGAACGTCGCTGCGGACGTCGTCCAGGCAGTCGTTGCT : 12109
NI907  : GAGGCTGTGCCCGAATCTGATTCTTGGAAAGGAAATTTTGATTTCGTTTCGGTCGAGAACGTCGCTGCGGACGTCGTCCAGGCAGTCGTTGCT : 12109
W97-11 : GAGGCTGTGCCCGAATCTGATTCTTGGAAAGGAAATTTTGATTTCGTTTCGGTCGAGAACGTCGCTGCGGACGTCGTCCAGGCAGTCGTTGCT : 12109
B71    : GAGGCCGTGCCCGAGTCTGATTCCTGGAAGGGCAACTTTGACTTTGTGTCGGTCGAGAACGTCGCTGCCGACATCGTCCAGGCCGTCGTTGCC : 12111
B2     : GAGGCCGTGCCCGAGTCTGATTCCTGGAAGGGCAACTTTGACTTTGTGTCGGTCGAGAACGTCGCTGCCGACATCGTCCAGGCCGTCGTTGCC : 12104
Ref    : GAGGCCGTGCCCGAGTCTGATTCCTGGAAGGGCAACTTTGACTTTGTGTCGGTCGAGAACGTCGCTGCCGACATCGTCCAGGCCGTCGTTGCC : 12104
70-15  : GAGGCCGTGCCCGAGTCTGATTCCTGGAAGGGCAACTTTGACTTTGTGTCGGTCGAGAACGTCGCTGCCGACATCGTCCAGGCCGTCGTTGCC : 12104

SCAU-2 : GCGAACGCTGCAGCCAGCGGCGGGGTGAAATATATTTACGAGGCTGGAGACATCATCTATCCATTATCGATGGTCAAGGATATGTCCGAGGGT : 12202
NI907  : GCGAACGCTGCAGCCAGCGGCGGGGTGAAATATATTTACGAGGCTGGAGACATCATCTATCCATTATCGATGGTCAAGGATATGTCCGAGGGT : 12202
W97-11 : GCGAACGCTGCAGCCAGCGGCGGGGTGAAATATATTTACGAGGCTGGAGACATCATCTATCCATTATCGATGGTCAAGGATATGTCCGAGGGT : 12202
B71    : GCAAACGTTGTAGCCGCCGGCGGCGTCAAGTTTATCTACGAGGCTGGGGACATTGTTTATCCGTTGTCCATGGTCAAGGATATGTCTGAGGGG : 12204
B2     : GCAAACGTTGTGGCCGCCGGCGGCGTCAAGTTTATCTACGAGGCTGGGGACATTGTTTATCCGTTGTCCATGGTCAAGGATATGTCTGAGGGG : 12197
Ref    : GCAAACGTTGTGGCCGCCGGCGGCGTCAAGTTTATCTACGAGGCTGGGGACATTGTTTATCCGTTGTCCATGGTCAAGGATATGTCTGAGGGG : 12197
70-15  : GCAAACGTTGTGGCCGCCGGCGGCGTCAAGTTTATCTACGAGGCTGGGGACATTGTTTATCCGTTGTCCATGGTCAAGGATATGTCTGAGGGG : 12197
```

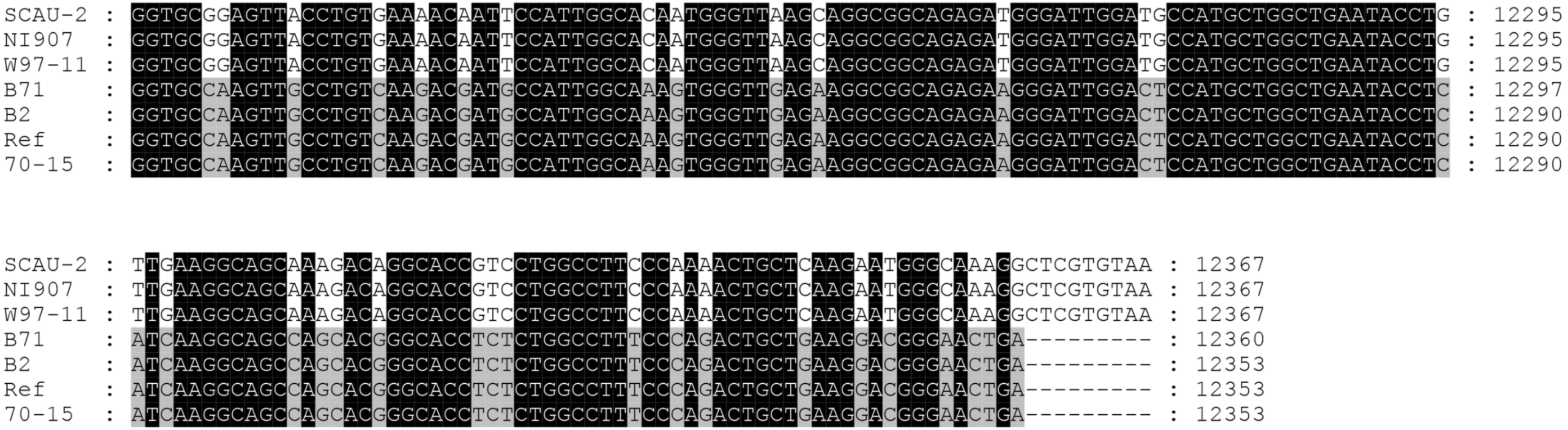

Figure S6. DNA sequence alignment of *ACE1*. Letters of uppercase denote to exons, and lowercase are introns. The sequence of referenced gene is accessible in NCBI under no. AJ704622.1. Sequence alignment was performed by Clustal X2 [49] and shaded by GeneDoc software [50]. The boxes with white character and black background represent that the conserved percentage is 100% in the column. The boxes with black character represent the difference of *ACE1* in different *Pyricularia* strains. Among the boxes with black character, the boxes with blue background represent that the conserved percentage is higher than or equal to 80% but less than 100% in the column, while the boxes with grey background represent the conserved percentage is higher than or equal to 50% but less than 80% in the column, and the boxes with white background represent the conserved percentage is less than 50% in the column.

```
SCAU-2 : MGDDMWTTNTEPIAIIGSGCRFPGGSTTPSKLWELLKDPKDIVSEIKPDRFDVDKYFHPDHKHHGTSNVRHSYFLDENFKLFDAKFFGIRPQEA :  94
NI907  : MGDDMWTTNTEPIAIIGSGCRFPGGSTTPSKLWELLKDPKDIVSEIKPDRFDVDKYFHPDHKHHGTSNVRHSYFLDENFKLFDAKFFGIRPQEA :  94
W97-11 : MGDDMWTTNTEPIAIIGSGCRFPGGSTTPSKLWELLKDPKDIVSEIKPDRFDVDKYFHPDHKHHGTSNVRHSYFLDENFKLFDAKFFGIRPQEA :  94
B71    : MRDEMWNTATEPIAIIGSGCKFPGGSTTPSKLWELLKDPKDIVSEIRPDRFDVDKYFHPDHKHHGTSNVRHSYFLEENFKHFDAKFFGIRPQEA :  94
B2     : MRDEMWNTATEPIAIIGSGCKFPGGSTTPSKLWELLKDPKDIVSEIRPDRFDVDKYFHPDHKHHGTSNVRHSYFLEENFKHFDAKFFGIRPQEA :  94
Ref    : MRDEMWNTATEPIAIIGSGCKFPGGSTTPSKLWELLKDPKDIVSEIRPDRFDVDKYFHPDHKHHGTSNVRHSYFLEENFKHFDAKFFGIRPQEA :  94
70-15  : MRDEMWNTATEPIAIIGSGCKFPGGSTTPSKLWELLKDPKDIVSEIRPDRFDVDKYFHPDHKHHGTSNVRHSYFLEENFKHFDAKFFGIRPQEA :  94

SCAU-2 : MAMDPQQRFLLETVYESLEAAGITIGGLKGSQTGVFVGNMGVDYSELLSQDIDAFPTYFAPGTARSILSNRISYFFDLHGPSVTVDTACSSSLV : 188
NI907  : MAMDPQQRFLLETVYESLEAAGITIGGLKGSQTGVFVGNMGVDYSELLSQDIDAFPTYFAPGTARSILSNRISYFFDLHGPSVTVDTACSSSLV : 188
W97-11 : MAMDPQQRFLLETVYESLEAAGITIGGLKGSQTGVFVGNMGVDYSELLSQDIDAFPTYFAPGTARSILSNRISYFFDLHGPSVTVDTACSSSLV : 188
B71    : MAMDPQQRFLLETVYESLEAAGITISDLKGSQAGVFVGNMGVDYSELLSQDIDAFPTYFAPGTARSILSNRISYFFDLHGPSVTVDTACSSSLV : 188
B2     : MAMDPQQRFLLETVYESLEAAGITISDLKGSQAGVFVGNMGVDYSELLSQDIDAFPTYFAPGTARSILSNRISYFFDLHGPSVTVDTACSSSLV : 188
Ref    : MAMDPQQRFLLETVYESLEAAGITISDLKGSQAGVFVGNMGVDYSELLSQDIDAFPTYFAPGTARSILSNRISYFFDLHGPSVTVDTACSSSLV : 188
70-15  : MAMDPQQRFLLETVYESLEAAGITISDLKGSQAGVFVGNMGVDYSELLSQDIDAFPTYFAPGTARSILSNRISYFFDLHGPSVTVDTACSSSLV : 188

SCAU-2 : AVHQAVQSLRLGETPVAIVCGANLLLGPAQYIAESKLQMLSPNGRSRMWDASADGYARGEGFASIVLKPLSAALANGDHIECIIRETGCNQDGR : 282
NI907  : AVHQAVQSLRLGETPVAIVCGANLLLGPAQYIAESKLQMLSPNGRSRMWDASADGYARGEGFASIVLKPLSAALANGDHIECIIRETGCNQDGR : 282
W97-11 : AVHQAVQSLRLGETPVAIVCGANLLLGPAQYIAESKLQMLSPNGRSRMWDASADGYARGEGFASIVLKPLSAALANGDHIECIIRETGCNQDGR : 282
B71    : AVHQAVQSLRLGETPVAIVCGANLLLGPAQYIAESKLQMLSPNGRSRMWDASADGYARGEGFASIVLKPLSVALANGDHIECIIRETGCNQDGR : 282
B2     : AVHQAVQSLRLGETPVAIVCGANLLLGPAQYIAESKLQMLSPNGRSRMWDASADGYARGEGFASIVLKPLSVALANGDHIECIIRETGCNQDGR : 282
Ref    : AVHQAVQSLRLGETPVAIVCGANLLLGPAQYIAESKLQMLSPNGRSRMWDASADGYARGEGFASIVLKPLSVALANGDHIECIIRETGCNQDGR : 282
70-15  : AVHQAVQSLRLGETPVAIVCGANLLLGPAQYIAESKLQMLSPNGRSRMWDASADGYARGEGFASIVLKPLSVALANGDHIECIIRETGCNQDGR : 282

SCAU-2 : TKGITMPSPLAQCKLIQETYRRAGLDLSKSSDRPQYFEAHGTGTPAGDPVEAEAISTAFFGPQSGYCRKSDDPKLYVGSVKTVIGHTEGTAGLA : 376
NI907  : TKGITMPSPLAQCKLIQETYRRAGLDLSKSSDRPQYFEAHGTGTPAGDPVEAEAISTAFFGPQSGYCRKSDDPKLYVGSVKTVIGHTEGTAGLA : 376
W97-11 : TKGITMPSPLAQCKLIQETYRRAGLDLSKSSDRPQYFEAHGTGTPAGDPVEAEAISTAFFGPQSGYCRKSDDPKLYVGSVKTVIGHTEGTAGLA : 376
B71    : TKGITMPSPLAQCKLIQETYKRAGLDLSKSSDRPQYFEAHGTGTPAGDPVEAEAISTAFFGPESGFRRTSHDPKLYVGSVKTVIGHTEGTAGLA : 376
B2     : TKGITMPSPLAQCKLIQETYKRAGLDLSKSSDRPQYFEAHGTGTPAGDPVEAEAISTAFFGPESGFRRTSHDPKLYVGSVKTVIGHTEGTAGLA : 376
Ref    : TKGITMPSPLAQCKLIQETYKRAGLDLSKSSDRPQYFEAHGTGTPAGDPVEAEAISTAFFGPESGFRRTSHDPKLYVGSVKTVIGHTEGTAGLA : 376
70-15  : TKGITMPSPLAQCKLIQETYKRAGLDLSKSSDRPQYFEAHGTGTPAGDPVEAEAISTAFFGPESGFRRTSHDPKLYVGSVKTVIGHTEGTAGLA : 376
```

```
SCAU-2 : GLIKASLAMKAKSIPPNLHLERVNPAVQPFYGNLEIPTRLMDWPEPEPGQPLRASVNSFGFGGANAHVILESYTPPA--VAALPPMPTSGPVFS : 468
NI907  : GLIKASLAMKAKSIPPNLHLERVNPAVQPFYGNLEIPTRLMDWPEPEPGQPLRASVNSFGFGGANAHVILESYTPPA--VAALPPMPTSGPVFS : 468
W97-11 : GLIKASLAMKAKSIPPNLHLERVNPAVQPFYGNLEIPTRLMDWPEPEPGQPLRASVNSFGFGGANAHVILESYTPPA--VAALPPMPTSGPVFS : 468
B71    : GLIKASLAMKAKSIPPNLHLERVNPAVQPFYGNLEIPTRLMDWPEPAPGQPLRASVNSFGFGGANAHVILESYNPPAAEVAMLPPTAAAGPVFS : 470
B2     : GLIKASLAMKAKSIPPNLHLERVNPAVQPFYGNLEIPTRLMDWPEPAPGQPLRASVNSFGFGGANAHVILESYTP-AAEVAMVTPTAAAGPVFS : 469
Ref    : GLIKASLAMKAKSIPPNLHLERVNPAVQPFYGNLEIPTRLMDWPEPAPGQPLRASVNSFGFGGANAHVILESYTP-AAEVAMVTPTAAAGPVFS : 469
70-15  : GLIKASLAMKAKSIPPNLHLERVNPAVQPFYGNLEIPTRLMDWPEPAPGQPLRASVNSFGFGGANAHVILESYTP-AAEVAMVTPTAAAGPVFS : 469
```

```
SCAU-2 : PFVFSASSDKALAGILSAYGEYLSQHPTVDLRSVAYTLSQHRSIFDKRAVISAADLDTLKSKLKARSEEASPSAK--AVQSLERRPRYLGIFTG : 560
NI907  : PFVFSASSDKALAGILSAYGEYLSQHPTVDLRSVAYTLSQHRSIFDKRAVISAADLDTLKSKLKARSEEASPSAK--AVQSLERRPRYLGIFTG : 560
W97-11 : PFVFSASSDKALAGILSAYGEYLSQHPTVDLRSVAYTLSQHRSIFDKRAVISAADLDTLKSKLKARSEEASPSAK--AVQSLERRPRYLGIFTG : 560
B71    : PFVFSASSDKALASMLSAYSDYLSLNPTVDLRSVAYTLSQHRSIFDKRAAISAPDLDTLKTKLKARSEEASPSGKTAAVQSLERRPRYLGVFTG : 564
B2     : PFVFSASSDKALASMLSAYSDYLSLNPTVDLRSVAYTLSQHRSVFDKRAAISAPDLDTLKTKLKARSEEASPSGKTAAVQSLERRPRYLGVFTG : 563
Ref    : PFVFSASSDKALASMLSAYSDYLSLNPTVDLRSVAYTLSQHRSVFDKRAAISAPDLDTLKTKLKARSEEASPSGKTAAVQSLERRPRYLGVFTG : 563
70-15  : PFVFSASSDKALASMLSAYSDYLSLNPTVDLRSVAYTLSQHRSVFDKRAAISAPDLDTLKTKLKARSEEASPSGKTAAVQSLERRPRYLGVFTG : 563
```

```
SCAU-2 : QGAQWARMGVDIITASPAARAIFEELEQSLQTLPEKERPSWSMLKELLAPPETSRVYQAHISQTVCTAVQILLVQLLRAAGVEFSCVVGHSSGE : 654
NI907  : QGAQWARMGVDIITASPAARAIFEELEQSLQTLPEKERPSWSMLKELLAPPETSRVYQAHISQTVCTAVQILLVQLLRAAGVEFSCVVGHSSGE : 654
W97-11 : QGAQWARMGVDIITASPAARAIFEELEQSLQTLPEKERPSWSMLKELLAPPETSRVYQAHISQTVCTAVQILLVQLLRAAGVEFSCVVGHSSGE : 654
B71    : QGAQWARMGVDVINASPAARAIFEDLEQSLKTLPEEDRPSWSMLEELLAPPETSRVYQANISQTVCTAVQVMMVQLLRAAGIEFSCVVGHSSGE : 658
B2     : QGAQWARMGVDVINASPAARAIFEDLEQSLKTLPEEDRPSWSMLEELLAPPETSRVYQANISQTVCTAVQVMMVQLLRAAGIEFSCVVGHSSGE : 657
Ref    : QGAQWARMGVDVINASPAARAIFEDLEQSLKTLPEEDRPSWSMLEELLAPPETSRVYQANISQTVCTAVQVMMVQLLRAAGIEFSCVVGHSSGE : 657
70-15  : QGAQWARMGVDVINASPAARAIFEDLEQSLKTLPEEDRPSWSMLEELLAPPETSRVYQANISQTVCTAVQVMMVQLLRAAGIEFSCVVGHSSGE : 657
```

```
SCAU-2 : IAAAYTAGYLSAKDAVRAAYFRGVHTHLAKGANGQPGGMIAVGTTLEDAKELCEVDDFKGRLCVAASNSNDSVTLSGDLDAVKEVKKVLDAEGK : 748
NI907  : IAAAYTAGYLSAKDAVRAAYFRGVHTHLAKGANGQPGGMIAVGTTLEDAKELCEVDDFKGRLCVAASNSNDSVTLSGDLDAVKEVKKVLDAEGK : 748
W97-11 : IAAAYTAGYLSAKDAVRAAYFRGVHTHLAKGANGQPGGMIAVGTTLEDAKELCEVDDFKGRLCVAASNSNDSVTLSGDLDAVKEVKKVLDAEGK : 748
B71    : MAAAYTAGYLSARDAVRAAYFRGVHSQLAKGSNGQPGGMIAVGTNFEDAEELCELDDFKGRLCVAASNSAELVTLSGDLDAVQEVKKILDAEEK : 752
B2     : MAAAYTAGYLSARDAVRAAYFRGVHSQLAKGSNGQPGGMIAVGTNFEDAEELCELDDFKGRLCVAASNSAELVTLSGDLDAVQEVKKILDAEEK : 751
Ref    : MAAAYTAGYLSARDAVRAAYFRGVHSQLAKGSNGQPGGMIAVGTNFEDAEELCELDDFKGRLCVAASNSAELVTLSGDLDAVQEVKKILDAEEK : 751
70-15  : MAAAYTAGYLSARDAVRAAYFRGVHSQLAKGSNGQPGGMIAVGTNFEDAEELCELDDFKGRLCVAASNSAELVTLSGDLDAVQEVKKILDAEEK : 751
```

```
SCAU-2 : FNKQLQVDKGYHSHHMLPCSEPYITSLQNCDIQARVPGDAKACRWISSVYVDDMASLDCRVQDKYWVENLAKPVLFSQALSYALGADDKFDCVI :  842
NI907  : FNKQLQVDKGYHSHHMLPCSEPYITSLQNCDIQARVPGDAKACRWISSVYVDDMASLDCRVQDKYWVENLAKPVLFSQALSYALGADDKFDCVI :  842
W97-11 : FNKQLQVDKGYHSHHMLPCSEPYITSLQNCDIQARVPGDAKACRWISSVYVDDMASLDCRVQDKYWVENLAKPVLFSQALSYALGADDKFDCVI :  842
B71    : FNKQLQVDKGYHSHHMLPCSEPYVASLQKCGIQAQVPGDATACRWISSVYVDDMTNLDCRVQDRYWIENLAKPVMFSQALSHALGGDDKFDCVI :  846
B2     : FNKQLQVDKGYHSHHMLPCSEPYVASLQKCGIQAQVPGDATACRWISSVYVDDMTNLDCRVQDRYWIENLAKPVMFSQALSHALGGDDKFDSVI :  845
Ref    : FNKQLQVDKGYHSHHMLPCSEPYVASLQKCGIQAQVPGDATACRWISSVYVDDMTNLDCRVQDRYWIENLAKPVMFSQALSHALGGDDKFDSVI :  845
70-15  : FNKQLQVDKGYHSHHMLPCSEPYVASLQKCGIQAQVPGDATACRWISSVYVDDMTNLDCRVQDRYWIENLAKPVMFSQALSHALGGDDKFDSVI :  845

SCAU-2 : EVGPHPALKGPASQIIQSCLGEKLPYFGCLNRGTNSNEAMAECLGGIWSSFGSSAVNLAAYEKFASGNCDQRLLKELPSYKWDHDVEYYFQSRL :  936
NI907  : EVGPHPALKGPASQIIQSCLGEKLPYFGCLNRGTNSNEAMAECLGGIWSSFGSSAVNLAAYEKFASGNCDQRLLKELPSYKWDHDVEYYFQSRL :  936
W97-11 : EVGPHPALKGPASQIIQSCLGEKLPYFGCLNRGTNSNEAMAECLGGIWSSFGSSAVNLAAYEKFASGNCDQRLLKELPSYKWDHDVEYYFQSRL :  936
B71    : EVGPHPALKGPASQTIQACLGERLPYFGCLSRGTDSNEAFAEFLGGVWSTFGSSAVDLAAYERFATGGCDQRLVKELPSYTWDHDVEHYFQSRL :  940
B2     : EVGPHPALKGPASQTIQACLGERLPYFGCLSRGTDSNEAFAEFLGGVWSTFGSSAVDLAAYERFATGGCDQRLVKGLPSYTWDHDVEHYFQSRL :  939
Ref    : EVGPHPALKGPASQTIQACLGERLPYFGCLSRGTDSNEAFAEFLGGVWSTFGSSAVDLAAYERFATGGCDQRLVKGLPSYTWDHDVEHYFQSRL :  939
70-15  : EVGPHPALKGPASQTIQACLGERLPYFGCLSRGTDSNEAFAEFLGGVWSTFGSSAVDLAAYERFATGGCDQRLVKGLPSYTWDHDVEHYFQSRL :  939

SCAU-2 : SKVVLHRGSSPNELLGTRLPDDSAAEVRWRNSLNPAEVPWLLQHSAQGQTVFPGTGYIATVLEAVKQLFESNGVQTVELRDFVIGNALVIEANA : 1030
NI907  : SKVVLHRGSSPNELLGTRLPDDSAAEVRWRNSLNPAEVPWLLQHSAQGQTVFPGTGYIATVLEAVKQLFESNGVQTVELRDFVIGNALVIEANA : 1030
W97-11 : SKVVLHRGSSPNELLGTRLPDDSAAEVRWRNSLNPAEVPWLLQHSAQGQTVFPGTGYIATVLEAVKQLFESNGVQTVELRDFVIGNALVIEANA : 1030
B71    : SKVVLHRSTPPNELLGTRLPDDTAGEVRWRNSLHPGELPWLLQHSAQGQTVFPGTGYIATTLEAVKQLFDSSGVQTVEIRDMVIGNALVIEANT : 1034
B2     : SKVVLHRSTPPNELLGTRLPDDTAGEVRWRNSLHPGELPWLLQHSAQGQTVFPGTGYIATTLEAVKQLFDSSGVQTVEIRDMVIGNALVIEANT : 1033
Ref    : SKVVLHRSTPPNELLGTRLPDDTAGEVRWRNSLHPGELPWLLQHSAQGQTVFPGTGYIATTLEAVKQLFDSSGVQTVEIRDMVIGNALVIEANT : 1033
70-15  : SKVVLHRSTPPNELLGTRLPDDTAGEVRWRNSLHPGELPWLLQHSAQGQTVFPGTGYIATTLEAVKQLFDSSGVQTVEIRDMVIGNALVIEANT : 1033

SCAU-2 : GVETLFSLTGINSQADRITAHFSFSSQQGNSTKLVENASGDLTVVLGKPSQDALPKNFPSVTQMKDIDEARFYEAIDKLGYGYEGPFRALSRLQ : 1124
NI907  : GVETLFSLTGINSQADRITAHFSFSSQQGNSTKLVENASGDLTVVLGKPSQDALPKNFPSVTQMKDIDEARFYEAIDKLGYGYEGPFRALSRLQ : 1124
W97-11 : GVETLFSLTGINSQADRITAHFSFSSQQGNSTKLVENASGDLTVVLGKPSQDALPKNFPSVTQMKDIDEARFYEAIDKLGYGYEGPFRALSRLQ : 1124
B71    : GVETLFSLTSINTQTDRITAHFSFCSQQGGSTKLVENASGDLVVLLGEPSEDALPRSFHPGTQMKDIDEERFYEAIDKLGYGYEGPFRALSQLQ : 1128
B2     : GVETLFSLTSINTQTDRITAHFSFCSQQGGSTKLVENASGDLVVLLGEPSEDALPRSFHPGTQMKDIDEERFYEAIDKLGYGYEGPFRALSQLQ : 1127
Ref    : GVETLFSLTFINTQTDRITAHFSFCSQQGGSTKLVENASGDLVVLLGEPSEDALPRSFHPGTQMKDIDEERFYEAIDKLGYGYEGPFRALSQLQ : 1127
70-15  : GVETLFSLTFINTQTDRITAHFSFCSQQGGSTKLVENASGDLVVLLGEPSEDALPRSFHPGTQMKDIDEERFYEAIDKLGYGYEGPFRALSQLQ : 1127
```

```
SCAU-2 : RRMGAATGFVAVPEKTKHFDQMVFHPAALDAMVQTILLAYCYPGDTRLQGISLPTGIDCIRFNYGMLSQAARPGSQLPFMSFTAFEGDDVLAGA : 1218
NI907  : RRMGAATGFVAVPEKTKHFDQMVFHPAALDAMVQTILLAYCYPGDTRLQGISLPTGIDCIRFNYGMLSQAARPGSQLPFMSFTAFEGDDVLAGA : 1218
W97-11 : RRMGAATGFVAVPEKTKHFDQMVFHPAALDAMVQTILLAYCYPGDTRLQGISLPTGIDCIRFNYGMLSQAARPGSQLPFMSFTAFEGDDVLAGA : 1218
B71    : RRMGAATGLVAIPEKTKHFDQMVLHPAALDAMVQTVLLAYCYPGDTRLQGISLPTGIDCIRFNYGMLSEAARPGCQLPFLSCTAFEGDDVLGGV : 1222
B2     : RRMGAATGLVAIPEKTKHFDQMVLHPAALDAMVQTVLLAYCYPGDTRLQGISLPTGIDCIRFNYGMLSEAARPGCQLPFLSCTAFEGDDVLGGV : 1221
Ref    : RRMGAATGLVAIPEKTKHFDQMVLHPAALDAMVQTVLLAYCYPGDTRLQGISLPTGIDCIRFNYGMLSEAARPGCQLPFLSCTAFEGDDVLGGV : 1221
70-15  : RRMGAATGLVAIPEKTKHFDQMVLHPAALDAMVQTVLLAYCYPGDTRLQGISLPTGIDCIRFNYGMLSEAARPGCQLPFLSCTAFEGDDVLGGV : 1221

SCAU-2 : GSDVGGDVDVFSEDKSFALVQLQGLHTKPLSPPSAATDLQIFSEMEWKIISPEGADIEVRGEKRAYVADLFTSIERVAYFYMRHVDREIGKDRS : 1312
NI907  : GSDVGGDVDVFSEDKSFALVQLQGLHTKPLSPPSAATDLQIFSEMEWKIISPEGADIEVRGEKRAYVADLFTSIERVAYFYMRHVDREIGKDRS : 1312
W97-11 : GSDVGGDVDVFSEDKSFALVQLQGLHTKPLSPPSAATDLQIFSEMEWKIISPEGADIEVRGEKRAYVADLFTSIERVAYFYMRHVDREIGKDRS : 1312
B71    : GGDVGGDVDVFSEDKRFALIQLQGLHTKPLSPPSAATDLQIFSEMEWKTASPEGADMEVRGEKRAYVADLYSSMERVAYFYMRHVDREIGKDRS : 1316
B2     : GGDVGGDVDVFSEDKRFALIQLQGLHTKPLSPPSAATDLQIFSEMEWKTASPEGADMEVRGEKRAYVADLYSSMERVAYFYMRHVDREIGKDRS : 1315
Ref    : GGDVGGDVDVFSEDKRFALIQLQGLHTKPLSPPSAATDLQIFSEMEWKTASPEGADMEVRGEKRAYVADLYSSMERVAYFYMRHVDREIGKDRS : 1315
70-15  : GGDVGGDVDVFSEDKRFALIQLQGLHTKPLSPPSAATDLQIFSEMEWKTASPEGADMEVRGEKRAYVADLYSSMERVAYFYMRHVDREIGKDRS : 1315

SCAU-2 : RLAAHHVRFLEWVDHMCGRVEQGTLPHINRKWDYDTRDDILKIIAKYPDSIDLELMHAVGENLCSVFRGEMNPLEPMVKNNMLNRFYTDALGMS : 1406
NI907  : RLAAHHVRFLEWVDHMCGRVEQGTLPHINRKWDYDTRDDILKIIAKYPDSIDLELMHAVGENLCSVFRGEMNPLEPMVKNNMLNRFYTDALGMS : 1406
W97-11 : RLAAHHVRFLEWVDHMCGRVEQGTLPHINRKWDYDTRDDILKIIAKYPDSIDLELMHAVGENLCSVFRGEMNPLEPMVKNNMLNRFYTDALGMS : 1406
B71    : GLAPHQVRFLEWVDHMCGRVEAGTLPHISRKWDHDTRQDILKIIAKYPDSIDLELMHAVGENLCSVFRGEMNVLEPMVKKNMLNRFYSDALGMS : 1410
B2     : GLAPHQVRFLEWVDHMCGRVEAGTLPHISRKWDHDTRQDILKIIAKYPDSIDLELMHAVGENLCSVFRGEMNALEPMVKKNMLNRFYSDALGMS : 1409
Ref    : GLAPHQVRFLEWVDHMCGRVEAGTLPHISRKWDHDTRQDILKIIAKYPDSIDLELMHAVGENLCSVFRGEMNALEPMVKKNMLNRFYSDALGMS : 1409
70-15  : GLAPHQVRFLEWVDHMCGRVEAGTLPHISRKWDHDTRQDILKIIAKYPDSIDLELMHAVGENLCSVFRGEMNALEPMVKKNMLNRFYSDALGMS : 1409

SCAU-2 : PYTEDLARMVEHITHRYPHMNILEVGAGTGGATKVMLRKLKDAFASYTYTDISSGFFADAREVFKAHESKMVFKTLDIEKEIADQGYEENSFDL : 1500
NI907  : PYTEDLARMVEHITHRYPHMNILEVGAGTGGATKVMLRKLKDAFASYTYTDISSGFFADAREVFKAHESKMVFKTLDIEKEIADQGYEENSFDL : 1500
W97-11 : PYTEDLARMVEHITHRYPHMNILEVGAGTGGATKVMLRKLKDAFASYTYTDISSGFFADAREVFKAHESKMVFKTLDIEKEIADQGYEENSFDL : 1500
B71    : PYTEDLARMVGHITHRYPHMNILEVGAGTGGATKVMLRRLQDAFASYTYTDISSGFFADARQVFKAHESKMLFKTLDIEKDIVDQGYEENSFDL : 1504
B2     : PYTEDLARMVGHITHRYPHMNILEVGAGTGGATKVMLRRLQDAFASYTYTDISSGFFADARQVFKAHESKMLFKTLDIEKDIVDQGYEENSFDL : 1503
Ref    : PYTEDLARMVGHITHRYPHMNILEVGAGTGGATKVMLRRLQDAFASYTYTDISSGFFADARQVFKAHESKMLFKTLDIEKDIVDQGYEENSFDL : 1503
70-15  : PYTEDLARMVGHITHRYPHMNILEVGAGTGGATKVMLRRLQDAFASYTYTDISSGFFADARQVFKAHESKMLFKTLDIEKDIVDQGYEENSFDL : 1503
```

```
SCAU-2 : VIANLVVHATADLDETMARLRRLVKPGGYLVLLEITNNDPLRFGFIFGPLPGWWLGGEDGRVHSPCVEVEWWDRVMKRSGFSGAEIVTPHHSLG : 1594
NI907  : VIANLVVHATADLDETMARLRRLVKPGGYLVLLEITNNDPLRFGFIFGPLPGWWLGGEDGRVHSPCVEVEWWDRVMKRSGFSGAEIVTPHHSLG : 1594
W97-11 : VIANLVVHATADLDETMARLRRLVKPGGYLVLLEITNNDPLRFGFIFGPLPGWWLGGEDGRVHSPCVEVEWWDRVMKRSGFSGAEIVTPHHSLG : 1594
B71    : VIANLVVHATADLDATMGRLRRLVKPGGHLVLLEITTNDPLRFGFIFGPLPGWWLGGEDGRVHSPCVDVEWWDRVMKRNGFSGADIVTPHHTLG : 1598
B2     : VIANLVVHATADLDATMGRLRRLVKPGGHLVLLEITTNDPLRFGFIFGPLPGWWLGGEDGRVHSPCVDVEWWDRVMKRNGFSGADIVTPHHTLG : 1597
Ref    : VIANLVVHATADLDATMGRLRRLVKPGGHLVLLEITTNDPLRFGFIFGPLPGWWLGGEDGRVHSPCVDVEWWDRVMKRNGFSGADIVTPHHTLG : 1597
70-15  : VIANLVVHATADLDATMGRLRRLVKPGGHLVLLEITTNDPLRFGFIFGPLPGWWLGGEDGRVHSPCVDVEWWDRVMKRNGFSGADIVTPHHTLG : 1597

SCAU-2 : PLSVIMTQAVDDRVRLLKEPTTADYKEFTIDPERLTIVGGASQLAKGLEQLLKSHYQTVTWIPNLEDVSSQSLPVMGSVLSLVELDEPLFKDMT : 1688
NI907  : PLSVIMTQAVDDRVRLLKEPTTADYKEFTIDPERLTIVGGASQLAKGLEQLLKSHYQTVTWIPNLEDVSSQSLPVMGSVLSLVELDEPLFKDMT : 1688
W97-11 : PLSVIMTQAVDDRVRLLKEPTTADYKEFTIDPERLTIVGGASQLAKGLEQLLKSHYQTVTWIPNLEDVSSQSLPVMGSVLSLVELDEPLFKDMT : 1688
B71    : PLSVIMTQAVDNRVQLLRQPTSADFGDFAIDPERLTIVGGVKPLAEGLEQLLKPRYQTVTWIPTLEEVSSQSLPVMGSVLSLVELDEPLFKDMT : 1692
B2     : PLSVIMTQAVDHRVQLLRQPTSADFGDFTIDPERLTIVGGVKPLAEGLEQLLKPRYQSVAWIPTLEEVSSHSLPVMGSVLSLVELDEPLFKDMT : 1691
Ref    : PLSVIMTQAVDHRVQLLRQPTSADFGDFTIDPERLTIVGGVKPLAEGLEQLLKPRYQSVAWIPTLEEVSSHSLPVMGSVLSLVELDEPLFKDMT : 1691
70-15  : PLSVIMTQAVDHRVQLLRQPTSADFGDFTIDPERLTIVGGVKPLAEGLEQLLKPRYQSVAWIPTLEEVSSHSLPVMGSVLSLVELDEPLFKDMT : 1691

SCAU-2 : VQTLEGFKLVFQQSRSVYWITCGASGANPYSNMAAGVARTVGLEMRHLRLGFLDFENSQDATVHNLSESFLQFEILGTLEQQGKLDRLTWYQEP : 1782
NI907  : VQTLEGFKLVFQQSRSVYWITCGASGANPYSNMAAGVARTVGLEMRHLRLGFLDFENSQDATVHNLSESFLQFEILGTLEQQGKLDRLTWYQEP : 1782
W97-11 : VQTLEGFKLVFQQSRSVYWITCGASGANPYSNMAAGVARTVGLEMRHLRLGFLDFENSQDATVHNLSESFLQFEILGTLEQQGKLDRLTWYQEP : 1782
B71    : AQTLEGFKLVFQQSRSVYWITCGASGANPYSNMAAGVARTVALEMRHLRLGFLDFEDAKDATVQRLADRFLEFEILGTLEQQGKLDRLTWYQEP : 1786
B2     : AQTLEGFKFVFQQSRSVYWITCGASGANPYSNMAAGVARTVALEMRHLRLGFLDFEDAKDATVQRLADRFLEFEILGTLEQQGKLDHLTWYQEP : 1785
Ref    : AQTLEGFKFVFQQSRSVYWITCGASGANPYSNMAAGVARTVALEMRHLRLGFLDFEDAKDATVQRLADRFLEFEILGTLEQQGKLDHLTWYQEP : 1785
70-15  : AQTLEGFKFVFQQSRSVYWITCGASGANPYSNMAAGVARTVALEMRHLRLGFLDFEDAKDATVQRLADRFLEFEILGTLEQQGKLDHLTWYQEP : 1785

SCAU-2 : ELRFDGSNFLVPRIRLSKDRNARYNSRRRQLRKNVNPREVSVSLVPS--GKGFVLEESLHTSLSSTKHGQDMVTLRVHYAMHRSVRLESSDYLF : 1874
NI907  : ELRFDGSNFLVPRIRLSKDRNARYNSRRRQLRKNVNPREVSVSLVPS--GKGFVLEESLHTSLSSTKHGQDMVTLRVHYAMHRSVRLESSDYLF : 1874
W97-11 : ELRFDGSNFLVPRIRLSKDRNARYNSRRRQLRKNVNPREVSVSLVPS--GKGFVLEESLHTSLSSTKHGQDMVTLRVHYAMHRSVRLESSDYLF : 1874
B71    : ELRFDGKNLLVPRMKLSKDRNGRYNSRRRQLTKNVNPREVPVSLVPTTSGKDFVLKESLSSS-STKHGAQDTVSLRVHYASQRSLRLESSDYLF : 1879
B2     : ELRFDGKNLLVPRMKLSKDRNGRYNSRRRQLTKNVNPREVPVSLVPTTSGKDFVLKESLSSS-STKHGAQDTVSLRVHYASQRSLRLESSDYLF : 1878
Ref    : ELRFDGKNLLVPRMKLSKDRNGRYNSRRRQLTKNVNPREVPVSLVPTTSGKDFVLKESLSSS-STKHGAQDTVSLRVHYASQRSLRLESSDYLF : 1878
70-15  : ELRFDGKNLLVPRMKLSKDRNGRYNSRRRQLTKNVNPREVPVSLVPTTSGKDFVLKESLSSS-STKHGAQDTVSLRVHYASQRSLRLESSDYLF : 1878
```

```
SCAU-2 : LVLGTDISSGEAMFALADSQKSIVQVDRQWTAPYVGNLADGKHALAGLYTQIIASTVVAVLSSGDSLVVLDAETSLSLALSARCDSKGVRLTLL : 1968
NI907  : LVLGTDISSGEAMFALADSQKSIVQVDRQWTAPYVGNLADGKHALAGLYTQIIASTVVAVLSSGDSLVVLDAETSLSLALSARCDSKGVRLTLL : 1968
W97-11 : LVLGTDISSGEAMFALADSQKSIVQVDRQWTAPYVGNLADGKHALAGLYTQIIASTVVAVLSSGDSLVVLDAETSLSLALSARCDSKGVRLTLL : 1968
B71    : LVLGTNLSSGEAMFALADSNRSIVHVDRQWTTSYLGNLDHGRHALAGLYTQIMASTVVAGLSAGDSLVVLDAETPLSLALSARCAAKGVRLTLL : 1973
B2     : LVLGTNLSSGEAMFALADSNRSIVHVDRQWTTSYLGNLDHGRHALAGLYTQIMASTVVAGLSAGDSLVVLDAETPLSQALSARCAAKGVRLTLL : 1972
Ref    : LVLGTNLSSGEAMFALADSNRSIVHVDRQWTTSYLGNLDHGRHALADLYTQIMASTVVAGLSAGDSLVVLDAETPLSQALSARCAAKGVRLTLL : 1972
70-15  : LVLGTNLSSGEAMFALADSNRSIVHVDRQWTTSYLGNLDHGRHALADLYTQIMASTVVAGLSAGDSLVVLDAETPLSQALSARCAAKGVRLTLL : 1972

SCAU-2 : STTSPDSDSS---AANKTVRIHPFESRRSIESKLPTSTTCFLNLSSSKENVTADVINSYIPTQCRVETRDSLTALVGQITRSTSM-GLTSAVAD : 2058
NI907  : STTSPDSDSS---AANKTVRIHPFESRRSIESKLPTSTTCFLNLSSSKENVTADVINSYIPTQCRVETRDSLTALVGQITRSTSM-GLTSAVAD : 2058
W97-11 : STTSPDSDSS---AANKTVRIHPFESRRSIESKLPTSTTCFLNLSSSKENVTADVINSYIPTQCRVETRDSLTALVGQITRSTSM-GLTSAVAD : 2058
B71    : STTTATSHSEADGTNKTNVRIHPLESRRSIESKLPSNATCFLDLSTSNGSEAAAVINSYIPAQCRVETRDTLTATAGQVTRSTSTGGLGPAVGD : 2067
B2     : STTTATSHSEADGTNKTNVRIHPLESRRSIESKLPSNATCFLDLSTSNGSEAAAVINSYIPAQCRVETRDTLTATACQVTRSTSTGGLGPAVGD : 2066
Ref    : STTTATSHSEADGTNKTNVRIHPLESRRSIESKLPSNATCFLDLSTNNGSEAAAVINSYIPAQCRVETRDTLTATACQVTRSTSTGGLGPAVGD : 2066
70-15  : STTTATSHSEADGTNKTNVRIHPLESRRSIESKLPSNATCFLDLSTNNGSEAAAVINSYIPAQCRVETRDTLTATACQVTRSTSTGGLGPAVGD : 2066

SCAU-2 : ILRTCWANVQAVRRDLTPFSGAVFTPTELTATVGK---MSPKVGNDALSVITDWTAEEELGVLIQPADSMVRFKQDKTYWLVGLTGGLALSLCR : 2149
NI907  : ILRTCWANVQAVRRDLTPFSGAVFTPTELTATVGK---MSPKVGNDALSVITDWTAEEELGVLIQPADSMVRFKQDKTYWLVGLTGGLALSLCR : 2149
W97-11 : ILRTCWANVQAVRRDLTPFSGAVFTPTELTATVGK---MSPKVGNDALSVITDWTAEEELGVLIQPADSMVRFKQDKTYWLVGLTGGLALSLCR : 2149
B71    : VLPACWANVEAAGRALSFFSAAVVTPTELTAAAGNGKTSAPRVGDDALLLITDWTAEAEVGVLVQPADSMVRFKQDKTYWLVGLTGGLALSLCR : 2161
B2     : VLPACWANVEAAGRDLSFFSAAVVTPTELTAAAGNGKTSAPRVGDDALLLITDWTAEAEVGVLVQPADSMVRFRQDKTYWLVGLTGGLALSLCR : 2160
Ref    : VLPACWANVEAAGRDLSFFSAAVVTPTELTAAAGNGKTSAPRVGDDALLLITDWTAEAEVGVLVQPADSMVRFRQDKTYWLVGLTGGLALSLCR : 2160
70-15  : VLPACWANVEAAGRDLSFFSAAVVTPTELTAAAGNGKTSAPRVGDDALLLITDWTAEAEVGVLVQPADSMVRFRQDKTYWLVGLTGGLALSLCR : 2160

SCAU-2 : WMVNRGARYVVMTSRNPVIDKEWLHTVESCGATVKIFSNDVTDRAAVNSAYRIISATLPPIAGVVQGAMVLRDTMFAETTMETIETVLGPKVRG : 2243
NI907  : WMVNRGARYVVMTSRNPVIDKEWLHTVESCGATVKIFSNDVTDRAAVNSAYRIISATLPPIAGVVQGAMVLRDTMFAETTMETIETVLGPKVRG : 2243
W97-11 : WMVNRGARYVVMTSRNPVIDKEWLHTVESCGATVKIFSNDVTDRAAVNSAYRIISATLPPIAGVVQGAMVLRDTMFAETTMETIETVLGPKVRG : 2243
B71    : WMVNRGARYVVMTSRNPKIDKEWLQGVESCGATVKIFSNDVTDRAAVNSAYRTISATLPPIAGVVQGAMVLRDTMFAETTMETIESILGPKVRG : 2255
B2     : WMVNRGARYVVMTSRNPKIDKEWLQGVESCGATVKIFSNDVTDRAAVNSAYRTISATLPPIAGVVQGAMVLRDTMFAETTMETIESILGPKVRG : 2254
Ref    : WMVNRGARYVVMTSRNPKIDKEWLQGVESCGATVKIFSNDVTDRAAVNSAYRTISATLPPIAGVVQGAMVLRDTMFAETTMETIESILGPKVRG : 2254
70-15  : WMVNRGARYVVMTSRNPKIDKEWLQGVESCGATVKIFSNDVTDRAAVNSAYRTISATLPPIAGVVQGAMVLRDTMFAETTMETIESILGPKVRG : 2254
```

```
SCAU-2 : SIYLDEIFYSTPLDFFVFLSSVTSTSGNPGQSIYAGANMFMNSLAAQRRKRGVAGSSVEIGCIMGNGSVTSILSYEHQKYLFSVGNTWLSEQDF : 2337
NI907  : SIYLDEIFYSTPLDFFVFLSSVTSTSGNPGQSIYAGANMFMNSLAAQRRKRGVAGSSVEIGCIMGNGSVTSILSYEHQKYLFSVGNTWLSEQDF : 2337
W97-11 : SIYLDEIFYSTPLDFFVFLSSVTSTSGNPGQSIYAGANMFMNSLAAQRRKRGVAGSSVEIGCIMGNGSVTSILSYEHQKYLFSVGNTWLSEQDF : 2337
B71    : SIYLDEIFYSTPLDFFVFLSSVTATSGNPGQSIYAGANMFMNSLAAQRRKRGVAGSSVEIGCIMGNGSVTTILSYEHQKYLFSVGNTWLAEQDF : 2349
B2     : SIYLDEIFYSTPLDFFVFLSSVTATSGNPGQSIYAGANMFMNSLAAQRRKRGVAGSSVEIGCIMGNGSVTTILSYEHQKYLFSVGNTWLAEQDF : 2348
Ref    : SIYLDEIFYSTPLDFFVFLSSVTATSGNPGQSIYAGANMFMNSLAAQRRKRGVAGSSVEIGCIMGNGSVTTILSYEHQKYLFSVGNTWLAEQDF : 2348
70-15  : SIYLDEIFYSTPLDFFVFLSSVTATSGNPGQSIYAGANMFMNSLAAQRRKRGVAGSSVEIGCIMGNGSVTTILSYEHQKYLFSVGNTWLAEQDF : 2348

SCAU-2 : LTMFGEAVLASPPDSSDSVTSVTGLRLQFNDDKPDITWFSNPIFQHLVLQSGNAMQTSLSVVRQGTPVKTLLQDAKSSEEVLEILKDAFQAKLV : 2431
NI907  : LTMFGEAVLASPPDSSDSVTSVTGLRLQFNDDKPDITWFSNPIFQHLVLQSGNAMQTSLSVVRQGTPVKTLLQDAKSSEEVLEILKDAFQAKLV : 2431
W97-11 : LTMFGEAVLASPPDSSDSVTSVTGLRLQFNDDKPDITWFSNPIFQHLVLQSGNAMQTSLSVVRQGTPVKTLLQDAKSSEEVLEILKDAFQAKLV : 2431
B71    : LTMFGEAVLASPPDAPDSVTSVTGLRLQFNDDKPDITWFSNPIFQHLVLQSGNAMQTSLSVARQGTPVKSLLQEAKSSEEVLDILKDAFTAKLV : 2443
B2     : LTMFGEAVLASPPDAPDSVTSVTGLRLQFNDDKPDITWFSNPIFQHLVLQSGNAMQTSLSVARQGTPVKSLLQEAKSSEEVLDILKDAFTAKLV : 2442
Ref    : LTMFGEAVLASPPDAPDSVTSVTGLRLQFNDDKPDITWFSNPIFQHLVLQSGNAMQTSLSVARQGTPVKSLLQEAKSSEEVLDILKDAFTAKLV : 2442
70-15  : LTMFGEAVLASPPDAPDSVTSVTGLRLQFNDDKPDITWFSNPIFQHLVLQSGNAMQTSLSVARQGTPVKSLLQEAKSSEEVLDILKDAFTAKLV : 2442

SCAU-2 : SSLQADPDSNILEVDLETLGMDSLVAVDLRSWFLAELSVDVPVLKILNGSTARSLLEFVQGLIPASMTPKLDGLDGADTA-----PQQAPPVTK : 2520
NI907  : SSLQADPDSNILEVDLETLGMDSLVAVDLRSWFLAELSVDVPVLKILNGSTARSLLEFVQGLIPASMTPKLDGLDGADTA-----PQQAPPVTK : 2520
W97-11 : SSLQADPDSNILEVDLETLGMDSLVAVDLRSWFLAELSVDVPVLKILNGSTARSLLEFVQGLIPASMTPKLDGLDGADTA-----PQQAPPVTK : 2520
B71    : SSLQADPDSNLLEVDLETLGMDSLVAVDLRSWFLAELSVDVPVLKILNGSTARLLLEFVQGLIPASMTPKLDGSDGADAAAQEEAPPVAPPVTK : 2537
B2     : SSLQADPDSNLLEVDLETLGMDSLVAVDLRSWFLAELSVDVPVLKILNGSTARLLLEFVQGLIPASMTPKLDGSDGADAAAQ-EAPPVAPPVTK : 2535
Ref    : SSLQADPDSNLLEVDLETLGMDSLVAVDLRSWFLAELSVDVPVLKILNGSTARLLLEFVQGLIPASMTPKLDGSDGADAAAQ-EAPPVAPPVTK : 2535
70-15  : SSLQADPDSNLLEVDLETLGMDSLVAVDLRSWFLAELSVDVPVLKILNGSTARLLLEFVQGLIPASMTPKLDGSDGADAAAQ-EAPPVAPPVTK : 2535

SCAU-2 : TQPEVSIKLPGSVSNYPPVASIKQSDSASSSSPSPEARSPDQPRSVASSMTDDRLDLSTPTTSASFASLDDSRKLIRTVPVSFGQARFWFLRSY : 2614
NI907  : TQPEVSIKLPGSVSNYPPVASIKQSDSASSSSPSPEARSPDQPRSVASSMTDDRLDLSTPTTSASFASLDDSRKLIRTVPVSFGQARFWFLRSY : 2614
W97-11 : TQPEVSIKLPGSVSNYPPVASIKQSDSASSSSPSPEARSPDQPRSVASSMTDDRLDLSTPTTSASFASLDDSRKLIRTVPVSFGQARFWFLRSY : 2614
B71    : PKPDVSVKVPPP---HQPVASLKPSGPASPTSPSSATASPGRSRSVASPVTADT--PVSPTTSASMASLNDSRKLIRTVPVSFGQSRFWFLGSY : 2626
B2     : PKPDVSVKVPPP---HQPVASLKPSGPASPTSPSSATASPGRSRSVASPVTADT--PVSPTTSASMASLNDSRKLIRTVPVSFGQSRFWFLGSY : 2624
Ref    : PKPDVSVKVPPP---HQPVASLKPSGPASPTSPSSATASPGRSRSVASPVTADT--PVSPTTSASMASLNDSRKLIRTVPVSFGQSRFWFLGSY : 2624
70-15  : PKPDVSVKVPPP---HQPVASLKPSGPASPTSPSSATASPGRSRSVASPVTADT--PVSPTTSASMASLNDSRKLIRTVPVSFGQSRFWFLGSY : 2624
```

```
SCAU-2 : NPDPLAFNITSLMRITGPLRTPDFAKAVERVLNHHEALRTSFVEEDNGPMQKIWSSPAFGLEQRKIIDDDSAVVKACKDVQNTVYNLDQGQTMR : 2708
NI907  : NPDPLAFNITSLMRITGPLRTPDFAKAVERVLNHHEALRTSFVEEDNGPMQKIWSSPAFGLEQRKIIDDDSAVVKACKDVQNTVYNLDQGQTMR : 2708
W97-11 : NPDPLAFNITSLMRITGPLRTPDFAKAVERVLNHHEALRTSFVEEDNGPMQKIWSSPAFGLEQRKIIDDDSAVVKACKDVQNTVYNLDQGQTMR : 2708
B71    : NPDPLAFNITSLMRISGPLRTNDFAKAVDKVLNHHEALRTSFVSENDAPVQKIWSSPAFTLEQRKIADDESEVVKAYTEVQNTRYNLEAGQTMR : 2720
B2     : NPDPLAFNITSLMRISGPLRTNDFGKAVDKVLNHHEALRTSFVSENDAPVQKIWSSPAFALEQRKIADDESEVFKAYTEVQNTRYNLEAGQTMR : 2718
Ref    : NPDPLAFNITSLMRISGPLRTNDFGKAVDKVLNHHEALRTSFVSENDAPVQKIWSSPAFALEQRKIADDESEVVKAYTEVQNTRYNLEAGQTMR : 2718
70-15  : NPDPLAFNITSLMRISGPLRTNDFGKAVDKVLNHHEALRTSFVSENDAPVQKIWSSPAFALEQRKIADDESEVVKAYTEVQNTRYNLEAGQTMR : 2718

SCAU-2 : ILLLTKSPTQHVLVLGYHHINMDGVSFEVLFSDIEKAYNGLPLDRSVMQFPDFTIKEFNEFKSGGWESELQYWRSKFTSLPEATPLLSVSKRRS : 2802
NI907  : ILLLTKSPTQHVLVLGYHHINMDGVSFEVLFSDIEKAYNGLPLDRSVMQFPDFTIKEFNEFKSGGWESELQYWRSKFTSLPEATPLLSVSKRRS : 2802
W97-11 : ILLLTKSPTQHVLVLGYHHINMDGVSFEVLFSDIEKAYNGLPLDRSVMQFPDFTIKEFNEFKSGGWESELQYWRSKFTSLPEATPLLSVSKRRS : 2802
B71    : IMLLTKSPTKHVLVLGYHHINMDGVSFEVLFSDIEKAYNRTPLDRSVMQFPDFTIREAGEYKSGAWRSELQYWQSKFTSLPEPTPLLSVSKRRT : 2814
B2     : IMLLTKSPTKHVLVLGYHHINMDGVSFEVLFSDIEKAYNRTPLDRSVMQFPDFTIREAGEYKSGAWRSELQYWQSKFTSLPEPTPLLSVSKRRT : 2812
Ref    : IMLLTKSPTKHVLVLGYHHINMDGVSFEVLFSDIEKAYNRTPLDRSVMQFPDFTIREAGEYKSGAWRSELQYWQSKFTSLPEPTPLLSVSKRRT : 2812
70-15  : IMLLTKSPTKHVLVLGYHHINMDGVSFEVLFSDIEKAYNRTPLDRSVMQFPDFTIREAGEYKSGAWRSELQYWQSKFTSLPEPTPLLSVSKRRT : 2812

SCAU-2 : RPINLSYTTHSINRRISAEQSKAIQSVSRKFKATPFHFYLAVFKTLIARFSGTDDFCIGIADANRKEEKVMAAVGLYLNLLPLRVRSSLAQTFG : 2896
NI907  : RPINLSYTTHSINRRISAEQSKAIQSVSRKFKATPFHFYLAVFKTLIARFSGTDDFCIGIADANRKEEKVMAAVGLYLNLLPLRVRSSLAQTFG : 2896
W97-11 : RPINLSYTTHSINRRISAEQSKAIQSVSRKFKATPFHFYLAVFKTLIARFSGTDDFCIGIADANRKEEKVMAAVGLYLNLLPLRVRSSLAQTFG : 2896
B71    : RPVNLSYTTHSVSRRINAEQSQAIHTVGRKFKATPFHFYLSVFKTLIARFSGADDFCIGIADANRKEDKVMGAVGLYLNLLPLRVRSALGQTFG : 2908
B2     : RPVNLSYTTHSVSRRINAEQSQAIHTVGRKFKATPFHFYLSVFKTLIARFSGADDFCIGIADANRKEDKVMGAVGLYLNLLPLRVRSALGQTFG : 2906
Ref    : RPVNLSYTTHSVSRRINAEQSQAIHTVGRKFKATPFHFYLSVFKTLIARFSGADDFCIGIADANRKEDKVMGAVGLYLNLLPLRVRSALGQTFG : 2906
70-15  : RPVNLSYTTHSVSRRINAEQSQAIHTVGRKFKATPFHFYLSVFKTLIARFSGADDFCIGIADANRKEDKVMGAVGLYLNLLPLRVRSALGQTFG : 2906

SCAU-2 : EALVDMKRVSQEAFANSKVPFDVLLNELNVPRSSSHTPLFQTFVNYRRGISEERSFCGCTGAGELISGGQVGYDISLDVVENPDGDALVTLSVQ : 2990
NI907  : EALVDMKRVSQEAFANSKVPFDVLLNELNVPRSSSHTPLFQTFVNYRRGISEERSFCGCTGAGELISGGQVGYDISLDVVENPDGDALVTLSVQ : 2990
W97-11 : EALVDMKRVSQEAFANSKVPFDVLLNELNVPRSSSHTPLFQTFVNYRRGISEERSFCGCTGAGELISGGQVGYDISLDVVENPDGDALVTLSVQ : 2990
B71    : ETLADMKKVSQEAFANSKVPFDVLLNELSVPRSSSQTPLFQTFVNYRRGVSEERSFCGCTGAGELISGGQIGYDISLDIVENPGGDALVTLSVQ : 3002
B2     : ETLADMKKVSQEAFANSKVPFDVLLNELSVPRSSSHTPLFQTFVNYRRGVSEERSFCGCTGAGELISGGQIGYDISLDIVENPGGDALVTLSVQ : 3000
Ref    : ETLADMKKVSQEAFANSKVPFDVLLNELSVPRSSSQTPLFQTFVNYRRGVSEERSFCGCTGAGELISGGQIGYDISLDIVENPGGDALVTLSVQ : 3000
70-15  : ETLADMKKVSQEAFANSKVPFDVLLNELSVPRSSSQTPLFQTFVNYRRGVSEERSFCGCTGAGELISGGQIGYDISLDIVENPGGDALVTLSVQ : 3000
```

```
SCAU-2 : KDLYDMDMANLLFDSYFRLVDSFSKNPATSLNRPALYDPVAVKKALELGCGPS-QDLSWPETLVHRIEDMSVRYATKFALRNGQNAGLTYARMI : 3083
NI907  : KDLYDMDMANLLFDSYFRLVDSFSKNPATSLNRPALYDPVAVKKALELGCGPS-QDLSWPETLVHRIEDMSVRYATKFALRNGQNAGLTYARMI : 3083
W97-11 : KDLYDMDMANLLFDSYFRLVDSFSKNPATSLNRPALYDPVAVKKALELGCGPS-QDLSWPETLVHRIEDMSVRYATKFALRNGQNAGLTYARMI : 3083
B71    : KDLYNVDMANLLLDSYFRLVDSFAKNPATSLNRPAIYDPVAVDKALTLGCGPTLEDSSWPETLIHRIENMSVKYATKFALRNGQNGGLTYSQMI : 3096
B2     : KDLYNVDMANLLLDSYFRLVDSFAKNPATSLNRPAIYDPVAVDKALTLGCGPTLEDSSWPETLIHRIENMSVKYATKFALRNGQNGGLTYSQMI : 3094
Ref    : KDLYNVDMANLLLDSYFRLVDSFAKNPATSLNRPAIYDPVAVDKALTLGCGPTLEDSSWPETLIHRIENMSVKYATKFALRNGQNGGLTYSQMI : 3094
70-15  : KDLYNVDMANLLLDSYFRLVDSFAKNPATSLNRPAIYDPVAVDKALTLGCGPTLEDSSWPETLIHRIENMSVKYATKFALRNGQNGGLTYSQMI : 3094

SCAU-2 : ARVNDIAAKLIKAMVGSNPGIVGVMQASTMDFICSILAIWKAGAIYTPLDPRLNSVDRLRAVVDECQPICILVDATTKPLFDSLSSKAIQIDVS : 3177
NI907  : ARVNDIAAKLIKAMVGSNPGIVGVMQASTMDFICSILAIWKAGAIYTPLDPRLNSVDRLRAVVDECQPICILVDATTKPLFDSLSSKAIQIDVS : 3177
W97-11 : ARVNDIAAKLIKAMVGSNPGIVGVMQASTMDFICSILAIWKAGAIYTPLDPRLNSVDRLRAVVDECQPICILVDATTKPLFDSLSSKAIQIDVS : 3177
B71    : ARINDIAAKLIDAKVG--SGIVGVMQASTMDFICSILAVWKAGAIYTPLDPRLNSTDRLKAVVDECQPACILVDATTKPLFDSLATNAVQIDVS : 3188
B2     : ARINDIAAKLIDAKVG--TGIVGVMQASTMDFICSILAVWKAGAIYTPLDPRLNSTDRLKAVVDECQPACILVDATTKPLFDSLATNAVQIDVS : 3186
Ref    : ARINDIAAKLIDAKVG--TGIVGVMQASTMDFICSILAVWKAGAIYTPLDPRLNSTDRLKAVVDECQPACILVDATTKPLFDSLATNAVQIDVS : 3186
70-15  : ARINDIAAKLIDAKVG--TGIVGVMQASTMDFICSILAVWKAGAIYTPLDPRLNSTDRLKAVVDECQPACILVDATTKPLFDSLATNAVQIDVS : 3186

SCAU-2 : EVQSSKTLEQSPKLAIQAKGASAAAVFYTSGSTGTPKGISLSHTSLTYNIMAATQQFGFKEGIDIMLQQSSFSFDMSLAQMLTSLSNGGTLVVV : 3271
NI907  : EVQSSKTLEQSPKLAIQAKGASAAAVFYTSGSTGTPKGISLSHTSLTYNIMAATQQFGFKEGIDIMLQQSSFSFDMSLAQMLTSLSNGGTLVVV : 3271
W97-11 : EVQSSKTLEQSPKLAIQAKGASAAAVFYTSGSTGTPKGISLSHTSLTYNIMAATQQFGFKEGIDIMLQQSSFSFDMSLAQMLTSLSNGGTLVVV : 3271
B71    : MVQSSKTLEASPKVAIHAKAPSAAAVFYTSGSTGVPKGITLSHASLTYNIMAATQQFGFKEGVDIMLQQSSFSFDMALAQMLTSLSNGGTLVVV : 3282
B2     : MVQSSKTLEASPKVAIHAKAPSAAAVFYTSGSTGVPKGITLSHASLTYNIMAATQQFGFKEGVDIMLQQSSFSFDMALAQMLTSLSNGGTLVVV : 3280
Ref    : MVQSSKTLEASPKVAIHAKAPSAAAVFYTSGSTGVPKGITLSHASLTYNIMAATRQFGFKEGVDIMLQQSSFSFDMALAQMLTSLSNGGTLVVV : 3280
70-15  : MVQSSKTLEASPKVAIHAKAPSAAAVFYTSGSTGVPKGITLSHASLTYNIMAATRQFGFKEGVDIMLQQSSFSFDMALAQMLTSLSNGGTLVVV : 3280

SCAU-2 : PSHLRGDALGLSQLIVAENVSIVQASPTEYKSLIGVNAQQLRTSKWRVALSGGENMTQNLLEVFRSLGKPDLVLYNGYGPTEATINANTRIIPY : 3365
NI907  : PSHLRGDALGLSQLIVAENVSIVQASPTEYKSLIGVNAQQLRTSKWRVALSGGENMTQNLLEVFRSLGKPDLVLYNGYGPTEATINANTRIIPY : 3365
W97-11 : PSHLRGDALGLSQLIVAENVSIVQASPTEYKSLIGVNAQQLRTSKWRVALSGGENMTQNLLEVFRSLGKPDLVLYNGYGPTEATINANTRIIPY : 3365
B71    : PSHLRGDALGLSQLIVAENVSIVQASPTEYKSLIGVNAQHLKTSKWRVALSGGENMTQSLLEVFRSLGKPDLVLFNGYGPTEATINANTRIVPY : 3376
B2     : PSHLRGDALGLSQLIVAENVSIVQASPTEYKSLIGVNAQHLKTSKWRVALSGGENMTQSLLEVFRSLGKPDLVLFNGYGPTEATINANTRIVPY : 3374
Ref    : PSHLRGDALGLSQLIVAENVSIVQASPTEYKSLIGVNAQHLKTSKWRVALSGGENMTQSLLEVFRSLGKPDLVLFNGYGPTEATINANTRIVPY : 3374
70-15  : PSHLRGDALGLSQLIVAENVSIVQASPTEYKSLIGVNAQHLKTSKWRVALSGGENMTQSLLEVFRSLGKPDLVLFNGYGPTEATINANTRIVPY : 3374
```

```
SCAU-2 : HEPNSNPDLPLLTWENYSISVVDLELNPVPVGVFGEICIGGAGVGLGYFKNEELTSKAFVADKTAPPEFLAKGWKTKYRTGDLGRLSPDGGLII : 3459
NI907  : HEPNSNPDLPLLTWENYSISVVDLELNPVPVGVFGEICIGGAGVGLGYFKNEELTSKAFVADKTAPPEFLAKGWKTKYRTGDLGRISPDGGLII : 3459
W97-11 : HEPNSNPDLPLLTWENYSISVVDLELNPVPVGVFGEICIGGAGVGLGYFKNEELTSKAFVADKTAPPEFLAKGWKTKYRTGDLGRLSPDGGLII : 3459
B71    : HEPNSNPDLPLLTWPNYSISIVDLELNPVPVGVFGEVCIGGAGVGLGYFKNDELTAKAFVADKTAPAEFVAKGWKTKFRTGDLGRLSPDGGLII : 3470
B2     : HEPNSNPDLPLLTWPNYSISIVDLELNPVPVGVFGEVCIGGAGVGLGYFKNDELTAKAFVADKTAPAEFVAKGWKTKFRTGDLGRLSPDGGLII : 3468
Ref    : HEPNSNPDLPLLTWPNYSISIVDLELNPVPVGVFGEVCIGGAGVGLGYFKNDELTAKAFVADKTAPAEFVAKGWKTKFRTGDLGRLSPDGGLII : 3468
70-15  : HEPNSNPDLPLLTWPNYSISIVDLELNPVPVGVFGEVCIGGAGVGLGYFKNDELTAKAFVADKTAPAEFVAKGWKTKFRTGDLGRLSPDGGLII : 3468

SCAU-2 : EGRIDGDTQIKLRGIRIDLQNVESAILEAGAGKIIDVAVSLRRGGADESDPQYLVGHVVLDSDQIPQNSQQEFLAQIVPRLRLPQHMKPSLLVP : 3553
NI907  : EGRIDGDTQIKLRGIRIDLQNVESAILEAGAGKIIDVAVSLRRGGADESDPQYLVGHVVLDSDQIPQNSQQEFLAQIVPRLRLPQHMKPSLLVP : 3553
W97-11 : EGRIDGDTQIKLRGIRIDLQNVESAILEAGAGKIIDVAVSLRRGGADESDPQYLVGHVVLDSDQIPQNSQQEFLAQIVPRLRLPQHMKPSLLVP : 3553
B71    : EGRIDGDTQVKLRGMRIDLKNIESAILQAGAGKIIDAAVSVRRGGADESEPQYLVGHVVLDADQTPEDSQQDFLAQLIPRLRLPRHMKPSLLVP : 3564
B2     : EGRIDGDTQVKLRGMRIDLKNIESAILQAGAGKIIDAAVSVRRGGADESEPQYLVGHVVLDADQTPEDSQQDFLAQLIPRLRLPRHMKPSLLVP : 3562
Ref    : EGRIDGDTQVKLRGMRIDLKNIESAILQAGAGKIIDAAVSVRRGGADESEPQYLVGHVVLDADQTPEDSQQDFLAQLIPRLRLPRHMKPSLLVP : 3562
70-15  : EGRIDGDTQVKLRGMRIDLKNIESAILQAGAGKIIDAAVSVRRGGADESEPQYLVGHVVLDADQTPEDSQQDFLAQLIPRLRLPRHMKPSLLVP : 3562

SCAU-2 : IHSLPQTASHKLDRRALQDLPISDENQIV---HQGAELGSDQAEMWKLWKQVIPSDVSSKYSITPRSDFFHVGGTSLLLVNLQSLIRKKHGSAP : 3644
NI907  : IHSLPQTASHKLDRRALQDLPISDENQIV---HQGAELGSDQAEMWKLWKQVIPSDVSSKYSITPRSDFFHVGGTSLLLVNLQSLIRKKHGSAP : 3644
W97-11 : IHSLPQTASHKLDRRALQDLPISDENQIV---HQGAELGSDQAEMWKLWKQVIPSDVSSKYSITPRSDFFHVGGTSLLLVNLQSLIRKKHGSAP : 3644
B71    : IRALPQTASHKLDRRALQQLPISDAGQIAKQSQQGAELGSDQARMWKLWKQVIPRDVVSQYSITPQSDFFHVGGTSLLLVNLQSLIAREHGRAP : 3658
B2     : IRALPQTASHKLDRRALQQLPISDAGQIAKQSQQGAELGSDQARMWKLWKQVIPRDVVSQYSITPQSDFFHVGGTSLLLVNLQSLIAREHGRAP : 3656
Ref    : IRALPQTASHKLDRRALQQLPISDAGQIAKQSQQGAELGSDQARMWKLWKQVIPRDVVSQYSITPQSDFFHVGGTSLLLVNLQSLIAREHGRAP : 3656
70-15  : IRALPQTASHKLDRRALQQLPISDAGQIAKQSQQGAELGSDQARMWKLWKQVIPRDVVSQYSITPQSDFFHVGGTSLLLVNLQSLIAREHGRAP : 3656

SCAU-2 : PLHAMFESSTVASMTDLVLSDNPSGNGALIDWEQETSIPMLAPNVIPGGGANKVSLPPRVVLITGATGFLGRQLVEFLLRQPNITRIHCLAVRN : 3738
NI907  : PLHAMFESSTVASMTDLVLSDNPSGNGALIDWEQETSIPMLAPNVIPGGGANKVSLPPRVVLITGATGFLGRQLVEFLLRQPNITRIHCLAVRN : 3738
W97-11 : PLHAMFESSTVASMTDLVLSDNPSGNGALIDWEQETSIPMLAPNVIPGGGANKVSLPPRVVLITGATGFLGRQLVEFLLRQPNITRIHCLAVRN : 3738
B71    : PLHAMFESSTVAAMTDLVLSDDASGSTALIDWEQETSIPTLPPHIIPGGAGNKVSVPPRVVLLTGATGFLGRQLMAFLLRQPSVKRIHCLAVRG : 3752
B2     : PLHAMFESSTVAAMTDLVLSDDASGSTALIDWEQETSIPTLPPHIIPGGAGNKVSVPPRVVLLTGATGFLGRQLMAFLLRQPSVKRIHCLAVRG : 3750
Ref    : PLHAMFESSTVAAMTDLVLSDDASGSTALIDWEQETSIPTLPPHIIPGGAGNKVSVPPRVVLLTGATGFLGRQLMAFLLRQPSVKRIHCLAVRG : 3750
70-15  : PLHAMFESSTVAAMTDLVLSDDASGSTALIDWEQETSIPTLPPHIIPGGAGNKVSVPPRVVLLTGATGFLGRQLMAFLLRQPSVKRIHCLAVRG : 3750
```

```
SCAU-2 : S-PPSSELPFSDPRVSIHHGDLSAPRLGLGEDVAESLFAEADVIIHNGADVSFLKTYASLRPVNVGSTQELARLAAPRRIPFHFVSSASITQLT : 3831
NI907  : S-PPSSELPFSDPRVSIHHGDLSAPRLGLGEDVAESLFAEADVIIHNGADVSFLKTYASLRPVNVGSTQELARLAAPRRIPFHFVSSASITQLT : 3831
W97-11 : S-PPSSELPFSDPRVSIHHGDLSAPRLGLGEDVAESLFAEADVIIHNGADVSFLKTYASLRPVNVGSTQELARLAAPRRIPFHFVSSASITQLT : 3831
B71    : AAPPSSAAPFSDPRVSIHAGDLNAPHLGLGEAVAESLFAQADVIIHNGADVSFLKTYATLRATNVGSTRELARLAAPRRIPFHFVSSASITQLT : 3846
B2     : GAPPSSAAPFSDPRVSIHAGDLNAPHLGLGEAVAESLFAQADVIIHNGADVSFLKTYATLRATNVGSTRELARLAAPRRIPFHFVSSASITQLT : 3844
Ref    : GAPPSSAAPFSDPRVSIHAGDLNAPHLGLGEAVAELLFAQADVIIHNGADVSFLKTYATLRATNVGSTRELARLAAPRRIPFHFVSSASITQLT : 3844
70-15  : GAPPSSAAPFSDPRVSIHAGDLNAPHLGLGEAVAELLFAQADVIIHNGADVSFLKTYATLRATNVGSTRELARLAAPRRIPFHFVSSASITQLT : 3844

SCAU-2 : GKDEFGEASLAAWAPPADPRAMMGGYVAAKWASEVLLEKAARAWGLPVVIHRPSSITGQNANSLDLMGNMFKYIELLEAVPESDSWKGNFDFVS : 3925
NI907  : GKDEFGEASLAAWAPPADPRAMMGGYVAAKWASEVLLEKAARAWGLPVVIHRPSSITGQNANSLDLMGNMFKYIELLEAVPESDSWKGNFDFVS : 3925
W97-11 : GKDEFGEASLAAWAPPADPRAIMGGYVAAKWASEVLLEKAARAWGLPVVIHRPSSITGQNANSLDLMGNMFKYIELLEAVPESDSWKGNFDFVS : 3925
B71    : GLDEFGEASMAAWAPPTDPRAMSGGYAAAKWASEVLLEKAARAWGLPVVIHRPSSITGEGTNSLDLMGNMFKYIEQLEAVPESDSWKGNFDFVS : 3940
B2     : GLDEFGEASMAAWAPPADPRGMSGGYAAAKWASEVLLEKAARAWGLPVVIHRPSSITGEGTNSLDLMGNMFKYIEQLEAVPESDSWKGNFDFVS : 3938
Ref    : GLDEFGEASMAAWAPPADPRGMSGGYAAAKWASEVLLEKAARAWGLPVVIHRPSSITGEGTNSLDLMGNMFKYIEQLEAVPESDSWKGNFDFVS : 3938
70-15  : GLDEFGEASMAAWAPPADPRGMSGGYAAAKWASEVLLEKAARAWGLPVVIHRPSSITGEGTNSLDLMGNMFKYIEQLEAVPESDSWKGNFDFVS : 3938

SCAU-2 : VENVAADVVQAVVAANAAASGGVKYIYEAGDIIYPLSMVKDMSEGGAELPVKTIPLAQWVKQAAEMGLDAMLAEYLLKAAKTGTVLAFPKLLKN : 4019
NI907  : VENVAADVVQAVVAANAAASGGVKYIYEAGDIIYPLSMVKDMSEGGAELPVKTIPLAQWVKQAAEMGLDAMLAEYLLKAAKTGTVLAFPKLLKN : 4019
W97-11 : VENVAADVVQAVVAANAAASGGVKYIYEAGDIIYPLSMVKDMSEGGAELPVKTIPLAQWVKQAAEMGLDAMLAEYLLKAAKTGTVLAFPKLLKN : 4019
B71    : VENVAADIVQAVVAANVVAAGGVKFIYEAGDIVYPLSMVKDMSEGGAKLPVKTMPLAKWVEKAAEKGLDSMLAEYLIKAASTGTSLAFPRLLKD : 4034
B2     : VENVAADIVQAVVAANVVAAGGVKFIYEAGDIVYPLSMVKDMSEGGAKLPVKTMPLAKWVEKAAEKGLDSMLAEYLIKAASTGTSLAFPRLLKD : 4032
Ref    : VENVAADIVQAVVAANVVAAGGVKFIYEAGDIVYPLSMVKDMSEGGAKLPVKTMPLAKWVEKAAEKGLDSMLAEYLIKAASTGTSLAFPRLLKD : 4032
70-15  : VENVAADIVQAVVAANVVAAGGVKFIYEAGDIVYPLSMVKDMSEGGAKLPVKTMPLAKWVEKAAEKGLDSMLAEYLIKAASTGTSLAFPRLLKD : 4032

SCAU-2 : GQRLV : 4024
NI907  : GQRLV : 4024
W97-11 : GQRLV : 4024
B71    : GN--- : 4036
B2     : GN--- : 4034
Ref    : GN--- : 4034
70-15  : GN--- : 4034
```

Figure S7. Amino acid sequence alignment of ACE1. The sequence of referenced protein is accessible in NCBI under no. CAG28797. Sequence alignment was performed by Clustal X2 [49] and shaded by GeneDoc software [50]. The boxes with white character and black background represent that the conserved percentage is 100% in the column. The boxes with black character represent the difference of ACE1 in different *Pyricularia* strains. Among the boxes with black character, the boxes with blue background represent that the conserved percentage is higher than or equal to 80% but less than 100% in the column, while the boxes with grey background represent the conserved percentage is higher than or equal to 50% but less than 80% in the column, and the boxes with white background represent the conserved percentage is less than 50% in the column.

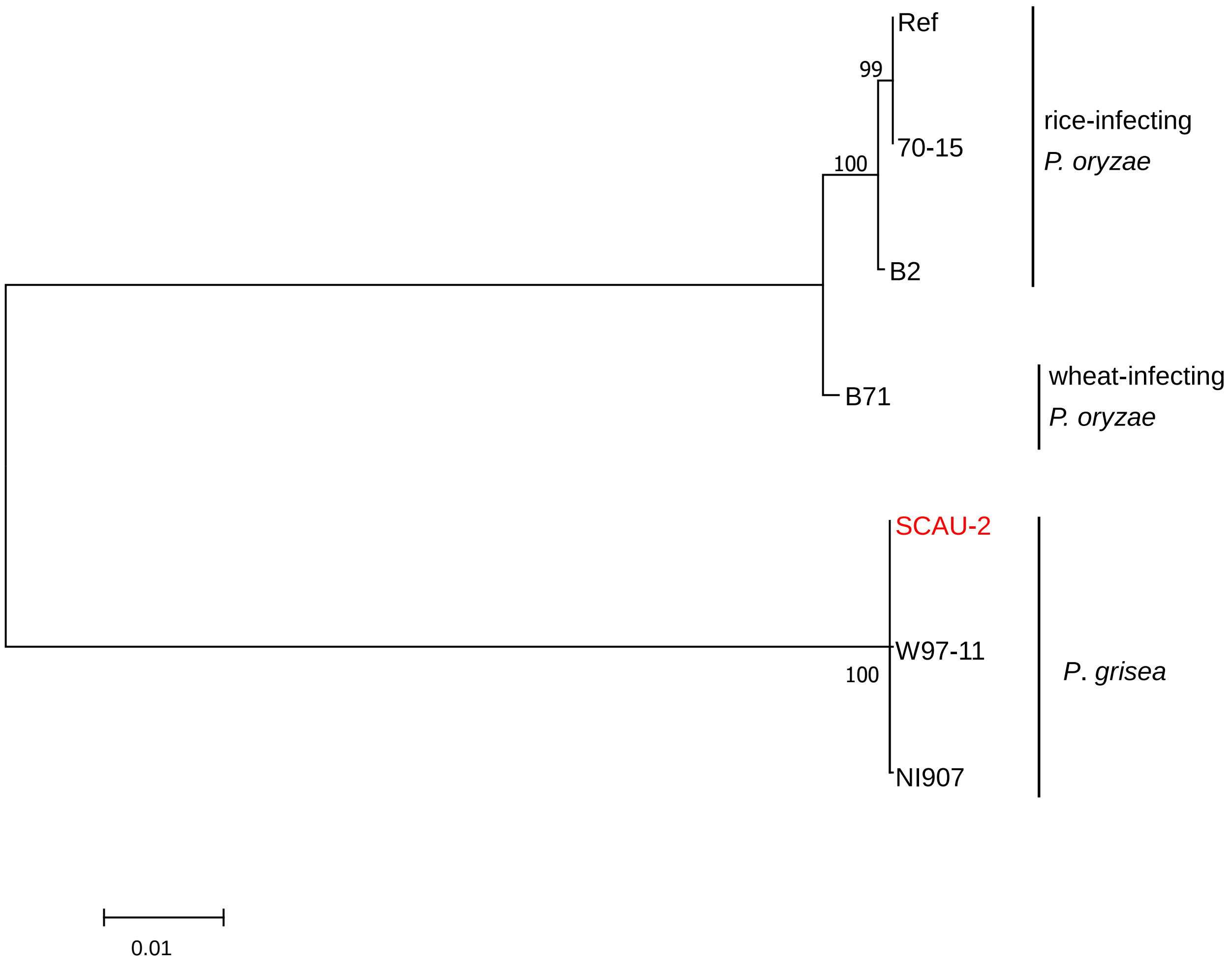

Figure S8. The phylogenic tree analysis of ACE1. The sequence of referenced protein is accessible in NCBI under no. CAG28797. Sequence alignment was performed by Clustal X2 [49]. The phylogenetic tree was constructed using the Maximum Likelihood method based on the best-fit JTT matrix-based model selected by MEGA 7 [51], with 1,000 bootstrap support. The percentage of trees is shown next to the branches.

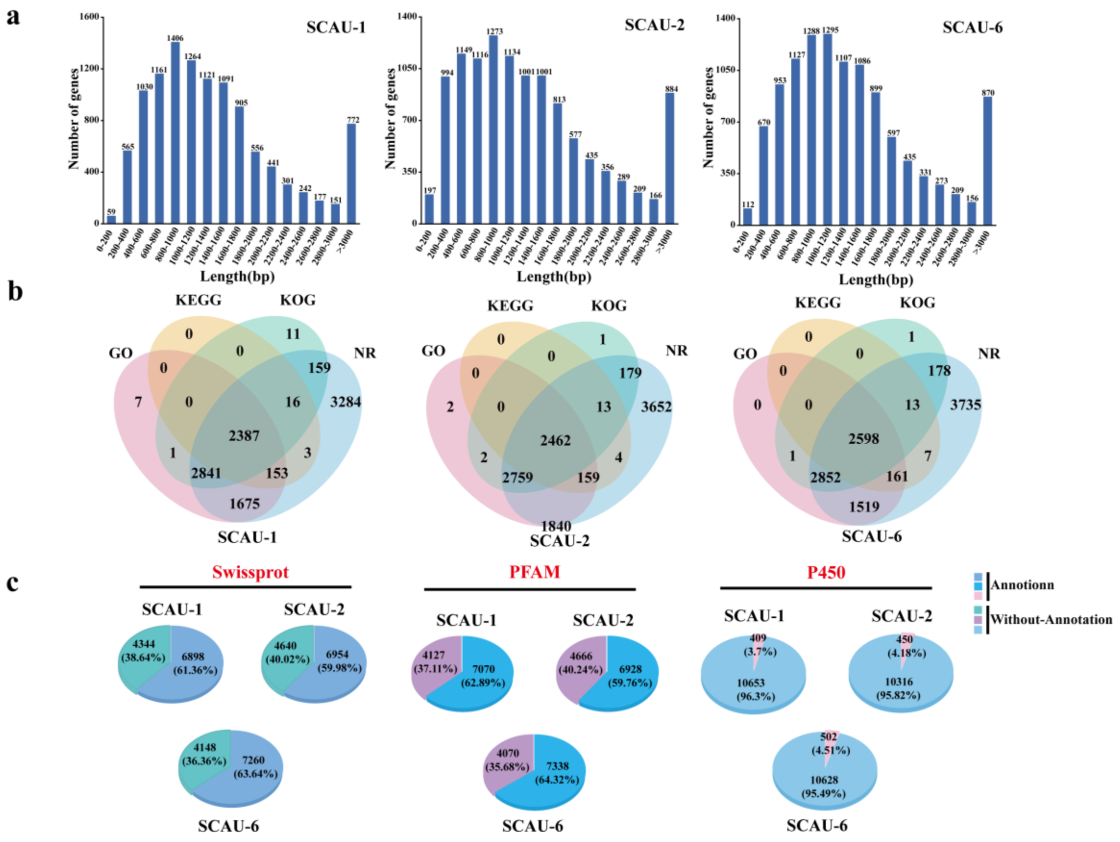

Figure S9. Distribution of gene lengths and annotations in general databases for the isolated strains. (**a**) Gene length distribution diagram of the three pathogenic strains. (**b**) Venn diagram showing the gene annotation of the pathogens in the non-redundant protein database (NR), Clusters of Eukaryotic Orthologous Groups (KOG), Gene Ontology (GO), and Kyoto Encyclopedia of Genes and Genomes (KEGG) databases. (**c**) The proportion of coding genes annotated in the Pfam, Swiss-Prot, and Cytochrome P450 databases among the three strains.

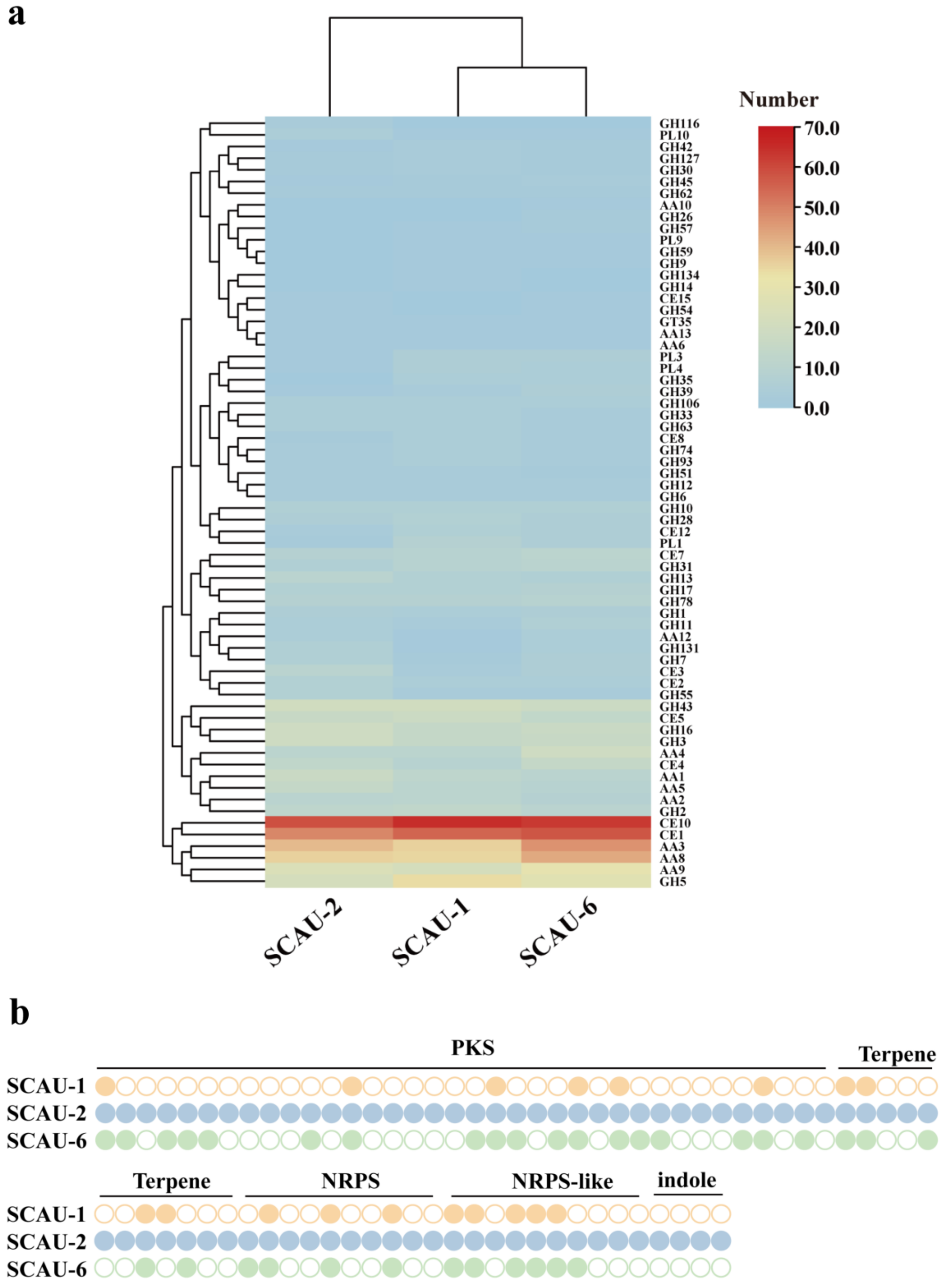

Figure S10. Cluster analysis of plant cell wall degrading enzymes (FCWDEs) and the presence/absence of core genes for secondary metabolite biosynthesis in three pathogens. (**a**) Cluster analysis of FCWDEs of three pathogens. The color changed from blue to red, indicating that the number of enzymes increased from low to high. (**b**)

Visualization of the presence/absence of the secondary metabolite biosynthetic core genes of SCAU-1 and SCAU-6 in SCAU-2. Blank indicates absence, and different colors represent the presence of core genes of secondary metabolites in different strains.
